# Synthesis of highly functionalized oligobenzamide proteomimetic foldamers by late stage introduction of sensitive groups†
†Electronic supplementary information (ESI) available. See DOI: 10.1039/c6ob00078a



**DOI:** 10.1039/c6ob00078a

**Published:** 2016-03-23

**Authors:** George M. Burslem, Hannah F. Kyle, Panchami Prabhakaran, Alexander L. Breeze, Thomas A. Edwards, Stuart L. Warriner, Adam Nelson, Andrew J. Wilson

**Affiliations:** a School of Chemistry , University of Leeds , Woodhouse Lane , Leeds , LS29JT , UK . Email: a.j.wilson@leeds.ac.uk; b Astbury Centre for Structural Molecular Biology , University of Leeds , Woodhouse Lane , Leeds , LS29JT , UK; c School of Molecular and Cellular Biology , Faculty of Biological Sciences , University of Leeds , Woodhouse Lane , Leeds LS2 9JT , UK; d Discovery Sciences , AstraZeneca R&D , Alderley Park , Cheshire , SK10 4TG , UK

## Abstract

α-Helix proteomimetics represent an emerging class of ligands that can be used to inhibit an array of helix mediated protein–protein interactions. Within this class of inhibitor, aromatic oligobenzamide foldamers have been widely and successfully used. This manuscript describes alternative syntheses of these compounds that can be used to access mimetics that are challenging to synthesize using previously described methodologies, permitting access to compounds functionalized with multiple sensitive side chains and accelerated library assembly through late stage derivatisation.

## Abstract 2

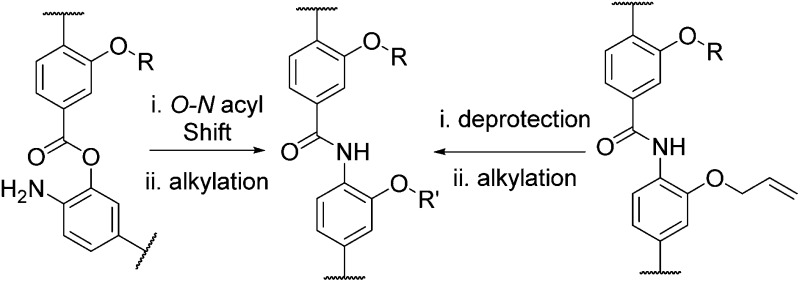
New routes are developed to allow introduction of sensitive side-chains and synthesis of challenging proteomimetic sequences.

## Introduction

**Fig. 1:**
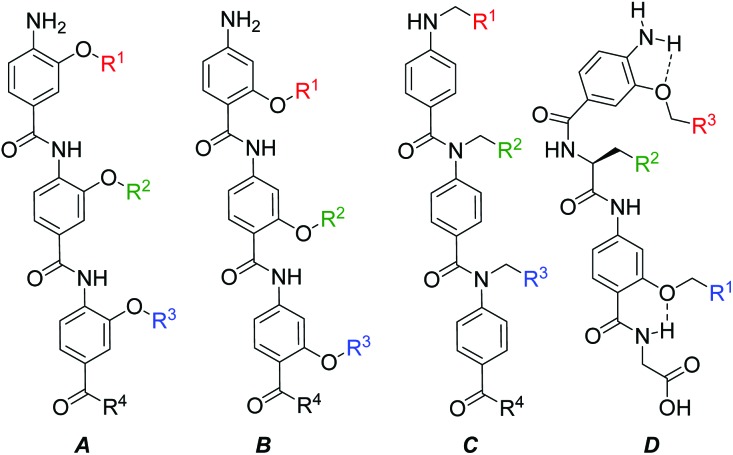
Structures of aromatic oligoamide helix mimetics **A–D**.

**Scheme 1:**
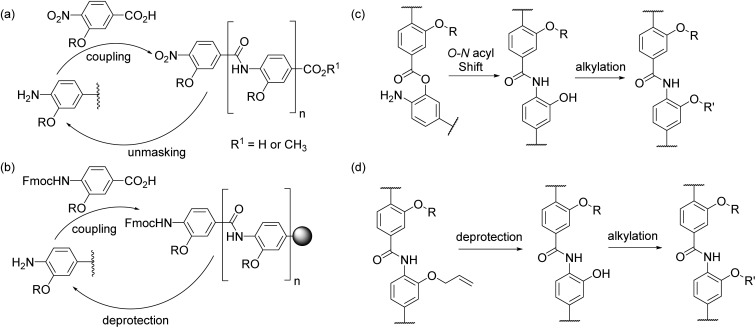
General strategies for aromatic oligoamide helix mimetics synthesis (a) *N*-terminal elongation with nitro-functionalised monomers (b) solid-phase synthesis using Fmoc strategy (c) *O*–*N*-acyl-shift for post monomer coupling functional group introduction (d) late stage functionalization.

An on-going challenge in chemical biology is the inhibition of protein–protein interactions (PPIs) using designed small molecules.^1,2^ An approach to the targeting of PPIs that has gained attention is the use of secondary structure mimics^3,4^ which have been referred to as α-helix mimetics, proteomimetics,^5^ topographical mimics and pharmacological chaperones.^6^ Oligobenzamide helix mimetics (*e.g.* **A–D** Fig. 1) have been shown by us and others to act as effective and selective inhibitors of a range of PPIs.^7–20^ Our group has developed two general approaches for synthesis^21^ of these compounds (Scheme 1a and b): (i) iterative solution phase coupling of nitro-masked monomers with carboxy-protected terminal anilines, followed by unmasking of the latent amine^10,12,13,22–24^ and (ii) *N*-terminal extension with Fmoc protected monomers followed by deprotection in solution or on solid-supported resin.^7–9,11,25–28^ Herein, we report alternative complementary routes towards highly functionalised compounds. These comprise (a) coupling of unfunctionalized monomers followed by subsequent introduction of the amino acid side chain mimicking group (Scheme 1c) and (b) the preparation of compounds bearing side chain functionality through late stage functionalization on the fully assembled scaffold (Scheme 1d).

**Fig. 2:**
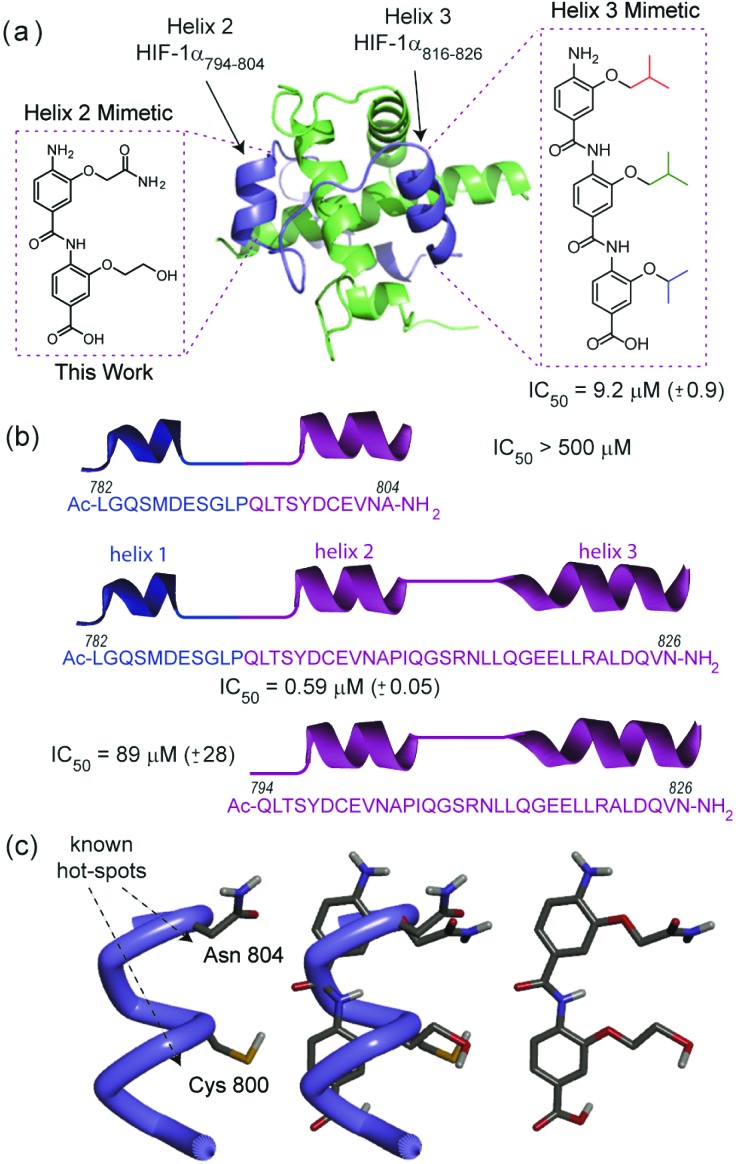
(a) Structure of the HIF-1α CTAD/p300 CH1 domain complex (PDB ID: 1L8C,^32^ p300 green, HIF-1α, blue) together with 3-*O*-alkylated aromatic oligoamide helix mimetics for helix 2 and 3 of HIF-1α designed on the basis of (b) binding studies reported previously (regions confirmed to contribute affinity shown in purple and other regions shown in blue) (c) Modelling of helix 2 mimetic (hot spot residues on helix (in blue) are highlighted).

HIF-1 is an emerging, but not yet fully validated target for cancer chemotherapy; the interaction of HIF-1α with CREB binding protein (CBP)/p300 plays a major role in regulating the hypoxic response and as such its inhibition represents an attractive approach to prevent development of new vasculature by hypoxic tumours. The HIF-1α/p300 interaction is substantially mediated by the binding of HIF-1α around the p300 CH1 domain and involves 3 regions which adopt a helical conformation on binding to p300 (Fig 2a).^29,30^ We recently reported that helix 3 plays the dominant role in mediating potency of recognition but that helix 2, either as a fusion with helix 1 or 3 plays a role in binding (Fig. 2b).^31^ We also demonstrated that helix 3 could be effectively mimicked using a 3-*O*-alkylated aromatic oligoamide helix mimetic (Fig 2c). Motivated by this, we sought to synthesise mimics of the helix-2 sequence and explore the structure activity relationship around the helix-3 mimetic. Thus, using the HIF-1α/p300 interaction as a model, the two new synthetic methods described below allow further analyses of α-helix mimicry of the p300 binding HIF-1α transactivation sequence, but equally may be applied to further studies on any of the 2 or 3-*O*-alkylated helix mimetics described to date.

## Results and discussion

### Highly functionalised compounds – helix 2

**Scheme 2:**
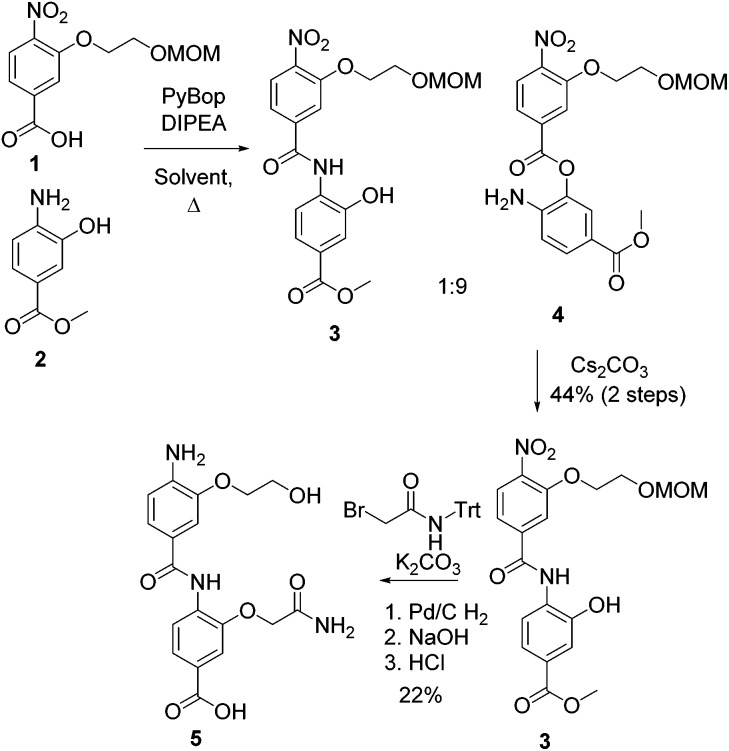
Synthesis of helix 2 mimetic by *O*–*N*-acyl shift and alkylation sequence.

Initially, we sought to prepare mimetics capable of recapitulating the side chains of a different region of the HIF-1α peptide to corroborate our observations from peptide binding studies. The helix 2 region of the HIF-1α CTAD contains two reported “hot-spot” residues which are also the sites of post-translational modifications, Cys_800_ and Asn_804_ as shown in Fig. 2.^33,34^ Initial attempts to prepare helix 2 mimics (Fig. 2c) using monomers designed to mimic Asn, Cys and Ser (see ESI, Scheme S2† for structures and synthesis) and either of our established protocols were hampered by inefficient coupling between the respective amine and acid building blocks. We tentatively suggest that the lack of coupling arises as a consequence of the extra steric bulk conferred by the large protecting groups on each monomer (see Table S1†) along with the poor nucleophilicity of *ortho*-amino phenols.^25,35^ After screening a range of amide bond forming conditions (see ESI, Table S1†), an alternative synthetic strategy was investigated building on the work of Ahn;^35^ this ligation approach exploits the efficiency of intramolecular acylation. The nitro acid monomer **1** was reacted with methyl 4-amino-3-hydroxybenzoate **2** to give a 1 : 9 mixture of the amide **3** and the ester **4**; *O*- to *N*-acyl transfer was then promoted by treating the mixture with caesium carbonate to afford the desired compound **3** in a moderate 44% yield. The second side chain was then introduced with 2-bromo-*N*-(triphenylmethyl)-acetamide before final reduction, hydrolysis and deprotection to yield the desired, densely functionalised dimer **5** as shown in Scheme 2. In the design of **5** an alcohol side chain was used on the *N*-terminal amino benzoic acid monomer *in lieu* of a thiol.^36,37^ The *O*- to *N*-acyl synthetic method was also employed to synthesise the reverse side chain orientation of **5** (see ESI†). In principle this synthetic method should be fully compatible with the solid-phase synthesis methodologies previously reported and facilitate the preparation of highly functionalised helix mimetics for use against other targets.

### Late stage derivatisation – helix 3

Having demonstrated synthetic feasibility for late stage alkylation of the phenolic position in the course of synthesising dimer **5** (see above), we further adapted this approach to permit late stage derivatisation of the trimeric oligobenzamide scaffold (Fig. 1a) and rapid library generation for structure activity relationship (SAR) studies around our initial helix 3 inhibitors.^38^ Late stage derivatisation reduces the requirement to build and assemble large sets of monomers and should allow the incorporation of delicate functionalities (*e.g.* trifluoromethyl diazirines to support proteomics analyses and binding site identification by cross-linking).

**Scheme 3:**
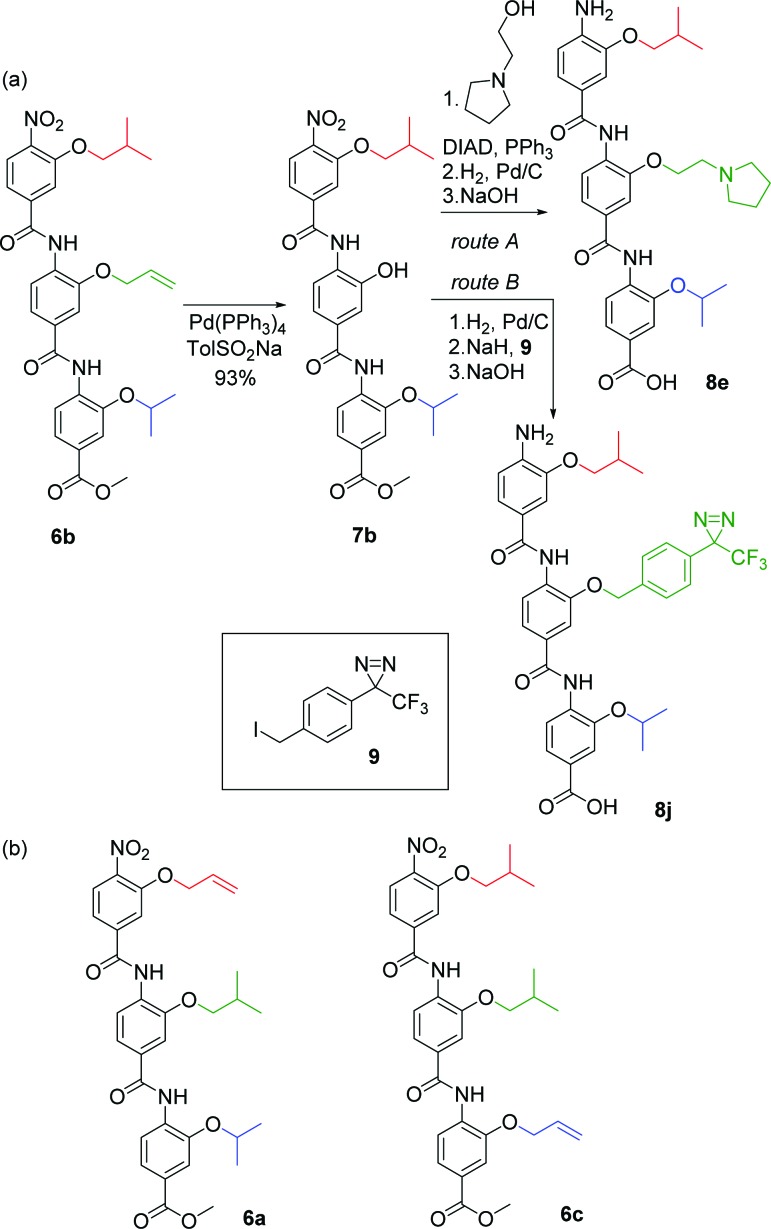
Late stage helix mimetic derivatisation (a) representative synthetic route for **6b** (b) structures of compounds **6a** and **6b**.

Using the recently identified proteomimetic inhibitor (see Fig. 2a) of the HIF-1α/p300 interaction as inspiration,^10^ a series of compounds with cleavable protecting groups was prepared, *e.g.* **6b**. Phenolic allyl ethers are readily cleaved *via* the formation of a π-allyl complex with palladium(0) but are relatively inert to other conditions. This makes then ideal for use in the late stage derivatisation of oligobenzamides. Indeed, the allyl groups in **6a–c** were readily cleaved with palladium(0) tetrakis(triphenylphosphine) employing toluene sulfinic acid as a scavenger (Scheme 3 for a representative example (**6b** → **7b**)).

The phenols **7a–c** could then be functionalised by employing *Mitsunobu* chemistry or alkylation with an appropriate halide yielding functionalized helix mimetics which, upon reduction of the aryl nitro group and ester hydrolysis in one pot, afford final compounds **8a–i** (Table 1), following purification (Scheme 3 for a representative example (**7b** → **8e**)). This approach was thus employed to prepare a small library of compounds **8a–i** with previously unreported side chain combinations. Alcohols and alkyl halides were selected to challenge the synthetic approach (focusing on tertiary amines) rather than a specific intention to develop optimised inhibitors.

**Table 1:** Compounds targeted to HIF-1α helix 3 binding site on p300 prepared in this study. R groups refer to Fig. 1 mimetic **A**

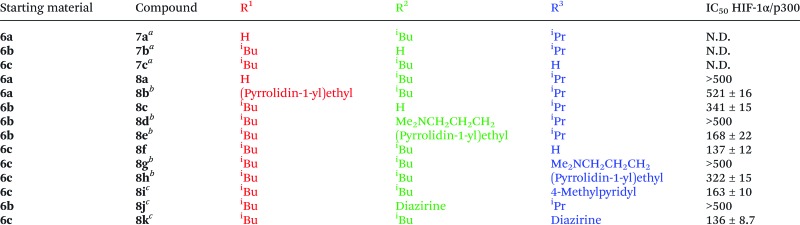

^*a*^Nitro ester compounds obtained from **6a–c**; (PPh_3_)_4_Pd, TolSO_2_Na.

^*b*^Following Route A (Scheme 3); 1. DIAD, PPh_3_, ROH, 2. H_2_, Pd(C), 3. NaOH.

^*c*^Following Route B (Scheme 3); 1. H_2_, Pd(C), 2. NaH, alkyl halide, 3. NaOH.

As a further illustrative example of the power of the approach, we incorporated a trifluoromethyl diazirine functionality into the oligobenzamide for photo-crosslinking experiments. The fragile diazirine would not be stable to the reductive conditions required to reduce the nitro groups. However, using the late stage methodology described above, the aryl trifluoromethyl diazirine was incorporated following a slightly modified sequence following all the required reductions. By selectively, and irreversibly, deprotonating the phenol **7b** with sodium hydride then treating with the iodide **9**, the desired functionality could be introduced regioselectively. Final ester hydrolysis was performed *in situ* by treating the reaction mixture with sodium hydroxide to yield the photo-crosslinking helix mimetic **8j**. **8k** was synthesized using a similar sequence of steps from compound **6c** (see ESI† for details) (Table 1).

### Biophysical analyses

Using a previously described fluorescence anisotropy competitive HIF-1α/p300 assay,^10,31^ the helix 2 mimetic **5** was shown to be ineffective as a HIF-1α/p300 inhibitor; this is surprising given that the group of Arora had previously shown a hydrogen-bond surrogate constrained peptide derived from helix 2 of HIF-1α was a good HIF-1α/p300 inhibitor^39^ but is consistent with our observations that the helix 2 peptide alone is not a useful inhibitor.^31^ The compounds prepared by late stage derivatisation (**7** and **8**) were also screened for activity (Table 1); they showed reduced activity compared to the previously reported inhibitors by us and others.^10,40^ This is consistent with the hydrophobicity of the binding groove. The diazarine bearing compound **8k** also exhibits reduced binding whereas **8j**, was not active in our assay.

In pursuit of preliminary cross-linking studies, compound **8k** was incubated with p300 and irradiated with 365 nM light followed by LC-MS analysis. LC-MS analysis revealed almost complete conversion of the diazarine moiety to carbene quenching products but unfortunately no protein labelling was observed (see Fig. S3†). The quenched carbene products and absence of cross-linking to protein suggest that the trifluoromethyl diazarine is exposed to solvent when compound **8k** is bound to p300 rather than buried in the protein surface. Further experiments with other proteins are ongoing.

## Conclusions

In summary, we have described methodology that provides access to challenging aromatic oligoamide sequences and complements current approaches for the synthesis of these widely used helix mimetics. This approach may be useful in building up a picture of structure activity relationships (SAR) and is complementary to solution and SPS strategies for helix mimetic assembly useful for library production and initial screening.^8,18,25^ To appreciate the power of this alternative synthetic approach, compounds **8b,d–e,g–i** were prepared in a total of 39 synthetic steps whereas by contrast, it would have required 51 synthetic steps to prepare this library using our previously described methods.^10,14,25^ We have exemplified this with our preliminary results for inhibition of a key target in tumour metabolism, HIF-1α/p300.
