# Supplementary material for: Synthesis of highly functionalized oligobenzamide proteomimetic foldamers by late stage introduction of sensitive groups
Source: Org Biomol Chem. 2016 Mar 23;14(15):3782–6. doi: 10.1039/c6ob00078a (PMC4839272; doi:10.1039/c6ob00078a)
Supplement: Supplementary file 1 [file OB-014-C6OB00078A-s001.pdf]

Electronic Supplementary Material (ESI) for Organic & Biomolecular Chemistry.
This journal is © The Royal Society of Chemistry 2016

Routes to Highly Functionalised Oligobenzamide Proteomimetics

George M. Burslem,[a, b] Hannah F. Kyle,[b,c] Panchami Prabhakaran, Alex Breeze,[b,c,d] Thomas A. Edwards,[b, c] Stuart L. Warriner,[a, b] Adam Nelson[a, b] and Andrew J. Wilson*[a, b]

Supporting Information

[a] School of Chemistry, University of Leeds, Woodhouse Lane, Leeds LS29JT, UK, [b] Astbury Centre for Structural Molecular Biology, University of Leeds, Woodhouse Lane, Leeds LS29JT, UK, [c] School of Molecular and Cellular Biology, Faculty of Biological Sciences, University of Leeds, Woodhouse Lane, Leeds LS2 9JT, UK, [d] Discovery-Enabling Capabilities and Sciences, AstraZeneca R&D, Alderley Park, Cheshire SK10 4TG, UK.

General Experimental Considerations 2
Oligobenzamide Nomenclature 2
Supplementary Figures and Schemes 3
Synthesis 5
- Standard Procedures 5
- Highly Functionalised Trimers 6
- Late Stage Derivatisaion 14

Competition Assays 25
Photo-Crosslinking Experiments 26
NMR and HPLC Spectra for final compounds 28
- References 46

General Experimental Considerations

All commercial solvents and reagents were used without further purification unless stated otherwise. All non-aqueous reactions were performed under an atmosphere of nitrogen and using anhydrous solvents. Water-sensitive reactions were performed in oven-dried glassware, cooled under nitrogen before use, or flame dried and cooled, under vacuum if stated. Solvents were removed under reduced pressure using a Büchi rotary evaporator. Ether refers to diethyl ether and petrol refers to petroleum spirit (b.p. 40-60 °C). Flash column chromatography was carried out using silica (35-70 μm particles) or alumina (neutral, Brockman activity 1), with crude reaction mixtures loaded in the initial solvent system or its least polar constituent. Thin layer chromatography was carried out on commercially available silica pre-coated aluminium plates (Kieselgel 60 F254, Merck) or commercially available alumina pre-coated glass plates (neutral, Brockman activity 1). Strong cation exchange columns were carried out using SCX, 5.0 g pre-packed cartridge, Supelco.

Proton and carbon NMR spectra were recorded on a Bruker Avance 500, Avance DPX300 or DRX500 spectrophotometer with an internal deuterium lock. Carbon NMR spectra were recorded with composite pulse decoupling using the waltz 16 pulse sequence. Chemical shifts are quoted in parts per million downfield of tetramethylsilane, and coupling constants (*J*) are given in Hz. NMR spectra were recorded at 300 K unless otherwise stated. Infra-red spectra were recorded using a Perkin–Elmer Spectrum One FT-IR spectrophotometer. Melting points were determined using a Griffin and George melting point apparatus and are uncorrected. Nominal mass spectrometry was routinely performed on a Bruker HCT Ultra spectrometer using electrospray (+) ionization. Nominal and accurate mass spectrometry using electrospray ionisation was carried out by staff or the candidate in the School of Chemistry using a Micromass LCT-KA111, Bruker MicroTOF or Bruker MaXis Impact TOF mass spectrometer. Mass-directed HPLC purifications were run on an Agilent 1260 Infinity Preparative HPLC system equipped with a Waters XBridge™ Prep C18 19 × 100 mm column, 5 μm particle size, on an acetonitrile or methanol/water gradient (5-95% acetonitrile or methanol over 8 minutes) and an Agilent 6120 Quadrupole system equipped with a quadrupole MS detector, using electrospray ionisation (ESI).

Oligobenzamide Nomenclature

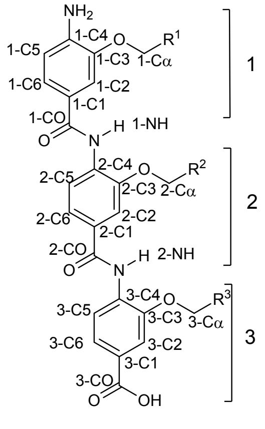

To simplify the numbering and NMR assignment of oligobenzamides, we have devised a sequential nomenclature, where each of the monomer building blocks is considered separately. The monomers are numbered from 1 to 3 starting from the *N*-terminal. Within each monomer, the numbering is the same: the carbons from the aminobenzoic acid are numbered using the standard system (the aromatic carbon bearing the carboxylic acid is C1, the one bearing the amine is C4). Then, the lateral chain is numbered: the carbon attached to the oxygen is the Cα, and the numbering of the aliphatic part of the side chain continues with Cβ, etc. In the case of aromatic side chains,

the aromatic carbons are numbered CAr1, CAr2, etc. The numbering of the protons is based on the carbon numbering. To differentiate each individual carbon/proton, the monomer number is added as a prefix to the carbon/proton number representative examples are given above.

Supplementary Figures and Schemes

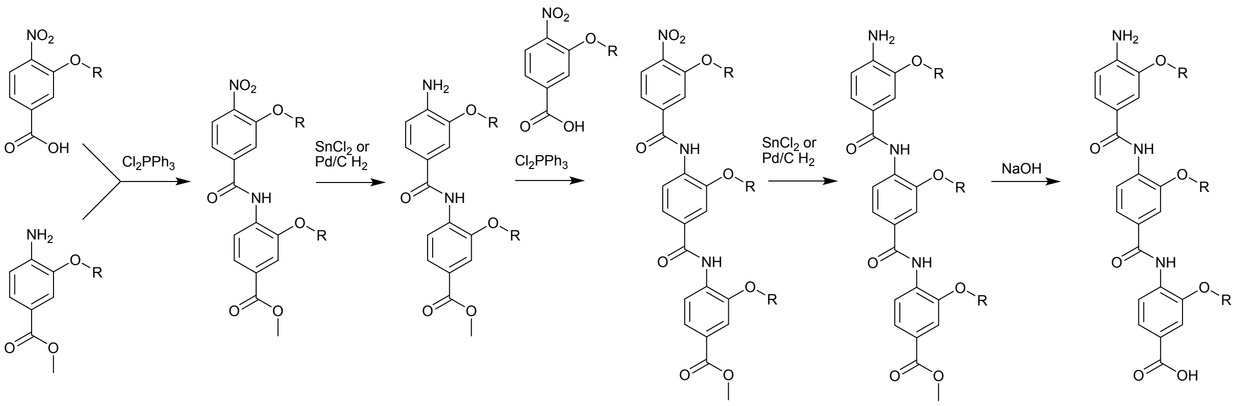

Scheme S1. General Synthesis of Oligobenzamides

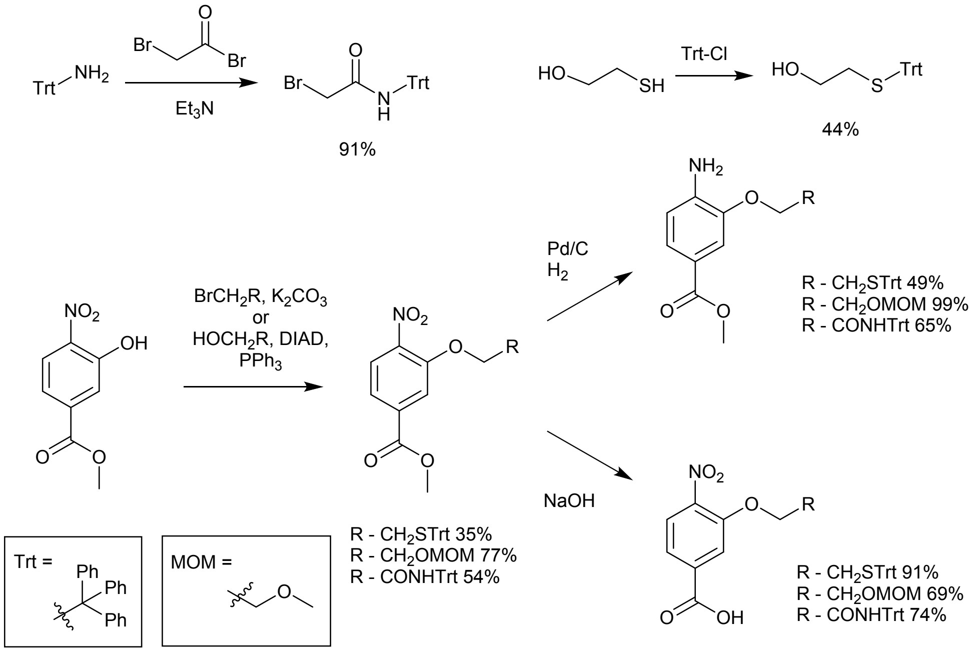

Scheme S2. Synthesis of Building Blocks

Table S1 - Attempted Coupling Conditions

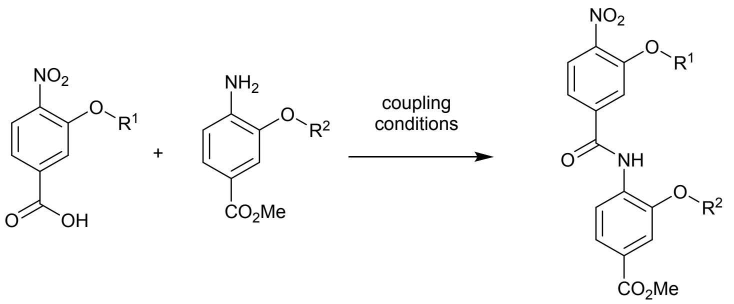

Entry	R^1	R^2	Conditions	Conversion by crude NMR
**1**	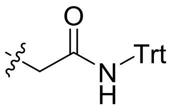	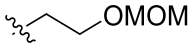	HATU, DIPEA	No conversion
**2**	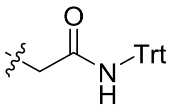	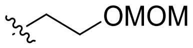	$SOCl_2$	No conversion
**3**	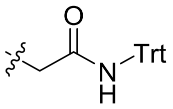	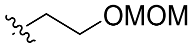	Ghosez's Reagent	~5%
**4**	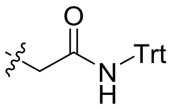	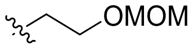	Cl_2PPh_3	No conversion
**5**	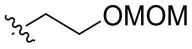	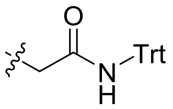	Ghosez's Reagent	~5%
**6**	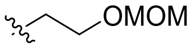	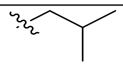	Cl_2PPh_3	Complete conversion
**7**	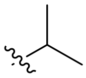	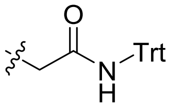	Cl_2PPh_3	Complete conversion

Synthesis

Standard Procedures

Standard Procedure A - Ester Hydrolysis

An aqueous sodium hydroxide solution (2 M, 1 ml per 100 mg of ester) was added to a solution of the ester in methanol (~5 ml per 100 mg of ester) and stirred at room temperature until the starting material had been consumed, as observed by TLC. The reaction mixture was concentrated by half *in vacuo* then adjusted to pH 3 by the addition of 1M HCl (aq.); the resulting precipitate was isolated by filtration, dried *in vacuo* and used without further purification in subsequent steps.

Standard Procedure B – Tin mediated nitro reduction

Tin (II) chloride dihydrate (5 equivalents) was added in one portion to a solution of the nitro compound in ethyl acetate (5 ml per 100 mg) and the reaction stirred at 50 °C under a calcium chloride drying tube for 24 hours. The reaction was then allowed to cool to room temperature and poured into 2 M sodium hydroxide solution (5 ml per 100 mg of starting material). The organic layer was separated, washed with 2 M sodium hydroxide solution (2 × 5 ml per 100 mg of starting material) and brine (5 ml per 100 mg of starting material), dried over $MgSO_4$ and concentrated *in vacuo*. The residue was purified by flash column chromatography to give the desired compound.

Standard Procedure C – Nitro reduction by hydrogenation

Palladium on charcoal (10 %) was added against a flow of nitrogen to a solution of the nitro compound in methanol (10 ml per 100 mg) under a nitrogen atmosphere, the atmosphere was then replaced with hydrogen and the reaction stirred vigorously until complete by TLC (typically 2 hours). The hydrogen atmosphere was vented and the reaction mixture filtered through a pad of Celite with methanol, concentrated *in vacuo* and purified by flash column chromatography.

Standard Procedure D - Coupling

Dichlorotriphenylphosphorane (4.5 equivalents) was added to a solution of nitro-acid compound (1.2 equivalents) in chloroform (5 ml per 100 mg of amine) and the reaction heated to reflux with stirring under nitrogen. After 2 hours at reflux, the amine ester compound (1 equivalent) was added as solution in chloroform (1 ml) and the reaction was heated to reflux for a further 24 hours. The reaction mixture was then concentrated *in vacuo* and partitioned between ethyl acetate (5 ml per 100 mg of amine) and H_2O (5 ml per 100 mg of amine). The organic layer was separated and washed with saturated aqueous sodium bicarbonate solution (5 ml per 100 mg of amine), dried over magnesium sulphate and

concentrated *in vacuo*. The resulting residue was purified by flash column chromatography to give the desired compound.

Standard Proceedure E – Removal of Allyl Groups

Allyl functionalised trimers were treated with palladium tetrakistriphenylphosphine (10 mol%) and sodium toluenesulfinate (1.2 eq) in THF (1ml/mg) overnight, concentrated *in vacuo* and purified by column chromatography eluting with Et_2O in DCM.

Standard Procedure F – Side chain introduction by Mitsunobu reaction

De-allylated nitro ester trimers were dissolved in THF (1 ml/10 ml substrate) and PPh_3 (2 eq.), alcohol (2 eq.) and DIAD (2 eq.) added sequentially. The reaction was stirred overnight at r.t., concentrated *in vacuo*. Amine products were isolated by SCX column and used without further purification.

Highly Functionalised Trimers

2-Bromo-*N*-(triphenylmethyl)-acetamide, S1

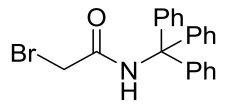

A solution of tritylamine (500 mg, 1.93 mmol) and triethylamine (0.73 mL, 5.25 mmol) in CH_2Cl_2 (5ml) was added drop-wise to a cooled solution (0 °C) of bromo-acetyl bromide (0.8 mL, 1.75 mmol) and triethylamine (0.73 mL, 5.25 mmol) in DCM (10 mL) with rapid stirring under an inert atmosphere. The reaction was stirred and allowed to warm to room temperature over 15 hours. The reaction mixture was concentrated *in vacuo* and immediately purified by column chromatography eluting with CH_2Cl_2. Fractions containing product were concentrated *in vacuo* to a black solid which upon trituration with MeOH gave the product as a colourless solid (608 mg, 91%). m.p. 201-203 °C (CH_2Cl_2); R_f 0.35 (CH_2Cl_2); ν_{max}/cm^{-1} (solid state) 3261, 3053, 3032, 1660; δ_H (500 MHz; $CDCl_3$) 7.75 (1H, s, NH), 7.33 (10H, m, Ar-H), 7.25 (5H, d, J = 6.9 Hz, Ar-H), 3.91 (2H, s, CH_2); δ_C (125 MHz; $CDCl_3$) 164.3 (Carbonyl), 144.1 (trityl), 128.6 (trityl), 128.2 (trityl), 127.3 (trityl), 30.0 (CH_2) quaternary carbon not observed; *m/z* (ES) 404 (100%, MH^+), 402 (100%, MH^+), 243 (50%, Trt^+); HRMS Found: 402.0475; $C_{21}H_{18}NOBr$ $[M+Na]^+$ requires 402.0464.

***S*-trityl-mercapto-ethanol, S2**

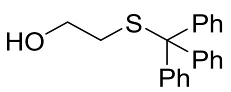

Following a literature procedure,[1] mercaptoethanol (0.23 ml, 3.27 mmol) was added to a solution of trityl chloride (1g, 3.59 mmol) in THF (5 mL) and heated to reflux for 4 hours, the reaction was allowed to cool, concentrated *in vacuo* and triturated with 1:2 EtOAc–Hexane to yield the desired compound as a colourless solid (466 mg, 44%). m.p. 108-110 °C (Hexane); ν_{max}/cm^{-1} (film) 3336 (broad), 3063, 2926, 1592; δ_H (500 MHz; $CDCl_3$) 7.50 (6H, d, J = 7.8 Hz, trityl), 7.34 (6H,

m, trityl), 7.27 (3H, m, trityl), 3.42 (2H, t, J = 6.2 Hz, CH_2), 2.53 (2H, t, J= 6.2 Hz, CH_2), 1.93 (1H, s, OH); δ_C (125 MHz; $CDCl_3$) 144.8 (trityl), 129.7 (trityl), 128.1 (trityl), 126.8 (trityl), 66.74 (CH_2S), 35.26 (CH_2O), quaternary carbon not observed; *m/z* (ES) $[MH]^+$ 343, $[2M+Na]^+$ 663; HRMS Found: 343.1143; $C_{21}H_{20}OS$ $[M+Na]^+$ requires 343.1127

Methyl 4-nitro-3-{2-[(triphenylmethyl)sulfanyl]ethoxy}benzoate, S3

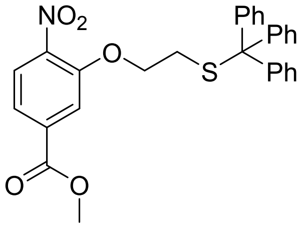

Diisopropyl azodicarboxylate (DIAD) (0.09 mL, 0.45 mmol) was added to a cooled solution (0 °C) of methyl 3-hydroxy-4-nitrobenzoate (88 mg, 0.45 mmol), *S*-trityl mercaptoethanol (200 mg, 0.63 mmol) and triphenylphosphine (116 mg, 0.45 mmol) in anhydrous THF (5 mL) under an inert atmosphere and reaction allowed to warm to room temperature with stirring. After 3 days the reaction was concentrated *in vacuo*, dissolved in CH_2Cl_2 (10 mL) and washed with H_2O (2 × 10 mL), saturated aqueous sodium bicarbonate (3 × 10 mL) and brine (10 mL), dried over $MgSO_4$ and concentrated *in vacuo* to a yellow oil. The oil was purified by column chromatography eluting with 1:1 CH_2Cl_2–petrol and 7:3 CH_2Cl_2–petrol sequentially to give the product as a colourless oil which crystallized to colourless needles on standing (79 mg, 35%). m.p. 79-81 °C (7:3 CH_2Cl_2, Petrol); R_f 0.35 (1:1, CH_2Cl_2, Petrol); ν_{max}/cm^{-1} (Solid State) 3054, 2478, 2254, 2159, 2028, 1974, 1727; δ_H (500 MHz; $CDCl_3$) 7.70 (1H, d, J = 8.2 Hz, Ar-H), 7.57 (1H, dd, J = 8.5, 1.6 Hz, Ar-H), 7.38 (7H, d, J = 7.8 Hz, trityl and Ar-H), 7.22 (6H, t, J = 7.8 Hz, trityl), 7.13 (3H, t, J = 7.8 Hz, trityl), 3.87 (3H, s, CO_2Me), 3.64 (2H, t, J = 6.8 Hz, CH_2S), 2.63 (2H, t, J = 6.8 Hz, OCH_2); δ_C (125 MHz; $CDCl_3$) 165.1, 151.4, 144.5, 142.5, 134.7, 129.6, 128.1, 126.9, 125.3, 121.6, 115.7, 68.3, 67.0, 52.8, 33.7; HRMS Found: 522.1339; $C_{29}H_{25}NO_5S$ $[M+Na]^+$ requires 522.1346.

Methyl 3-[2-(methoxymethoxy)ethoxy]-4-nitrobenzoate, S4

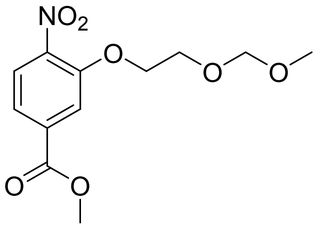

1-Bromo-2-(methoxymethoxy)ethane (0.41 mL, 3.55 mmol) was added to a solution of methyl 3-hydroxy-4-nitrobenzoate (500 mg, 2.54 mmol) and potassium carbonate (1.7 g, 12.7 mmol) in DMF (10 mL) and heated to 50 °C for 24 hours, allowed to cool and partitioned between EtOAc (20 mL) and H_2O (20 mL). The organic layer was separated and washed with H_2O (20 mL) and brine (20 mL), dried over $MgSO_4$, concentrated *in vacuo* to give a yellow solid and purified by column chromatography eluting with CH_2Cl_2 to give the desired product as a pale yellow solid (209 mg, 29%). m.p. 58-60 °C (CH_2Cl_2); R_f 0.3 (CH_2Cl_2); ν_{max}/cm^{-1} (Solid state) 2966, 2942, 2891, 1726; δ_H (500 MHz; $CDCl_3$) 7.83 (1H, d, J = 8.2 Hz, Ar-H), 7.77 (1H, s, Ar-H), 7.69 (1H, d, J = 8.2 Hz, Ar-H), 4.70 (2H, s, CH_2), 4.35 (2H, t, J = 4.6 Hz, CH_2), 3.96 (3H, s, CO_2Me), 3.94 (2H, t, J = 4.6 Hz, CH_2), 3.38 (3H, s, OMe); δ_C (125 MHz;

CDCl3) 165.1, 151.7, 142.6, 134.8, 125.3, 121.6, 115.7, 96.6, 69.4, 65.3, 55.2, 52.8; HRMS Found: 308.0746; $C_{12}H_{15}NO_7$ $[M+Na]^+$ requires 308.0741.

Methyl 3-[(triphenylmethyl)carbamoyl]methoxy-4-nitrobenzoate, S5

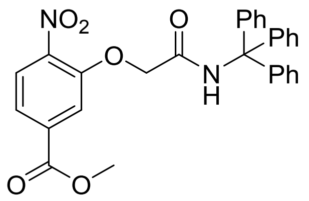

2-Bromo-*N*-(triphenylmethyl)-acetamide (600 mg, 1.58 mmol) was added to a solution of methyl 3-h ydroxy-4-nitrobenzoate (258mg, 1.31 mmol) and potassium carbonate (900 mg, 6.58 mmol) in DMF (10 mL) and heated to 50 °C for 24 hours then allowed to cool and partitioned between EtOAc (20 mL) and H_2O (20 mL). The organic layer was separated and washed with saturated aqueous solution of sodium bicarbonate (20ml), H_2O (20 ml) and brine (20 ml), dried over $MgSO_4$, concentrated *in vacuo* to an orange solid which was recrystallised from methanol to give a white solid (155.4 mg, 23%), m.p. 205 °C (MeOH), ν_{max}/cm^{-1} (film) 3407, 3058, 2950, 2512, 2159, 1976, 1728; δH (500 MHz; $CDCl_3$) 8.14 (1H, s (broad), N-H), 8.02 (1H, d, *J* = 8.1 Hz, Ar-H), 7.80 (1H, dd, *J* = 8.5 Hz, 1.7, Ar-H), 7.74 (1H, d, *J* = 1.4 Hz, Ar-H), 7.34-7.26 (15H, m, trityl), 4.68 (2H, s (broad), CH_2), 3.98 (3H, s (broad), OMe); δC (125 MHz; $CDCl_3$) 164.9, 164.6, 150.2, 144.2, 135.8, 128.6, 128.0, 127.2, 126.4, 122.8, 155.5, 91.9, 70.6, 68.3, 53.0; HRMS Found: 519.1533; $C_{29}H_{24}N_2O_6$ $[M+Na]^+$ requires 519.1527.

Methyl 3-{2-[(triphenylmethyl)sulfanyl]ethoxy}-4-aminobenzoate, S6

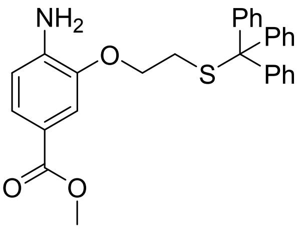

Prepared using procedure C from methyl 3-{2-[(triphenylmethyl)sulfanyl]ethoxy}-4-nitrobenzoate (220 mg, 0.44 mmol) and purified by alumina column chromatography eluting with 1% ethyl acetate–DCM followed by 1% MeOH–DCM to give a pale yellow oil (101 mg, 49%), ν_{max}/cm^{-1} (film) 3486, 3374, 3057, 2948, 1704, 1615; δH (300 MHz; $CDCl_3$) 7.52 (1H, dd, *J* = 8.2, 1.6 Hz, Ar-H), 7.45 (6H, m, Trityl and Ar-H), 7.25 (10H, m, Trityl), 6.63 (1H, d, *J* = 8.2 Hz, Ar-H), 3.83 (5H, m, OMe and CH_2), 2.65 (2H, t, J = 6.3 Hz, CH_2); δC (75 MHz; $CDCl_3$) 144.8, 144.6, 141.4, 129.5, 128.0, 126.8, 124.4, 119.3, 113.2, 112.6, 66.99, 51.72, 31.54; HRMS Found: 492.1603; $C_{29}H_{27}NO_3S$ $[M+Na]^+$ requires 492.1604.

Methyl 3-[2-(methoxymethoxy)ethoxy]-4-aminobenzoate, S7

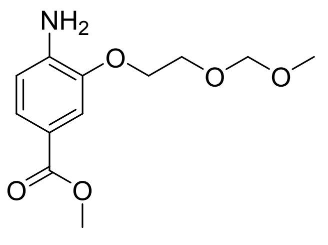

Prepared using procedure C from methyl 3-[2-(methoxymethoxy)ethoxy]-4-nitrobenzoate (416 mg, 1.46 mmol) to give a pale yellow oil (369 mg, 99%), ν_{max}/cm^{-1} (film) 3480, 3365, 2949, 2887, 1704; δ_H (500 MHz; $CDCl_3$) 7.56 (1H, d, *J* = 8.2 Hz, Ar-H), 7.48 (1H, s, Ar-H), 6.67 (1H, d, *J* = 7.8 Hz, Ar-H), 4.71 (2H, s, OCH_2O), 4.38 (2H, s (broad), NH_2), 4.22 (2H, t, *J* = 4.4 Hz, CH_2), 3.92 (2H, t, *J* = 4.4 Hz, CH_2), 3.86 (3H, s, OMe), 3.39 (3H, s, OMe); δ_C (125

MHz; $CDCl_3$) 167.2, 145.0, 141.8, 124.5, 119.2, 113.3, 113.0, 96.5, 68.1, 66.1, 55.3, 51.6; HRMS Found: 278.0999; $C_{12}H_{17}NO_5$ $[M+Na]^+$ requires 278.0999.

Methyl 3-[(triphenylmethyl)carbamoyl]methoxy-4-aminobenzoate, S8

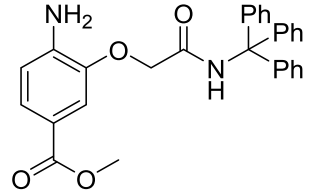

Prepared using procedure C from methyl 3-[(triphenylmethyl)carbamoyl]methoxy-4-nitrobenzoate (220 mg, 0.44 mmol) to give a beige solid (153 mg, 65%), m.p. 132-134 °C, ν_{max}/cm^{-1} (film) 3338, 3056, 2949, 2581, 1966, 1671; δ_H (500 MHz; d_6-DMSO) 8.76 (1H, s, NH), 7.42 (1H, dd, J = 8.2, 1.8 Hz, Ar-H), 7.36 (1H, d, J = 1.4 Hz, Ar-H), 7.28-7.17 (15H, m, trityl), 6.68 (1H, d, J = 8.2 Hz, Ar-H), 5.72 (2H, s (broad), NH_2), 4.75 (2H, s, CH_2), 3.79 (3H, s, OMe); δ_C (125 MHz; d_6-DMSO) 167.1, 166.2, 144.4, 143.8, 128.4, 127.7, 127.5, 126.5, 124.3, 112.9, 112.4, 69.2, 61.8, 51.3, 48.5; HRMS Found: 489.1790, $C_{29}H_{26}N_2O_4$ $[M+Na]^+$ requires 489.1785.

4-Nitro-3-{2-[(triphenylmethyl)sulfanyl]ethoxy}benzoic acid, S9

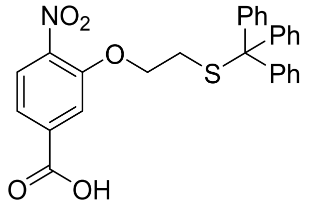

Prepared using procedure A from methyl 4-Nitro-3-{2 [(triphenylmethyl)sulfanyl]ethoxy} benzoate (220 mg, 0.44 mmol) to give a colourless solid (195 mg, 91%), m.p. 194-196 °C ν_{max}/cm^{-1} (film) 3057, 2654, 1693; δ_H (500 MHz; $CDCl_3$) 7.94 (1H, d, J = 8.2 Hz, Ar-H), 7.65 (1H, dd, J = 8.2 Hz, 1.4, Ar-H), 7.56 (1H, s, Ar-H), 7.37-7.24 (15H, m, trityl), 3.99 (2H, t, J = 6.2 Hz, CH_2), 2.54 (2H, t, J = 6.2 Hz, CH_2); δ_C (125 MHz; $CDCl_3$) 165.6, 150.1, 144.1, 142.1, 129.0, 128.0, 126.8, 124.9, 121.6, 115.4, 67.56, 66.19, 30.64; HRMS Found: 508.119; $C_{28}H_{23}NO_5S$ $[M+Na]^+$ requires 508.1189.

3-[2-(Methoxymethoxy)ethoxy]-4-nitrobenzoic acid, 1

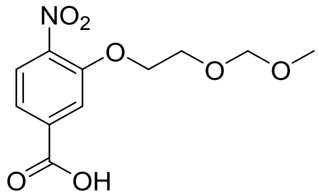

Prepared using procedure A from methyl 3-[2-(methoxymethoxy)ethoxy]-4-nitrobenzoate (400 mg, 1.4 mmol) to give a colourless solid, (262.6 mg, 69%), m.p. 131-133 °C; ν_{max}/cm^{-1} (film) 3063, 2938, 2542, 2159, 2025, 1691; δ_H (500 MHz; $CDCl_3$) 7.90-7.87 (2H, m, Ar-H), 7.81 (1H, dd, J = 8.2, 1.4 Hz, Ar-H), 4.76 (2H, s, OCH_2O), 4.41 (2H, t, J = 4.4 Hz, CH_2), 4.00 (2H, t, J = 4.4 Hz, CH_2), 3.44 (3H, s, OMe); δ_C (125 MHz; $CDCl_3$) 169.4, 151.7, 143.3, 133.8, 125.4, 122.4, 116.3, 96.6, 69.5, 65.4, 55.3; HRMS Found: 294.0571, $C_{11}H_{13}NO_7$ $[M+Na]^+$ requires 294.0584;

3-[(Triphenylmethyl)carbamoyl]methoxy-4-nitrobenzoic acid, S10

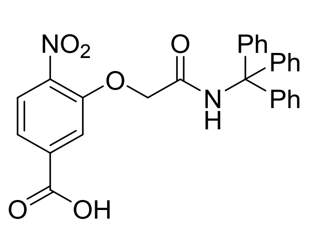

Prepared using procedure A from methyl 3-[(triphenylmethyl)carbamoyl]methoxy-4-nitrobenzoate (220mg, 0.44 mmol) to give an off white solid (157mg, 74%); m.p. 222-224 °C; ν_{max}/cm^{-1} (film) 3398, 3059, 2828, 2581, 1961, 1699; δ_H

(500 MHz; d_6-Acetone) 8.21 (1H, s (broad), NH), 8.07 (1H, d, J = 8.2 Hz, Ar-H), 7.93 (1H, d, J = 1.4 Hz, Ar-H), 7.84 (1H, dd, J = 8.5, 1.6 Hz, Ar-H), 7.32-7.25 (15H, m, trityl), 4.98 (2H, s, CH_2); δ_C (125 MHz; CDCl3) 166.1, 165.7, 151.4, 145.7, 143.1, 136.5, 129.6, 128.5, 127.7, 126.4, 123.3, 117.0, 70.92, 69.22, 49.6; HRMS Found: 505.1362; $C_{28}H_{22}N_2O_6$ $[M+Na]^+$ requires 505.137.

Methy 3-Hydroxy-4-aminobenzoate, 2

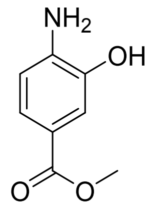

4-amino-3-hydroxybenzoic acid (1 g, 6 mmol) was heated to reflux in methanol (20 ml) and sulphuric acid (2 ml) for 16 hours, allowed to cool to r.t. and sodium bicarbonate added until gas evolution ceased. The resulting solid was removed by filtration and the filtrate concentrated *in vacuo*, suspended in water (20 ml) and extracted with ethyl acetate (3 × 30 ml). The combined organics were washed with brine (20 ml), dried over $MgSO_4$ and concentrated *in vacuo* to yield the *title compound* as a beige solid (854 mg, 85%). ν_{max}/cm^{-1} (solid state) 3398, 3315, 2948, 2583, 1705, 1604; δ_H (500 MHz; d_6-DMSO) 9.40 (1H, s, OH), 7.26 (2H, m, Ar-H), 6.60 (1H, d, J = 8.7 Hz, Ar-H), 5.36 (2H, s, NH), 3.74 (3H, s, OMe); δ_C (125 MHz; d_6-DMSO) 166.4, 142.7, 142.3, 122.5, 116.5, 116.2, 114.5, 112.4, 51.1; HRMS *m/z* (ESI) Found: 190.0493, $C_8H_8NO_3$ $[M+Na]^+$ requires 190.0475.

O_2N-[*O*-MOM-Hydroxyethyl(3-HABA)]-[(3-HABA)]-COOMe, 3

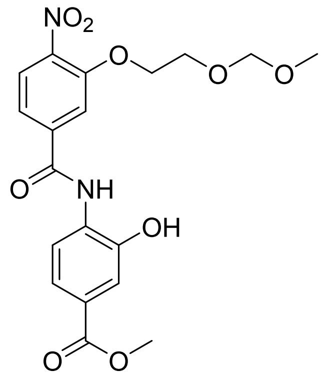

PyBOP (1664 mg, 3.2 mmol) and diisopropylethylamine (1.2 ml, 6.67 mmol) were added sequentially to a solution of 3-[2-(Methoxymethoxy)ethoxy]-4-nitrobenzoic acid (725 mg, 2.67 mmol) in dichloromethane (30 ml) and the reaction stirred for 30 minutes. After which time methy 3-hydroxy-4-aminobenzoate (446 mg, 2.67 mmol) was added and the reaction stirred at r.t. overnight. The reaction mixture was concentrated *in vacuo* and re-dissolved in dimethylformamide (20 ml). Caesium carbonate (3.8 g, 13.55 mmol) was added and the reaction stirred at r.t. for overnight. The reaction mixture was then poured into ethyl acetate (40 ml) and washed copiously with water and brine, dried over $MgSO_4$ and concentrated *in vacuo*. The residue was purified by column chromatography eluting with 5:95 ether/dichloromethane to give the *title compound* as a yellow solid (491 mg, 44%).. ν_{max}/cm^{-1} (solid state) 2952, 2869, 1716, 1674, 1524; ^1H NMR (500 MHz, d_6-DMSO) δ 10.39 (s, 1H, OH), 9.84 (s, 1H, Amide NH), 8.02 (d, J = 8.3 Hz, 1H, Ar-H), 7.92 (d, J = 8.3 Hz, 1H, Ar-H), 7.89 (s, 1H, Ar-H), 7.66 (dd, J = 8.3, 1.4 Hz, 1H), 7.55 (d, J = 1.9 Hz, 1H, Ar-H), 7.50 (dd, J = 8.3, 1.9 Hz, 1H, Ar-H), 4.64 (s, 2H, O-CH_2-O), 4.51 – 4.39 (t, 2H, CH_2), 3.92 – 3.80 (m, 5H, OMe and CH_2), 3.28 (s, 3H, OMe); HRMS found 421.1245, $C_{19}H_{20}N_2O_9$ $[M+H]^+$ requires 421.1241

O_2N-[*O*-MOM-hydroxyethyl(3-HABA)]-[*O*-(N-Trt)carbamoylmethoxy-(3-HABA)]-COOMe, S11

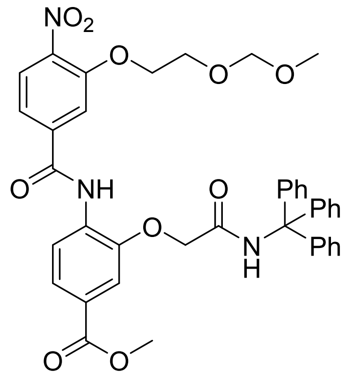

2-Bromo-*N*-(triphenylmethyl)-acetamide (217 mg, 0.57 mmol) was added to a suspension of methyl 3-hydroxy-4-{3-[2-(methoxymethoxy)ethoxy]-4-nitrobenzamido}benzoate (200 mg, 0.48 mmol) and potassium carbonate (331 mg, 2.4 mmol) in dimethylformamide (20 ml) and the reaction heated to 50 °C overnight. The reaction mixture was then poured into water (20 ml) and the resulting precipitate collected by filtration. The precipitate was purified by column chromatography eluting with 5:95 ether/dichloromethane to give the *title compound* as a pale yellow solid (138 mg, 40%). ν_{max}/cm^{-1} (solid state) 3381, 2964, 2821, 1969; ^{1}H NMR (500 MHz, $CDCl_3$) δ 9.49 (s, 1H, Amide N-H), 8.53 (d, *J* = 8.5 Hz, 1H, Ar-H), 7.86 (d, *J* = 8.5 Hz, 1H, Ar-H), 7.70 (d, *J* = 8.8 Hz, 1H, Ar-H), 7.63 (s, 1H, Ar-H), 7.44 (d, *J* = 8.4 Hz, 1H, Ar-H), 7.27 (m, *J* = 11.9, 6.7 Hz, 10H, Trityl), 7.20 (d, *J* = 7.1 Hz, 1H, Ar-H), 7.14 – 7.08 (m, 5H, Trityl), 4.68 (s, 4H, O-CH_2-O and O-CH_2-CONR), 4.21 – 4.13 (t, *J* = 4.2 Hz 2H, CH_2), 3.92 (s, 3H, OMe), 3.76 – 3.71 (t, *J* = 4.2 Hz, 2H, CH_2), 3.40 (s, 3H, OMe); ^{13}C NMR (125 MHz, $CDCl_3$) δ 167.50, 166.14, 163.51, 152.20, 147.51, 143.83, 141.61, 139.23, 133.67, 128.70, 128.62, 128.50, 128.16, 128.03, 127.48, 126.24, 125.72, 120.71, 119.05, 117.13, 114.03, 96.57, 71.16, 71.07, 69.40, 65.25, 55.27, 52.28; HRMS found 720.2555, $C_{40}H_{37}N_3O_{10}$ requires $[M+H]^+$ 720.2551

H_2N-[*O*-MOM-hydroxyethyl(3-HABA)]-[*O*-(N-Trt)carbamoylmethoxy-(3-HABA)]-COOMe, S12

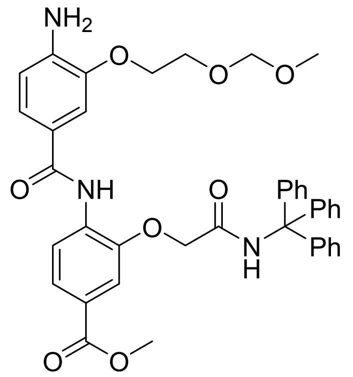

Using standard procedure C on 0.19 mmol scale to give the title compound as a colourless oil (74 mg, 56%). ν_{max}/cm^{-1} (solid state) 3343, 2950, 1673; ^{1}H NMR (500 MHz, $CDCl_3$) δ 8.57 (s, 1H, Amide N-H), 8.49 (d, *J* = 8.5 Hz, 1H, Ar-H), 7.73 (dd, *J* = 8.5, 1.1 Hz, 1H, Ar-H), 7.57 (s, 1H, Ar-H), 7.36 (d, *J* = 1.1, 1H, Ar-H), 7.25 (s, 1H, Ar-H), 7.15 (dd, *J* = 7.4, 2.8 Hz, 10H, Trityl), 7.05 (dd, *J* = 6.7, 2.7 Hz, 5H, Trityl), 6.45 (d, *J* = 8.1 Hz, 1H, Ar-H), 4.62 (s, 2H, O-C*H*$_2$-O), 4.61 (s, 2H, O-C*H*$_2$-CON), 4.20 (s, 2H, NH_2), 4.14 – 4.07 (t, *J* = 4.4 Hz, 2H, CH_2), 3.83 (s, 3H, OMe), 3.79 – 3.74 (t, *J* = 4.4 Hz, 2H, CH_2), 3.32 (s, 3H, OMe); ^{13}C NMR (125 MHz, $CDCl_3$) δ 166.36, 166.26, 165.19, 145.87, 145.79, 144.08, 141.14, 133.17, 128.46, 128.13, 127.31, 125.28, 125.00, 123.46, 120.81, 119.83, 113.51, 113.44, 111.73, 104.21, 96.64, 70.64, 69.46, 68.28, 66.14, 63.78, 55.33, 52.20.

H_2N-[*O*-hydroxyethyl(3-HABA)]-[*O*-carbamoylmethoxy-(3-HABA)]-COOH, 5

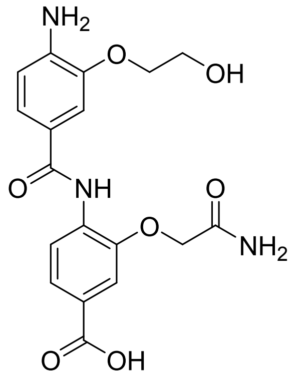

H_2N-[*O*-MOM-hydroxyethyl(3-HABA)]-[*O*-(N-Trt)carbamoylmethoxy-(3-HABA)]-COOMe (35 mg, 0.05 mmol) was dissolved in 1:1 tetrahydrofuran/methanol (5 ml) and treated with 1M sodium hydroxide solution (3 ml) for 2 hours. The reaction mixture was then acidified to pH 1 with concentrated HCl and stirred overnight. The reaction mixture was concentrated *in vacuo* and purified by mass directed preparative HPLC. v_{max}/cm^{-1} (solid state) 3408, 1663; ^{1}H NMR (500 MHz, d_6-DMSO) δ 9.67 (s, 1H, Amide N-H), 8.13 (d, *J* = 8.4 Hz, 1H, Ar-H), 7.62 (dd, *J* = 8.4, 1.8 Hz, 1H, Ar-H), 7.51 (d, *J* = 1.8 Hz, 1H, Ar-H), 7.45 (dd, *J* = 8.3, 1.9 Hz, 1H, Ar-H), 7.41 (d, *J* = 1.9 Hz, 1H, Ar-H), 6.67 (d, *J* = 8.2 Hz, 1H, Ar-H), 4.62 (s, 2H, CH_2), 4.01 (t, *J* = 4.8 Hz, 2H, CH_2), 3.75 (t, *J* = 4.7 Hz, 2H, CH_2); HRMS found 390.1301 $C_{18}H_{19}N_3O_7$ requires $[M+H]^+$ 390.1295

O_2N-[*O*-(N-Trt)carbamoylmethoxy-(3-HABA)]-[(3-HABA)]-COOMe, S13

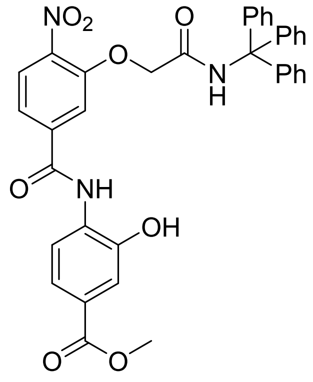

PyBOP (530 mg, 1.02 mmol) and diisopropylethylamine (0.4 ml, 2.12 mmol) were added sequentially to a solution of methyl 3-[(triphenylmethyl)carbamoyl]methoxy-4-aminobenzoate (410 mg, 0.85 mmol) in dichloromethane (30 ml) and the reaction stirred for 30 minutes. After which time methy 3-hydroxy-4-aminobenzoate (156 mg, 0.93 mmol) was added and the reaction stirred at r.t. overnight. The reaction mixture was concentrated *in vacuo* and redissolved in dimethylformamide (20 ml). Caesium carbonate (1.4 g, 4.25 mmol) was added and the reaction stirred at r.t. overnight. The reaction mixture was then poured into ethyl acetate (40 ml) and washed copiously with water and brine, dried over $MgSO_4$ and concentrated *in vacuo*. The residue was purified by column chromatography eluting with 5:95 ether/dichloromethane to give the *title compound* as a yellow solid (230 mg, 43%). v_{max}/cm^{-1} (solid state) 2956, 1709; ^{1}H NMR (500 MHz, DMSO-d_6) δ 8.74 (s, 1H, NH) 8.02 (d, *J* = 8.4 Hz, 1H, Ar-H), 7.93 (d, *J* = 8.4 Hz, 1H, Ar-H), 7.76 (d, *J* = 1.6 Hz, 1H, Ar-H), 7.67 (dd, *J* = 8.4, 1.6 Hz, 1H, Ar-H), 7.55 (d, *J* = 1.9 Hz, 1H, Ar-H), 7.50 (dd, *J* = 8.3, 1.9 Hz, 1H, Ar-H), 7.30 – 7.12 (m, 15H, trityl), 5.02 (s, 2H, 1-$C_\alpha H_2$), 3.83 (s, 3H, OMe); ^{13}C NMR (125 MHz, DMSO) δ 165.88, 165.83, 163.59, 150.39, 149.07, 144.31, 140.75, 139.25, 130.06, 128.38, 127.61, 126.62, 126.57, 125.29, 123.54, 120.30, 119.97, 115.86, 114.53, 69.40, 67.60, 52.03; HRMS found 632.2037 $C_{36}H_{29}N_3O_8$ requires $[M+H]^+$ 632.2027

O_2N-[*O*-(N-Trt)carbamoylmethoxy-(3-HABA)]-[*O*-MOM-hydroxyethyl (3-HABA)]-COOMe, S14

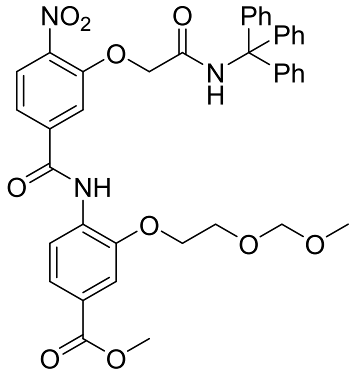

1-Bromo-2-(methoxymethoxy)ethane (41 µl, 0.35 mmol) was added to a suspension of O2N-[O-(N-Trt)carbamoylmethoxy-(3-HABA)]-[(3-HABA)]-COOMe (200 mg, 0.32 mmol) and potassium carbonate (218 mg, 1.58 mmol) in dimethylformamide (20 ml) and the reaction heated to 50 °C overnight. The reaction mixture was then poured into water (20 ml) and the extracted with ethyl acetate (30 ml). The organic layers were washed with brine, dried over MgSO4 and concentrated *in vacuo.* The residues was purified by column chromatography eluting with 40% ethyl acetate in petrol to give the *title compound* as a pale yellow solid (135 mg, 58%) ν_{max}/cm^{-1} (solid state) 3394, 2950, 1692, 1519; ^{1}H NMR (500 MHz, $CDCl_3$) δ 9.06 (s, 1H), 8.61 (d, *J* = 8.5 Hz, 1H), 8.21 (s, 1H), 8.12 (d, *J* = 8.4 Hz, 1H), 7.82 (dd, *J* = 8.5, 1.7 Hz, 1H), 7.73 (d, *J* = 1.6 Hz, 1H), 7.71 – 7.62 (m, 2H), 7.41 – 7.24 (m, 15H), 4.75 (s, 2H), 4.69 (s, 2H), 4.34 (t, *J* = 4.2 Hz, 2H), 3.98 (d, J = 4.2 Hz, 1H), 3.95 (s, 3H), 3.30 (s, 3H); ^{13}C NMR (126 MHz, $CDCl_3$) δ 166.41, 164.94, 162.50, 150.81, 144.21, 140.74, 140.61, 132.14, 128.68, 128.67, 128.06, 127.23, 126.82, 126.15, 124.17, 119.71, 119.29, 114.22, 113.33, 96.78, 70.65, 69.46, 68.29, 66.33, 55.42, 52.25; HRMS Found 720.2568 $C_{40}H_{37}N_3O_{10}$ $[M+H]^+$ requires 720.2552.

H_2N-[*O*-(N-Trt)carbamoylmethoxy-(3-HABA)]-[*O*-MOM-hydroxyethyl (3-HABA)]-COOMe, S15

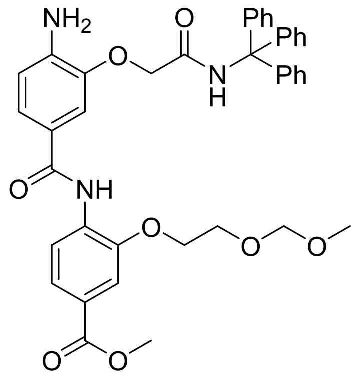

Using standard procedure C on 0.19 mmol scale to give the title compound as a colourless solid (62 mg, 50%) ν_{max}/cm^{-1} (solid state) 3422, 3360, 2923, 1699; ^{1}H NMR (500MHz, $CDCl_3$): δ 8.83 (s, 1H, Amide), 8.64 (d, *J* = 8.7 Hz, 1H, Amide), 7.77 (d, *J* = 8.5, 1.6 Hz, 1H, Ar-H), 7.62 (s, 2H, Ar-H), 7.40 - 7.50 (m, 2H, Ar-H), 7.12 - 7.31 (m, 15H, Trityl), 6.74 (d, *J* = 8.2 Hz, 1H), 4.66 (s, 4H, 1-$C_{\alpha}H_2$ and NH_2), 4.28 (t, J = 4.5 Hz, 2H, 2-$C_{\alpha}H_2$), 4.16 (s, 2H), 3.86 - 3.97 (m, 5H, 2-$C_{\beta}H_2$ and OMe), 3.27 (s, 3H, OMe); ^{13}C NMR (125 MHz, $CDCl_3$) δ 166.5, 164.5, 146.6, 144.3, 140.4, 133.3, 128.5, 128.1, 127.2, 124.7, 124.2, 122.7, 118.7, 114.3, 113.0, 112.0, 96.6, 70.4, 69.2, 68.5, 66.2, 55.3, 52.1; HRMS Found 690.2834 $C_{40}H_{39}N_3O_8$ $[M+H]^+$ requires 690.2810.

H_2N-[*O*-carbamoylmethoxy-(3-HABA)]-[*O*-hydroxyethyl-(3-HABA)]-COOH, S16

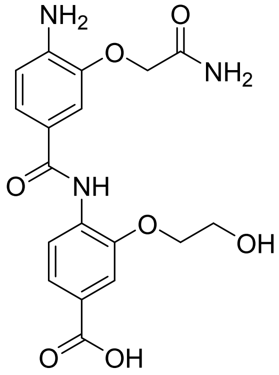

H_2N-[O-(N-Trt)carbamoylmethoxy-(3-HABA)]-[O-MOM-hydroxyethyl (3-HABA)]-COOMe (62 mg, 0.09 mmol) was dissolved in 1:1 tetrahydrofuran/methanol (10 ml) and treated with 1M sodium hydroxide solution (3 ml) for 2 hours. The reaction mixture was then acidified to pH 1 with concentrated HCl and stirred overnight. The product was observed by crude LS-MS but appeared to be unstable and could not be isolated.

Late Stage Derivatisaion

Methyl 4-nitro-3-(prop-2-en-1-yloxy)benzoate, S17

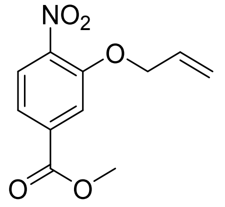

Allyl Bromide (610 µL, 7.10 mmol) was added to a solution of methyl 3-hydroxy-4-nitrobenzoate (1g, 5.07 mmol) and potassium carbonate (3.5 g, 25.35 mmol) in DMF (50 mL) and heated to 50 °C for 24 hours, allowed to cool and partitioned between EtOAc (50 mL) and H_2O (50 mL). The organic layer was separated and washed with H_2O (30 mL) and brine (30 mL), dried over $MgSO_4$, concentrated *in vacuo* to give a yellow solid (891 mg, 74%) ν_{max}/cm^{-1} (solid state) 2934, 1719, 1613; ^1H NMR (500 MHz, $CDCl_3$) δ 7.81 (d, *J* = 8.4 Hz, 1H, 5C-H), 7.72 (d, *J* = 1.7 Hz, 1H, 2C-H), 7.67 (dd, *J* = 8.4, 1.6 Hz, 1H, 6-CH), 6.03 (ddt, *J* = 17.3, 10.3, 5.0 Hz, 1H, 2'-CH), 5.49 (dq, *J* = 17.2, 1.6 Hz, 1H, 3'-CH_{trans}), 5.34 (dq, *J* = 10.6, 1.4 Hz, 1H, 3'-CH_{cis}), 4.73 (dt, *J* = 5.1, 1.6 Hz, 2H, 1'-CH_2), 3.95 (s, 3H, OMe);^{13}C NMR (125 MHz, $CDCl_3$) δ 165.10, 151.32, 142.62, 134.73, 131.26, 125.27, 121.51, 118.64, 115.86, 70.17, 52.80; HRMS found 260.0562, $C_{11}H_{11}NO_5$ $[M+Na]^+$ requires 260.0535.

Methyl 4-amino-3-(prop-2'-en-1'-yloxy)benzoate, S18

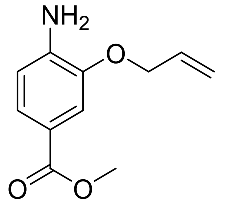

Prepared using standard procedure B on a 2.1 mmol scale (397 mg, 91%). ν_{max}/cm^{-1} (solid state) 3367, 2991, 1692; ^1H NMR (500 MHz, $CDCl_3$) δ 7.55 (dd, *J* = 8.2, 1.8 Hz, 1H, 6-CH), 7.46 (d, *J* = 1.7 Hz, 1H, 2-CH), 6.67 (d, *J* = 8.2 Hz, 1H, 5-CH), 6.08 (ddt, *J* = 17.2, 10.6, 5.4 Hz, 1H, 2'-CH), 5.42 (dq, *J* = 17.3, 1.6 Hz, 1H, 3'-CH_{trans})), 5.30 (dq, *J* = 10.5, 1.4 Hz, 1H, 3'-CH_{cis})), 4.61 (dt, *J* = 5.4, 1.4 Hz, 2H, 1-CH_2), 3.86 (s, 3H, OMe); ^{13}C NMR (125 MHz, $CDCl_3$) δ 167.27, 145.01, 141.38, 133.04, 124.28, 119.46, 117.88, 113.33, 112.68, 69.30, 51.68; HRMS found 208.0969, $C_{11}H_{13}NO_3$ $[M+H]^+$ requires 208.0968

4-Nitro-3-(prop-2'-en-1'-yloxy)benzoic acid, S19

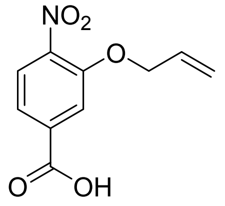

Prepared using standard procedure A on a 2.52 mmol scale (535 mg, 95%). ν_{max}/cm^{-1} (solid state) 2824, 2598, 2538, 1683; ^{1}H NMR (500 MHz, d_6-DMSO) δ 13.60 (s, 1H, OH), 7.96 (d, *J* = 8.3 Hz, 1H, 5-CH), 7.75 (d, *J* = 1.5 Hz, 1H, 2-CH), 7.63 (dd, *J* = 8.3, 1.6 Hz, 1H, 6-CH), 6.02 (ddt, *J* = 17.2, 10.5, 4.9 Hz, 1H, 2'-CH), 5.41 (dq, *J* = 17.3, 1.7 Hz, 1H, 3'C-H_{trans}), 5.29 (dq, *J* = 10.6, 1.5 Hz, 1H, 3'C-H_{cis}), 4.83 (dt, *J* = 4.9, 1.6 Hz, 2H, 1'-CH_2); ^{13}C NMR (125 MHz, d_6-DMSO) δ 165.71, 150.32, 142.18, 135.57, 132.32, 124.98, 121.47, 117.88, 115.73, 69.59; HRMS m/z (ESI) Found: 222.041033, $C_{10}H_9NO_5$ $[M-H]^-$ Requires 222.040796.

Trimers

The allyl bearing trimers were prepared in parallel using the methods and building blocks described previously[2-7] (Standard Procedures A, B, C and D), checking at pertinent times during the synthesis by crude NMR and LC-MS, to afford the below compounds. Compounds were either pure following final precipitation or purified by preparative HPLC.

O_2N-[*O*-Allyl(3-HABA)]-[*O*-*i*Bu (3-HABA)]-[*O*-*i*Pr (3-HABA)]-COOMe, 6a

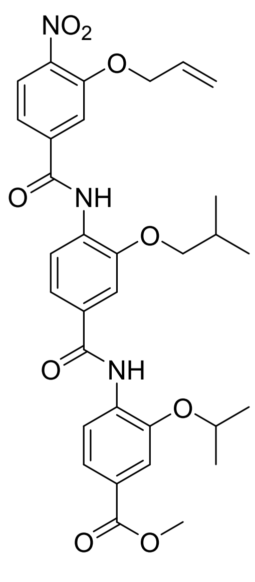

ν_{max}/cm^{-1} (solid state) 3421, 2975, 1704, 1680; ^{1}H NMR (500 MHz, $CDCl_3$) δ 8.85 (s, 1H, Amide N-H), 8.76 (s, 1H, Amide N-H), 8.63 (d, *J* = 8.4 Hz, 1H, Ar-H), 8.60 (d, *J* = 8.5 Hz, 1H, Ar-H), 7.93 (d, *J* = 8.3 Hz, 1H, Ar-H), 7.71 (dd, *J* = 8.5, 1.7 Hz, 1H, Ar-H), 7.69 (d, *J* = 1.5 Hz, 1H, Ar-H), 7.59 (m, 2H, Ar-H), 7.42 (m, 2H), 6.06 (ddt, *J* = 17.2, 10.2, 5.0 Hz, 1H, 1-C_βH), 5.52 (ddd, *J* = 17.1, 2.7, 1.5 Hz, 1H, 1-$C_\gamma H_{trans}$), 5.38 (ddd, *J* = 10.7, 2.5, 1.3 Hz, 1H1-$C_\gamma H_{cis}$), 4.83 – 4.67 (m, 3H, 1-$C_\alpha H_2$ and 3-C_αH), 3.98 (d, *J* = 6.5 Hz, 2H, 2- $C_\alpha H_2$), 3.90 (s, 3H, OMe), 2.22 (dp, *J* = 13.3, 6.7 Hz, 1H, 2-C_βH), 1.46 (d, *J* = 6.0 Hz, 6H, 3-$C_\beta H_3$ and 3-$C_\gamma H_3$), 1.11 (d, *J* = 6.7 Hz, 6H, 2-$C_\gamma H_3$ and 2-$C_\delta H_3$); ^{13}C NMR (125 MHz, $CDCl_3$) δ 166.74, 164.22, 162.98, 152.15, 147.84, 145.79, 141.96, 139.64, 132.93, 131.13, 130.68, 130.64, 126.02, 125.11, 123.31, 119.01, 118.93, 118.59, 117.71, 114.49, 113.14, 110.62, 75.26, 71.88, 70.31, 52.09, 28.25, 22.23, 19.31; HRMS found 628.2276, $C_{32}H_{35}N_3O_9$ $[M+Na]^+$ requires 628.2265

O_2N-[*O-i*Bu(3-HABA)]-[*O*-Allyl (3-HABA)]-[*O-i*Pr (3-HABA)]-COOMe

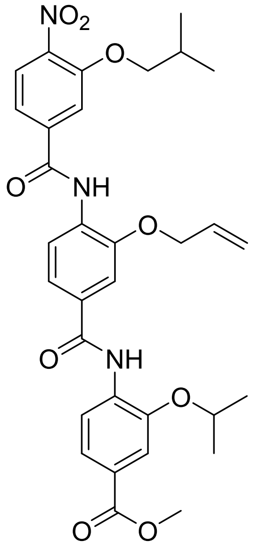

ν_{max}/cm^{-1} (solid state) 3428, 2961, 1792, 1714; ^{1}H NMR (500 MHz, $CDCl_3$) δ 8.81 (s, 1H, Amide N-H), 8.73 (s, 1H, Amide N-H), 8.61 (d, *J* = 8.4 Hz, 1H, Ar-H), 8.57 (d, *J* = 8.5 Hz, 1H, Ar-H), 7.88 (d, *J* = 8.3 Hz, 1H, Ar-H), 7.68 (dd, *J* = 8.5, 1.7 Hz, 1H, Ar-H), 7.63 (d, *J* = 1.6 Hz, 1H, Ar-H), 7.59 (d, *J* = 1.8 Hz, 1H, Ar-H), 7.57 (d, *J* = 1.7 Hz, 1H, Ar-H), 7.41 (dd, *J* = 8.5, 1.8 Hz, 1H, Ar-H), 7.38 (dd, *J* = 8.3, 1.7 Hz, 1H, Ar-H), 6.11 (ddt, *J* = 17.2, 10.6, 5.4 Hz, 1H, 2-C_{β}H), 5.47 (ddd, *J* = 17.3, 2.8, 1.5 Hz, 1H, 2-$C_{\gamma}H_{trans}$), 5.39 (ddd, *J* = 10.5, 2.3, 1.1 Hz, 1H, 2-$C_{\gamma}H_{cis}$), 4.86 – 4.68 (m, 3H, 2-$C_{\alpha}H_2$ and 3-C_{α}H), 3.94 (d, *J* = 6.5 Hz, 2H, 1-$C_{\alpha}H_2$), 3.88 (s, 3H, OMe), 2.16 (dt, *J* = 13.3, 6.6 Hz, 1H, 1-C_{β}H), 1.45 (d, *J* = 6.1 Hz, 6H, 3-$C_{\beta}H_3$ and 3-$C_{\gamma}H_3$), 1.06 (d, *J* = 6.7 Hz, 6H, 1-$C_{\gamma}H_3$ and 1-$C_{\delta}H_3$); ^{13}C NMR (125 MHz, $CDCl_3$) δ 166.69, 164.09, 163.23, 152.75, 147.39, 145.78, 141.70, 139.55, 132.88, 132.03, 130.82, 130.54, 125.81, 125.10, 123.24, 119.22, 119.19, 119.05, 118.58, 117.47, 113.95, 113.12, 111.06, 76.07, 71.87, 69.82, 52.06, 28.20, 22.22, 19.00; HRMS found 606.2456, $C_{32}H_{35}N_3O_9$ $[M+H]^+$ requires 606.2446

O_2N-[*O-i*Bu(3-HABA)]-[*O-i*Bu (3-HABA)]-[*O*-Allyl(3-HABA)]-COOMe, 6c

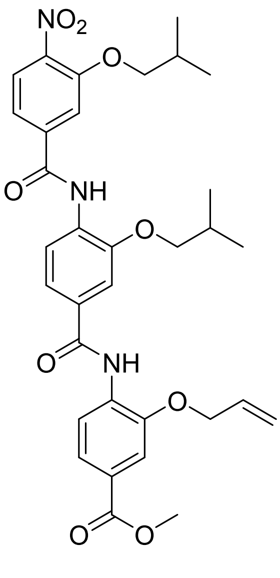

ν_{max}/cm^{-1} (solid state) 3435, 2956, 1714, 1679; ^{1}H NMR (500 MHz, $CDCl_3$) δ 8.83 (s, 1H, Amide N-H), 8.78 (s, 1H, Amide N-H), 8.66 (d, *J* = 8.4 Hz, 1H, Ar-H), 8.64 (d, *J* = 8.5 Hz, 1H, Ar-H), 7.94 (d, *J* = 8.3 Hz, 1H, Ar-H), 7.77 (dd, *J* = 8.5, 1.6 Hz, 1H, Ar-H), 7.66 (d, *J* = 1.4 Hz, 1H, Ar-H), 7.64 – 7.57 (m, 2H, Ar-H), 7.46 (dd, *J* = 8.4, 1.6 Hz, 1H, Ar-H), 7.42 (dd, *J* = 8.3, 1.5 Hz, 1H, Ar-H), 6.16 (ddt, *J* = 17.2, 10.6, 5.4 Hz, 1H, 3-C_{β}H), 5.52 (dd, *J* = 17.2, 1.2 Hz, 1H, 3-$C_{\gamma}H_{trans}$), 5.43 (dd, *J* = 10.5, 1.1 Hz, 1H, 3-$C_{\gamma}H_{cis}$), 4.75 (d, *J* = 5.4 Hz, 2H, 3-$C_{\alpha}H_2$), 4.04 – 3.95 (m, 4H, 1-$C_{\alpha}H_2$ and 2-$C_{\alpha}H_2$), 3.92 (s, 3H, OMe), 2.32 – 2.10 (m, 2H, 1-C_{β}H and 2-C_{β}H), 1.14 (d, *J* = 6.7 Hz, 6H, 1-$C_{\gamma}H_3$ and 1-$C_{\delta}H_3$), 1.10 (d, *J* = 6.7 Hz, 6H, 2-$C_{\gamma}H_3$ and 2-$C_{\delta}H_3$); ^{13}C NMR (125 MHz, $CDCl_3$) δ 166.63, 164.33, 163.08, 152.82, 147.78, 146.53, 141.72, 139.61, 132.27, 132.25, 130.77, 130.44, 125.92, 125.12, 123.71, 119.07, 118.89, 118.76, 118.62, 117.39, 113.74, 112.05, 110.54, 76.08, 75.20, 69.76, 52.12, 28.28, 28.22, 19.36, 19.03; HRMS found 620.2609, $C_{33}H_{37}N_3O_9$ $[M+H]^+$ requires 620.2602

O_2N-[(3-HABA)]-[*O-i*Bu (3-HABA)]-[*O-i*Pr (3-HABA)]-COOMe, 7a

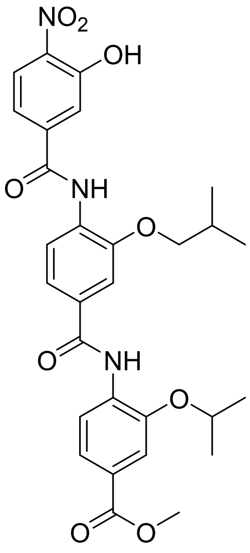

Prepared using standard procedure E on 0.58 mmol scale (267 mg, 81%). ^1H NMR (500 MHz, $CDCl_3$) δ 10.61 (s, 1H, OH), 8.87 (s, 1H, Amide NH), 8.77 (s, 1H, Amide NH), 8.64 (m, 2H, Ar-H), 8.28 (d, *J* = 8.7 Hz, 1H, Ar-H), 7.74 (dd, *J* = 8.5, 1.7 Hz, 1H, Ar-H), 7.68 (d, *J* = 1.9 Hz, 1H, Ar-H), 7.63 (d, *J* = 1.8 Hz, 1H, Ar-H), 7.61 (d, *J* = 1.7 Hz, 1H, Ar-H), 7.47 (dd, *J* = 8.8, 1.9 Hz, 1H, Ar-H), 7.44 (dd, *J* = 8.5, 1.8 Hz, 1H, Ar-H), 4.78 (hept, *J* = 6.2 Hz, 1H, 3-C_αH), 4.00 (d, *J* = 6.6 Hz, 2H, 2-$C_\alpha H_2$), 3.92 (s, 3H, OMe), 2.24 (dp, *J* = 13.4, 6.6 Hz, 1H, 2-C_βH), 1.46 (d, *J* = 6.1 Hz, 6H3-$C_\beta H_3$ and 3-$C_\gamma H_3$), 1.12 (d, *J* = 6.7 Hz, 6H, 2-$C_\gamma H_3$ and 2-$C_\delta H_3$); HRMS Found: 566.212; $C_{29}H_{31}N_3O_9$ $[M+H]^+$ requires 566.2133

O_2N-[*O-i*Bu(3-HABA)]-[(3-HABA)]-[*O-i*Pr(3-HABA)]-COOMe, 7b

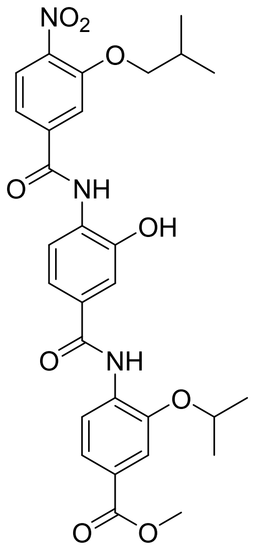

Prepared using standard procedure E on a 0.4 mmol scale (168 mg, 75%). ^1H NMR (500 MHz, d_6-DMSO) δ 10.34 (s, 1H, OH), 9.86 (s, 1H, Amide NH), 9.22 (s, 1H, Amide NH), 8.25 (d, *J* = 8.4 Hz, 1H, Ar-H), 8.00 (d, *J* = 8.3 Hz, 1H, Ar-H), 7.88 (d, *J* = 8.3 Hz, 1H, Ar-H), 7.83 (d, *J* = 1.5 Hz, 1H, Ar-H), 7.66 – 7.60 (m, 2H, Ar-H), 7.58 (d, *J* = 1.7 Hz, 1H, Ar-H), 7.49 (d, *J* = 2.0 Hz, 1H, Ar-H), 7.44 (dd, *J* = 8.3, 2.0 Hz, 1H, Ar-H), 4.82 – 4.69 (m, 1H, 3-C_αH), 4.05 (d, *J* = 6.4 Hz, 2H, 1-$C_\alpha H_2$), 3.33 (s, 3H, OMe) 2.06 (sept, 6.6 Hz, 1H, 1-C_βH), 1.36 (d, *J* = 6.0 Hz, 6H, 3-$C_\beta H_3$ and 3-$C_\gamma H_3$), 0.99 (d, *J* = 6.7 Hz, 6H, 1-$C_\gamma H_3$ and 1-$C_\delta H_3$); HRMS Found 566.2126; $C_{29}H_{31}N_3O_9$ $[M+H]^+$ requires 566.2133.

O_2N-[*O*-*i*Bu(3-HABA)] *O*-*i*Bu(3-HABA)]-[(3-HABA)]-COOMe, 7c

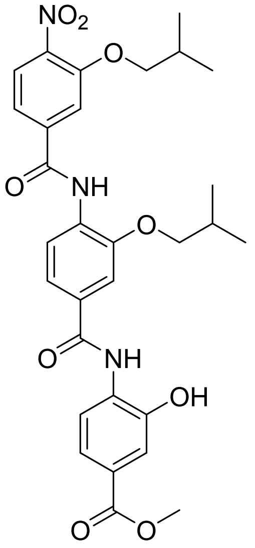

Prepared using standard procedure E on a 1 mmol scale (442 mg, 74%). ^{1}H NMR (500 MHz, d_6-DMSO) δ 10.37 (s, 1H, OH), 9.81 (s, 1H, Amide NH), 9.54 (s, 1H, Amide NH), 8.02 (d, *J* = 8.3 Hz, 1H, Ar-H), 7.96 (app.t, *J* = 8.0 Hz, 2H, Ar-H), 7.80 (d, *J* = 1.5 Hz, 1H, Ar-H), 7.66 (d, *J* = 1.8 Hz, 1H, Ar-H), 7.64 – 7.59 (m, 2H, Ar-H), 7.53 (d, *J* = 2.0 Hz, 1H, Ar-H), 7.48 (dd, *J* = 8.3, 1.9 Hz, 1H, Ar-H), 4.03 (d, *J* = 6.5 Hz, 2H, 1-$C_\alpha H_2$), 3.93 (d, *J* = 6.4 Hz, 2H, 2-$C_\alpha H_2$), 3.83 (s, 3H, OMe), 2.17 – 1.97 (m, 2H, 1-C_βH and 2-C_βH), 1.01 (d, *J* = 6.8 Hz, 6H, 1-$C_\gamma H_3$ and 1-$C_\delta H_3$), 0.99 (d, *J* = 6.8 Hz, 6H, 2-$C_\gamma H_3$ and 2-$C_\delta H_3$); HRMS Found 580.2288; $C_{30}H_{33}N_3O_9$ $[M+H]^+$ requires 580.2289.

H_2N-[(3-HABA)]-[*O*--^{i}Bu(3-HABA)]-[*O*-^{i}Pr(3-HABA)]-COOH, 8a

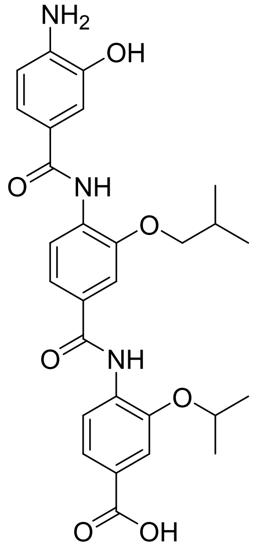

Prepared by standard procedures C and A and purification by mass directed HPLC (14.4 mg, 62%). ^{1}H NMR (500 MHz, d_6-DMSO) δ 9.30 (s, 1H, Amide NH), 8.86 (s, 1H, Amide NH), 8.28 (d, *J* = 8.2 Hz, 1H, Ar-H), 8.16 (d, *J* = 8.3 Hz, 1H, Ar-H), 7.61 – 7.53 (m, 4H), 7.27 (d, *J* = 2.1 Hz, 1H, Ar-H), 7.19 (dd, *J* = 8.3, 2.1 Hz, 1H, Ar-H), 6.64 (d, *J* = 8.2 Hz, 1H, Ar-H), 4.76 – 4.67 (m, 1H, 3-C_αH), 3.97 (d, *J* = 6.6 Hz, 2H, 2-$C_\alpha H_2$), 1.90 – 1.79 (m, 1H, 2-C_βH), 1.35 (d, *J* = 6.0 Hz, 6H, 3-$C_\beta H_3$ and 3-$C_\gamma H_3$), 1.03 (d, *J* = 6.7 Hz, 6H, 2-$C_\gamma H_3$ and 2-$C_\delta H_3$); HRMS found 522.2244 $C_{28}H_{31}N_3O_7$ $[M+H]^+$ requires 522.2234

H_2N-[2-(pyrrolidin-1-yl)ethoxy(3-HABA)]-[*O*--^{i}Bu(3-HABA)]-[*O*—^{i}Pr(3-HABA)]-COOH, 8b

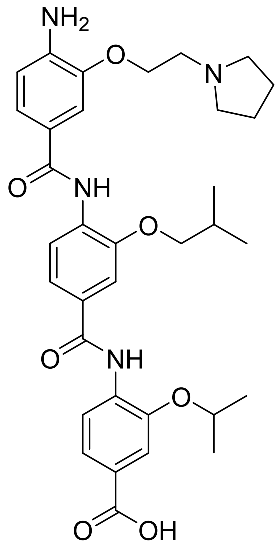

Prepared by standard procedure F followed by standard procedures C and A and purification by mass directed HPLC (7.8 mg, 36%). ^{1}H NMR (500 MHz, d_6-DMSO) δ 9.16 (s, 1H, Amide NH), 9.00 (s, 1H, Amide NH), 8.50 (s, 1H, Ar-H), 8.20 (d, *J* = 8.4 Hz, 1H, Ar-H), 7.89 (d, *J* = 7.8 Hz, 1H, Ar-H), 7.56 (m, 3H, Ar-H), 7.46 (s, 1H, Ar-H), 7.37 (d, *J* = 7.2 Hz, 1H, Ar-H), 6.70 (d, *J* = 8.6 Hz, 1H, Ar-H), 4.61 (dt, *J* = 12.1, 6.1 Hz, 1H, 3-C_αH), 4.10 (t, *J* = 6.0 Hz, 2H, 1-$C_\beta H^2$), 3.95 (d, *J* = 6.5 Hz, 2H, 2-$C_\beta H_2$), 2.83 (t, *J* = 6.0 Hz, 1H, 1-$C_\alpha H_2$), 2.58 – 2.51 (m, 4H, pyrrolidine 2 × CH_2) 2.14 (dt, *J* = 13.1, 6.5 Hz, 1H, 2-C_βH), 1.73 – 1.65 (m, 4H, pyrrolidine 2 × CH_2) 1.31 (d, *J* = 6.0 Hz, 6H, 3-$C_\beta H_3$ and 3-$C_\gamma H_3$), 1.25 – 1.09 (m, 5H), 1.08 – 0.97 (d, *J* = 6.7 Hz, 6H, 2-$C_\gamma H_3$ and 2-$C_\delta H_3$). HRMS Found 619.3136 $C_{34}H_{42}N_4O_7$ $[M+H]^+$ requires 619.3126

H_2N-[*O*-^{i}Bu(3-HABA)]-[(3-HABA)]-[*O*-^{i}Pr(3-HABA)]-COOH, 8c

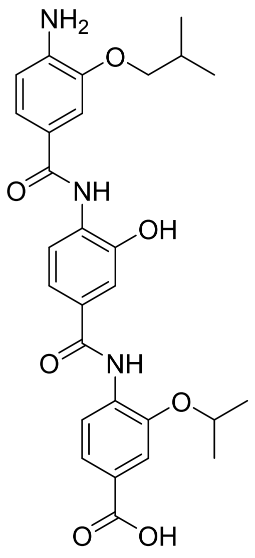

Prepared by standard procedures C and A and purification by mass directed HPLC (11.8 mg, 51%). ^{1}H NMR (500 MHz, d_6-DMSO) δ 9.26 (s, 1H, Amide NH), 9.15 (s, 1H, Amide NH), 8.22 (d, *J* = 8.3 Hz, 1H, Ar-H), 7.99 (d, J = 8.4 Hz, 1H), 7.63 – 7.52 (m, 4H) 7.47 (d, *J* = 2.0 Hz, 1H), 7.42 (dd, *J* = 8.1, 1.7 Hz, 1H), 6.70 (d, *J* = 8.0 Hz, 1H), 4.77 – 4.69 (m, 1H, 3-C_αH), 3.81 (d, *J* = 6.5 Hz, 2H, 1-$C_\alpha H_2$), 2.13 – 2.02 (m, 1H, 1-C_βH), 1.36 (d, *J* = 6.0 Hz, 6H, 3-$C_\beta H_3$ and 3-$C_\gamma H_3$), 1.02 (d, *J* = 6.7 Hz, 6H, 1-$C_\gamma H_3$ and 1-$C_\delta H_3$); HRMS found 522.2241 $C_{28}H_{31}N_3O_7$ $[M+H]^+$ requires 522.2234

H_2N-[*O*--^{i}Bu(3-HABA)]-[3-(dimethylamino)propoxy (3-HABA)]-[*O*-^{i}Pr(3-HABA)]-COOH, 8d

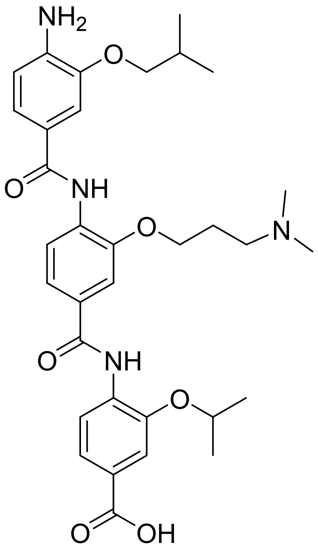

Prepared by standard procedure F followed by standard procedures C and A and purification by mass directed HPLC (11 mg, 49%). ^{1}H NMR (500 MHz, d_6-DMSO) δ 9.28 (s, 1H, Amide N-H), 9.04 (s, 1H, Amide N-H), 8.22 (d, J = 8.3 Hz, 1H, Ar-H), 8.11 (d, J = 8.5 Hz, 1H, Ar-H), 7.64 – 7.55 (m, 5H, Ar-H), 7.37 – 7.33 (m, 1H, Ar-H) 6.70 (d, J = 8.0 Hz, 1H, Ar-H), 4.74 – 4.66 (m, 1H, 3-C_αH), 4.20 (t, J = 6.2 Hz, 2H, 2-$C_\alpha H_2$), 3.80 (t, J = 5.4 Hz, 2H, 2-$C_\gamma H_2$), 2.41 (m, 2H, 2-$C_\beta H_2$), 2.14 (s, 6H, 2 × NMe), 1.97 (m, 1H, 1-C_βH) 1.35 (dd, J = 6.0, 1.8 Hz, 6H, 3-$C_\beta H_3$ and 3-$C_\gamma H_3$), 1.02 (dd, J = 6.7, 3.1 Hz, 6H, 1-$C_\gamma H_3$ and 1-$C_\delta H_3$); HRMS Found 607.3138, $C_{33}H_{42}N_4O_7$ $[M+H]^+$ requires 607.3126.

H_2N-[*O*--^{i}Bu(3-HABA)]-[2-(pyrrolidin-1-yl)ethoxy (3-HABA)] -[*O*-^{i}Pr(3-HABA)]-COOH, 8e

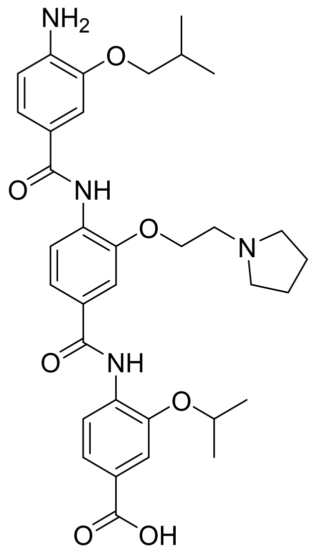

Prepared by standard procedure F followed by standard procedures C and A and purification by mass directed HPLC (6.2 mg, 29%). ^{1}H NMR (500 MHz, d_6-DMSO) δ 9.28 (s, 1H, Amide NH), 9.26 (s, 1H, Amide NH), 8.39 – 8.25 (m, 2H, Ar-H), 8.08 (d, J = 9.6 Hz, 1H, Ar-H), 7.72 – 7.50 (m, 5H, Ar-H), 7.33 (m, 1H, Ar-H), 4.74 – 4.63 (m, 1H, 3-C_αH), 4.29 (m, 2H, 2-$C_\beta H_2$), 3.80 (d, J =6.5 Hz, 2H, 1-$C_\alpha H_2$), 2.84 (m, 2H, 2-$C_\alpha H_2$), 2.09 (m, 1H, 1-C_βH), 1.60 – 1.50 (m, 4H, pyrrolidine 2 × CH_2), 1.35 (d, J = 6.0 Hz, 6H, 3-$C_\beta H_3$ and 3-$C_\gamma H_3$), 1.03 (d, J = 6.7 Hz, 6H, 1-$C_\gamma H_3$ and 1-$C_\delta H_3$); HRMS Found 619.3142, $C_{34}H_{42}N_4O_7$ $[M+H]^+$ requires 619.3126j

H_2N-[*O*-^{i}Bu(3-HABA)]-[*O*-^{i}Bu(3-HABA)]-[(3-HABA)]-COOH, 8f

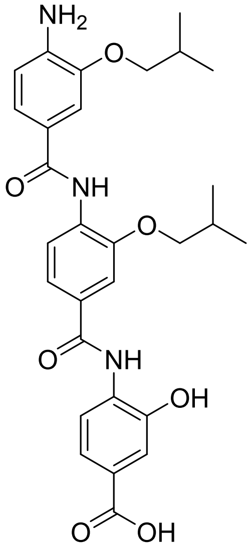

Prepared by standard procedures C and A and purification by mass directed HPLC (5.5 mg, 20%). ^{1}H NMR (500 MHz, d_6-DMSO) δ 9.47 (s, 1H, Amide NH), 8.99 (s, 1H, Amide NH), 8.24 (d, J = 8.3 Hz, 1H, Ar-H), 7.92 (d, J = 8.3 Hz, 1H, Ar-H), 7.64 – 7.58 (m, 2H, Ar-H), 7.50 (d, J = 1.9 Hz, 1H, Ar-H), 7.45 (dd, J = 8.3, 1.9 Hz, 1H, Ar-H), 7.35 (dd, J = 8.2, 1.9 Hz, 1H, Ar-H), 7.31 (d, J = 1.9 Hz, 1H, Ar-H), 6.70 (d, J = 8.2 Hz, 1H, Ar-H), 3.96 (d, J = 6.3 Hz, 2H, 1-$C_{\alpha}H_2$), 3.79 (d, J = 6.5 Hz, 2H, 2-$C_{\alpha}H_2$), 2.18 – 2.01 (m, 2H, 1-C_{β}H and 2-C_{β}H), 1.05 (d, J = 6.7 Hz, 6H, 1-$C_{\gamma}H_3$ and 1-$C_{\delta}H_3$), 1.02 (d, J = 6.7 Hz, 6H, 2-$C_{\gamma}H_3$ and 2-$C_{\delta}H_3$); HRMS found 536.23994 $C_{29}H_{33}N_3O_7$ $[M+H]^+$ requires 536.2391

H_2N-[*O*-^{i}Bu (3-HABA)]- [*O*--^{i}Bu(3-HABA)]- [3-(dimethylamino)propoxy (3-HABA)]-COOH, 8g

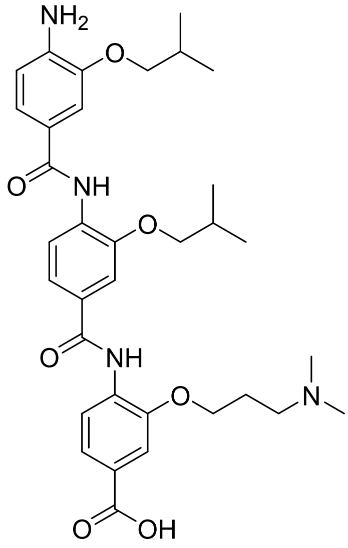

Prepared by standard procedure F followed by standard procedures C and A and purification by mass directed HPLC (15 mg, 70%). ^{1}H NMR (500 MHz, d_6-DMSO) δ 9.50 (s, 1H, Amide N-H), 9.00 (s, 1H, Amide N-H), 8.25 (d, J = 8.3 Hz, 1H, Ar-H), 8.00 (t, J = 9.2 Hz, 1H, Ar-H), 7.64 – 7.59 (m, 3H, Ar-H), 7.58 (d, J = 1.6 Hz, 1H, Ar-H), 7.35 (dd, J = 8.2, 1.9 Hz, 1H, Ar-H), 7.29 (d, J = 1.8 Hz, 1H, Ar-H), 6.69 (d, J = 8.2 Hz, 1H, Ar-H), 4.19 (t, J = 5.9 Hz, 2H, 3-$C_{\alpha}H_2$), 3.10 – 2.99 (m, 3H, 3-$C_{\gamma}H_2$), 2.64 – 2.57 (s, 6H, 2 × NMe), 2.19 – 2.01 (m, 4H, 1-C_{β}H, 2-C_{β}H and 3-$C_{\beta}H_2$), 1.05 (t, J = 5.5 Hz, 6H, 1-$C_{\gamma}H_3$ and 1-$C_{\delta}H_3$), 1.01 (d, J = 4.8 Hz, 6H, 2-$C_{\gamma}H_3$ and 2-$C_{\delta}H_3$); HRMS Found 621.3293 $C_{34}H_{44}N_2O_7$ $[M+H]^+$ requires 621.3288.

H_2N-[*O*-^{i}Bu (3-HABA)] -[*O*-^{i}Bu(3-HABA)] -[2-(pyrrolidin-1-yl) ethoxy(3-HABA)]-COOH, 8h

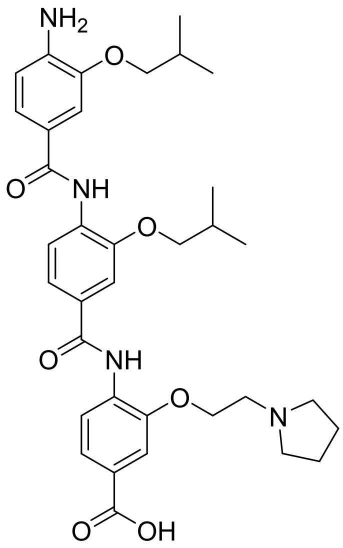

Prepared by standard procedure F followed by standard procedures C and A and purification by mass directed HPLC (16 mg, 72%). ^{1}H NMR (500 MHz, d_6-DMSO) δ 9.83 (s, 1H, Amide-NH), 8.99 (s, 1H, Amide-NH), 8.25 (d, *J* = 8.4 Hz, 1H, Ar-H), 8.04 (d, *J* = 8.3 Hz, 1H, Ar-H), 7.77 (s, 1H, Ar-H), 7.69 (dd, *J* = 8.4, 1.7 Hz, 1H, Ar-H), 7.64 (dd, *J* = 8.3, 1.4 Hz, 1H, Ar-H), 7.61 (d, *J* = 1.5 Hz, 2H, Ar-H), 7.35 (dd, *J* = 8.3, 1.6 Hz, 1H, Ar-H), 7.30 (d, *J* = 1.7 Hz, 1H, Ar-H), 6.71 (d, *J* = 8.2 Hz, 1H, Ar-H), 4.45 (t, *J* = 4.4 Hz , 2H, 3-$C_\alpha H_2$), 4.01 (d, *J* = 6.3 Hz, 2H, 1-$C_\alpha H_2$), 3.79 (d, *J* = 6.5 Hz, 2H, 2-$C_\alpha H_2$), 3.59 (m, 2H, 3-$C_\beta H_2$), 2.15 (dt, *J* = 13.5, 6.6 Hz, 1H, 1-C_βH), 2.08 (dt, *J* = 13.2, 6.7 Hz, 1H, 2-C_βH),1.93-1.86 (m, 4H, pyrrolidine 2 × CH_2), 1.27 – 1.22 (m, 4H, pyrrolidine 2 × CH_2), 1.05 (d, *J* = 6.7 Hz, 6H, 1-$C_\gamma H_3$ and 1-$C_\delta H_3$), 1.02 (d, *J* = 6.7 Hz, 6H, 2-$C_\gamma H_3$ and 2-$C_\delta H_3$); HRMS Found 633.3294 $C_{35}H_{44}N_2O_7$ $[M+H]^+$ requires 633.3282

H_2N-[*O*-^{i}Bu (3-HABA)]- [*O*--^{i}Bu(3-HABA)]- [pyridin-4-ylmethoxy (3-HABA)]-COOH, 8i

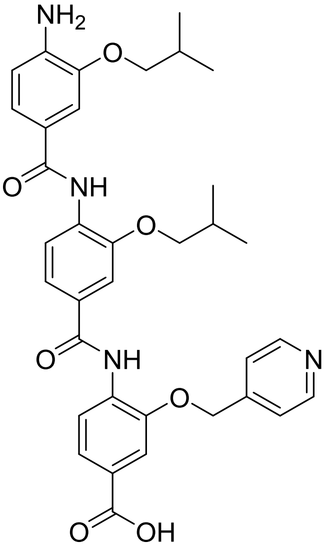

Prepared by standard procedure F followed by standard procedures C and A and purification by mass directed HPLC (8.7 mg, 40%). ^{1}H NMR (500 MHz, d_6-DMSO) δ 9.57 (s, 1H, Amide NH), 8.99 (s, 1H, Amide NH), 8.22 (d, *J* = 8.4 Hz, 1H, Ar-H), 8.04 (d, *J* = 8.3 Hz, 1H, Ar-H), 8.01 (d, *J* = 7.9 Hz, 1H, Ar-H), 7.71 (d, *J* = 1.7 Hz, 1H, Ar-H), 7.66 – 7.52 (m, 4H, Ar-H), 7.51 (d, *J* = 1.9 Hz, 1H, Ar-H), 7.44 (dt, *J* = 7.6, 3.8 Hz, 1H, Ar-H), 7.35 (dd, *J* = 8.2, 1.9 Hz, 1H, Ar-H), 7.31 (t, *J* = 2.0 Hz, 1H, Ar-H), 6.71 (d, *J* = 8.2 Hz, 1H, Ar-H), 5.41 (s, 2H, 3-$C_\alpha H_2$), 3.90 (d, *J* = 6.2 Hz, 2H, , 1-$C_\alpha H_2$), 3.79 (d, *J* = 6.5 Hz, 2H, , 2-$C_\alpha H_2$), 2.19 – 2.03 (m, 2H, 1-C_βH and 2-C_βH), 1.04 (d, *J* = 6.7 Hz, 6H, 1-$C_\gamma H_3$ and 1-$C_\delta H_3$), 1.02 (d, *J* = 6.7 Hz, 6H, 2-$C_\gamma H_3$ and 2-$C_\delta H_3$); HRMS Found 627.2820 $C_{35}H_{38}N_4O_7$ $[M+H]^+$ requires 627.2813

H_2N-[*O*-^{i}Bu(3-HABA)]-[*O-TFD* (3-HABA)]-[*O*-^{i}Pr(3-HABA)]-COOH, 8j

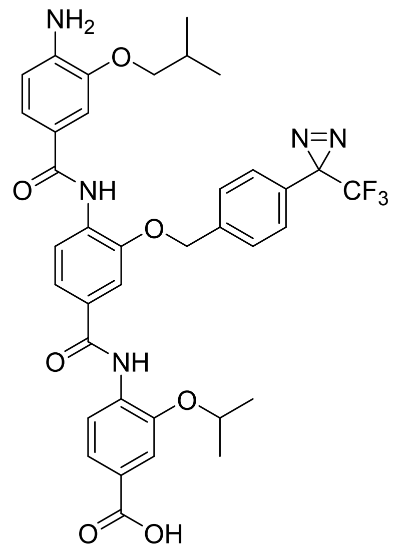

Prepared following standard procedure E followed by standard procedure C. The resulting material was dissolved in THF and cooled to 0 °C and sodium hydride (1 eq.) added. The reaction mixture was stirred for 30 minutes at 0 °C before 3-[4-(iodomethyl)phenyl]-3-(trifluoromethyl)-3H-diazirine **9** (prepared using literature methods)[8] (1.1 eq.) was added. The reaction mixture was stirred overnight and allowed to warm to r.t. The reaction mixture was then diluted with methanol and treated with 1M NaOH (aq.). Finally the reaction mixture was concentrated *in vacuo* and purified by mass directed HPLC (2.05 mg, 16%). ^{1}H NMR (500 MHz, $CDCl_3$) δ 8.82 (s, 1H, Amide NH), 8.64 (d, J = 8.3 Hz, 1H, Ar-H), 8.57 (m, 2H, Amide NH and Ar-H), 7.71 (d, J = 8.5 Hz, 1H, Ar-H), 7.63 (s, 1H, Ar-H), 7.55 (s, 1H, Ar-H), 7.44 (d, J = 8.2 Hz, 2H, Ar-H), 7.37 (d, J = 9.1 Hz, 1H, Ar-H), 7.33 (s, 1H, Ar-H), 7.08 (d, J = 7.7 Hz, 1H, Ar-H), 6.60 (d, J = 8.1 Hz, 1H, Ar-H), 5.21 (s, 2H, 2-$C_\alpha H_2$), 4.73 – 4.64 (m, 1H, 3-C_αH), 3.75 (d, J = 6.4 Hz, 2H, 1-$C_\alpha H_2$), 2.11 – 2.04 (m, 1H, 1-C_βH), 1.39 (d, J = 6.0 Hz, 6H, 3-$C_\beta H_3$ and 3-$C_\gamma H_3$), 0.97 (t, J = 9.0 Hz, 6H, 2-$C_\gamma H_3$ and 2-$C_\delta H_3$); HRMS Found: 720.2639; $C_{37}H_{36}F_3N_5O_7$ $[M+H]^+$ requires 720.2639.

H_2N-[*O*--^{i}Bu(3-HABA)]-[*O*-^{i}Bu(3-HABA)]-[*O*-*TFD*(3-HABA)]-COOH, 8k

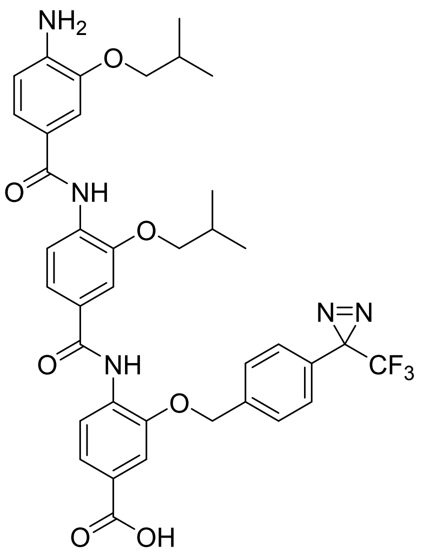

Prepared following standard procedure E followed by standard procedure C. The resulting material was dissolved in THF and cooled to 0 °C and sodium hydride (1 eq.) added. The reaction mixture was stirred for 30 minutes at 0 °C before 3-[4-(iodomethyl)phenyl]-3-(trifluoromethyl)-3H-diazirine **9** (prepared using literature methods)[8] (1.1 eq.) was added. The reaction mixture was stirred overnight and allowed to warm to r.t. The reaction mixture was then diluted with methanol and treated with 1M NaOH (aq.). Finally the reaction mixture was concentrated *in vacuo* and purified by mass directed HPLC (4.6 mg, 44%). ^{1}H NMR (500 MHz, $CDCl_3$) δ 8.79 (s, 1H, Amide NH), 8.76 (s, 1H, Amide NH), 8.72 (d, J = 8.5 Hz, 1H, Ar-H), 8.68 (d, J = 8.4 Hz, 1H, Ar-H), 7.88 (d, J = 8.6 Hz, 1H, Ar-H), 7.72 (s, 1H, Ar-H), 7.58 (s, 1H, Ar-H), 7.53 (d, J = 8.2 Hz, 2H, Ar-H), 7.44 (s, 1H, Ar-H), 7.40 – 7.33 (m, 2H, Ar-H), 7.30 (d, J = 3.8 Hz, 2H, Ar-H), 6.78 (d, J = 8.1 Hz, 1H, Ar-H), 5.31 (s, 2H, 3-$C_\alpha H_2$), 3.96 (d, J = 6.4 Hz, 2H, 1-$C_\alpha H_2$), 3.90 (d, J = 6.5 Hz, 2H, 2-$C_\alpha H_2$), 2.31 – 2.04 (m, 2H, 1-C_βH and 2-C_βH), 1.14 (d, J = 6.7 Hz, 6H, 1-$C_\gamma H_3$ and 1-$C_\delta H_3$), 1.10 (d, J = 6.7 Hz, 6H, 2-$C_\gamma H_3$ and 2-$C_\delta H_3$); HRMS Found: 734.2780; $C_{38}H_{38}F_3N_5O_7$ $[M+H]^+$ requires 734.2796.

Competition Assays

Competition assays and protein expression were carried out as previously reported.[2, 9]

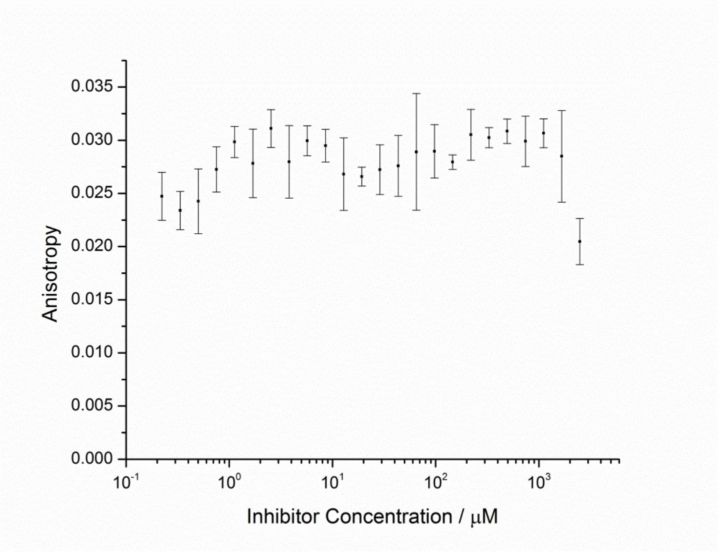

Figure S1. Inhibition profile of helix 2 mimetic compound **5**

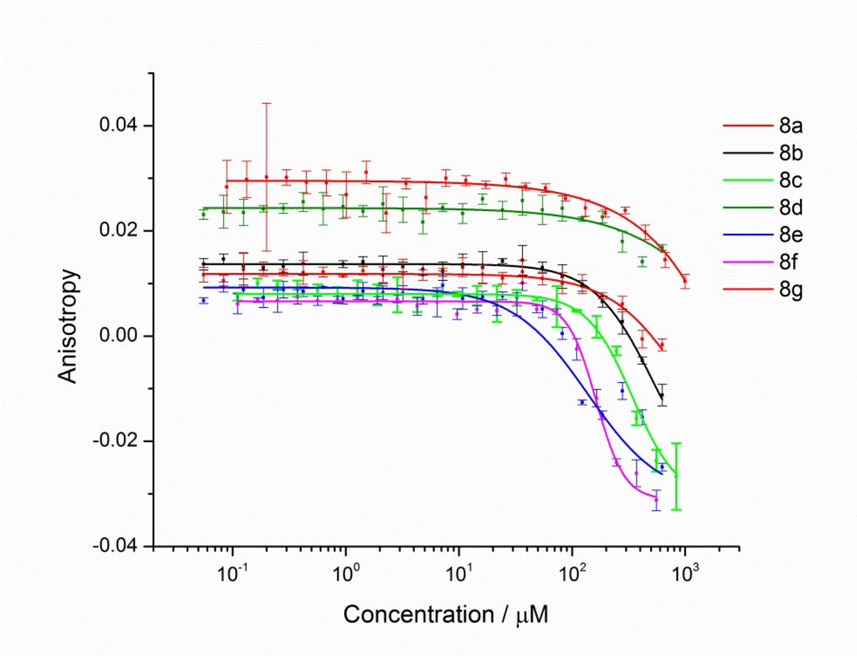

Figure S2. Inhibition curves for compounds **8a-g**

Photo-Crosslinking Experiments

A solution containing the photo-crosslinking compound (150 µM) and p300 (100 µM) in assay buffer was prepared and analysed by LC-MS on a Bruker HCT Ultra. Separate solutions containing only compound or only protein were treated identically. The solutions were then irradiated with UV light (365 nm) for 1 hour whilst cooled in an ice bath. The LC-MS analysis was then repeated. The protein signals were examined for any increase of adduct formation.

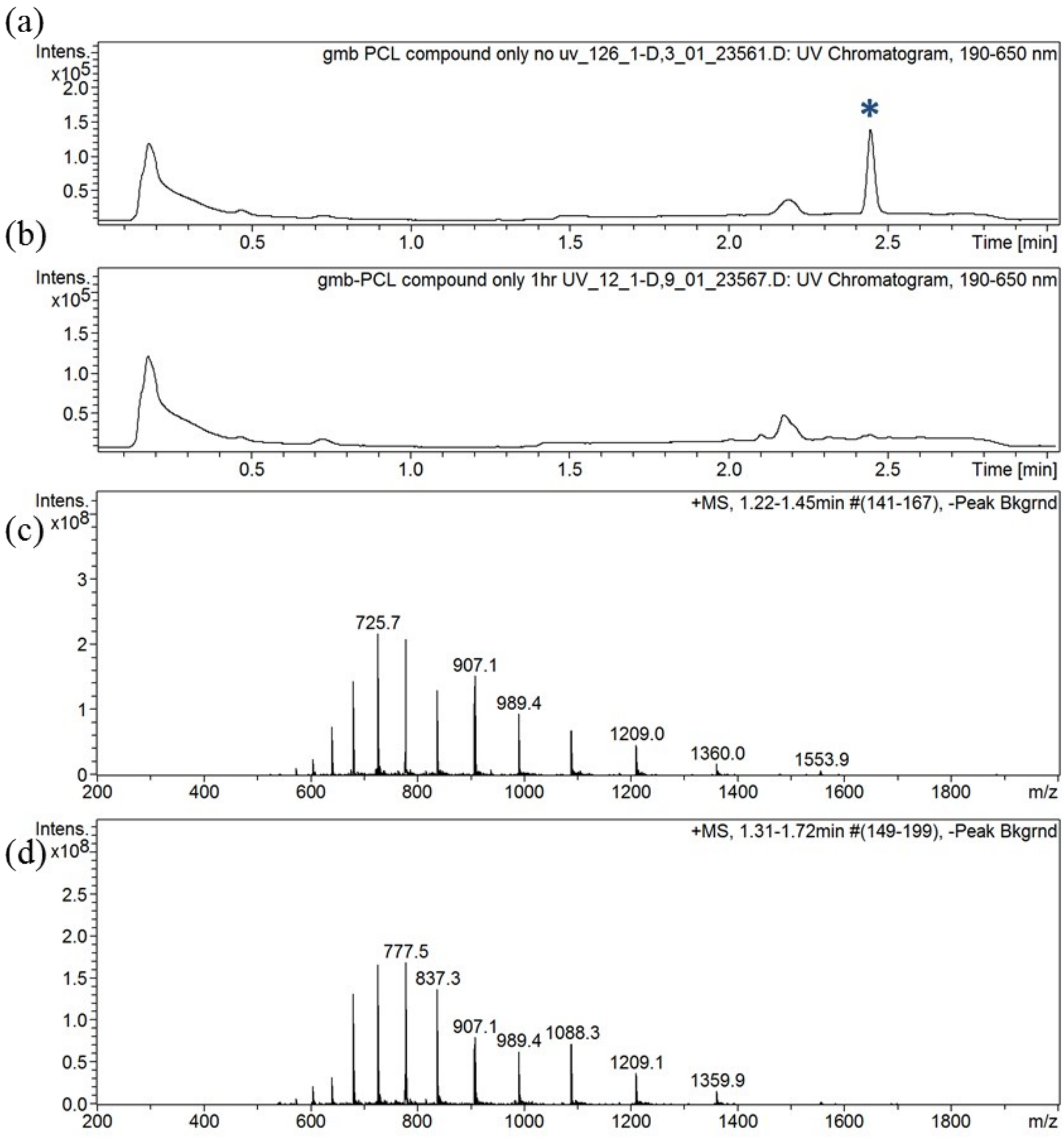

Figure S3 - Analytical data for photo-crosslinking experiments. (a) Chromatogram of 150 µM compound **8k** in buffer prior to UV irradiation. The peak corresponding to compound **129** is denoted by the asterisk (b) Chromatogram of 150 µM compound **8k** in buffer post UV irradiation (365 nm, 1 hour) (c) mass spectrum of protein before UV irradiation (365 nm, 1 hour) (d) mass spectrum of protein after UV activation (365 nm, 1 hour)

Table S2 – Calculated and Observed m/z values for p300

Charge State	Calculated m/z	Observed m/z
8^+	1359.8	1359.9
9^+	1208.8	1209.1
10^+	1088.1	1088.3
11^+	989.2	98934
12^+	906.9	607.1
13^+	837.2	837.3
14^+	777.5	777.5
15^+	725.7	725.7

NMR and HPLC Spectra for final compounds

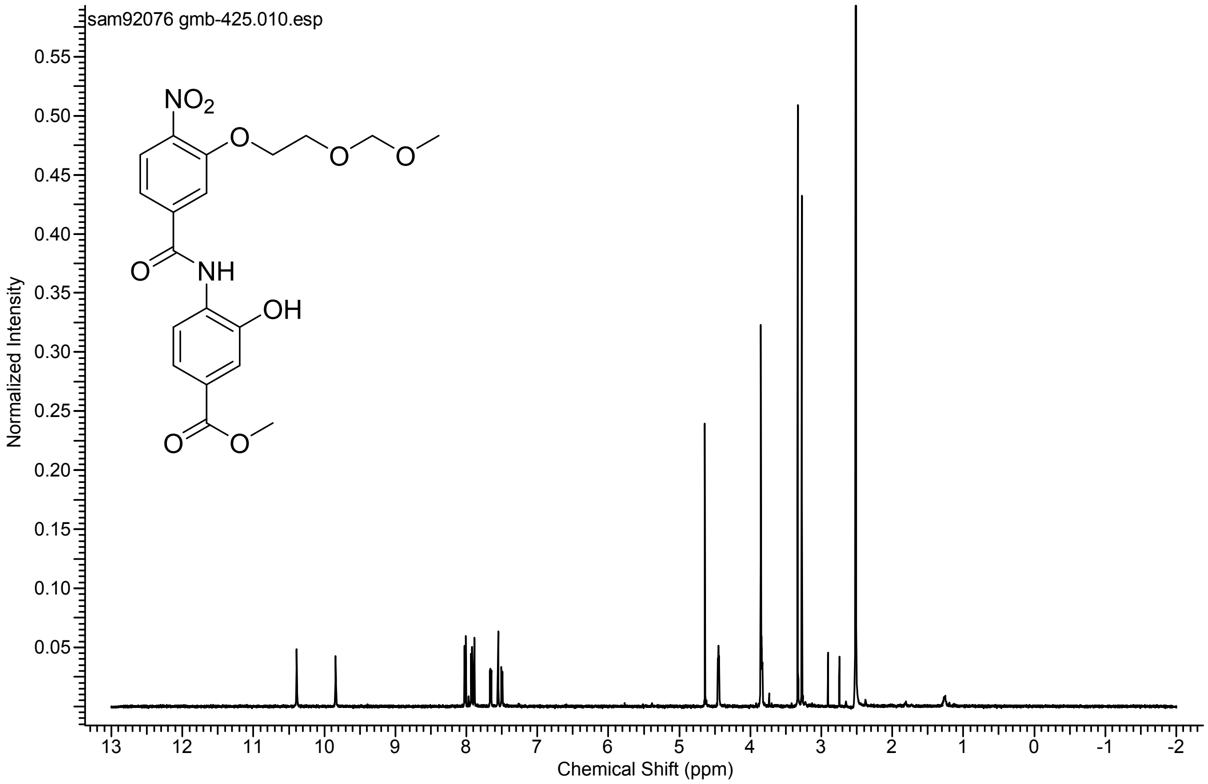

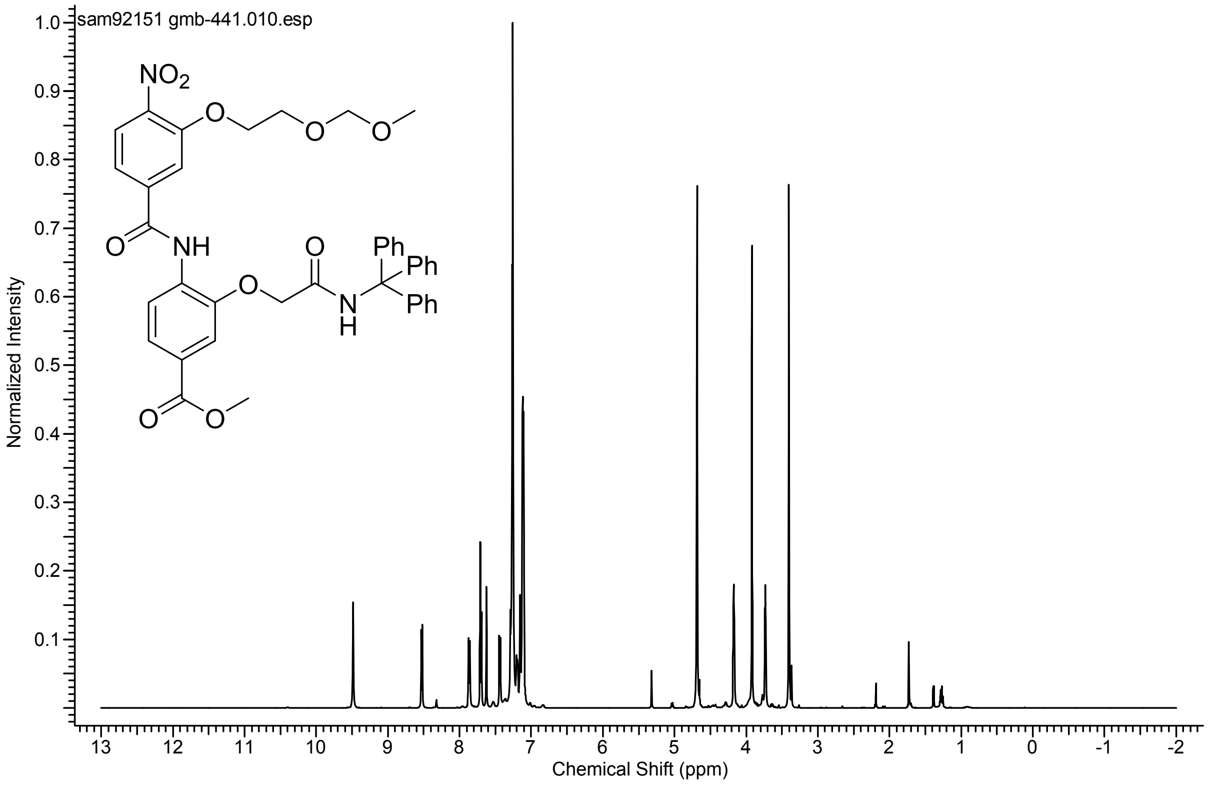

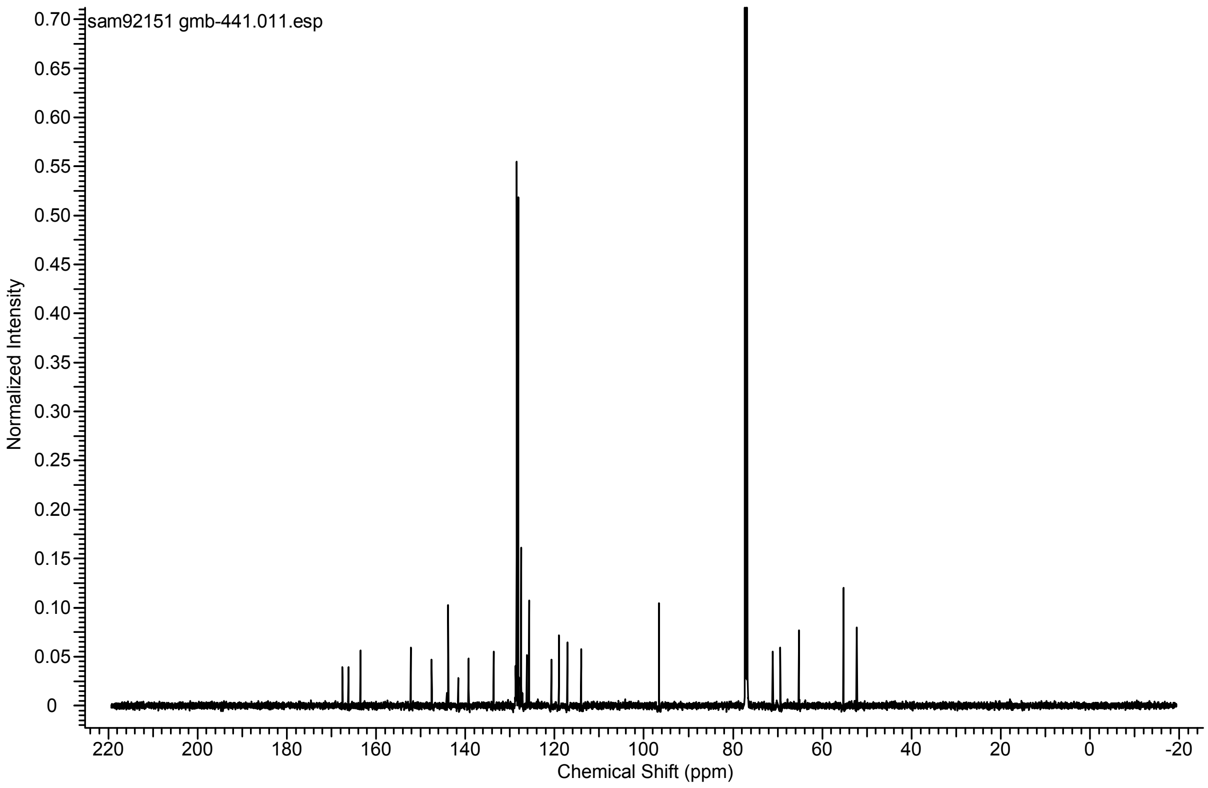
sam92151 gmb-441.011.esp
Normalized Intensity
Chemical Shift (ppm)

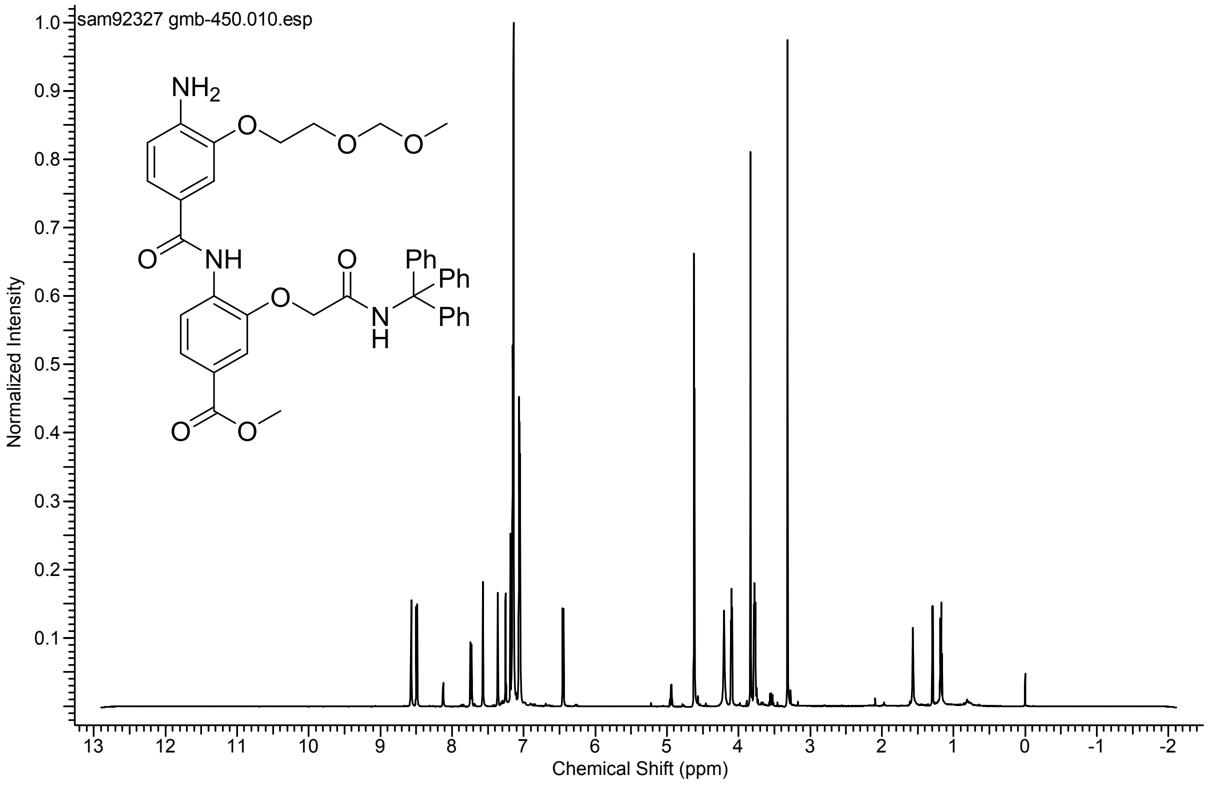
sam92327 gmb-450.010.esp
Normalized Intensity
Chemical Shift (ppm)
NH2
O
O
O
O
NH
O
Ph
Ph
Ph
N
H
O
O

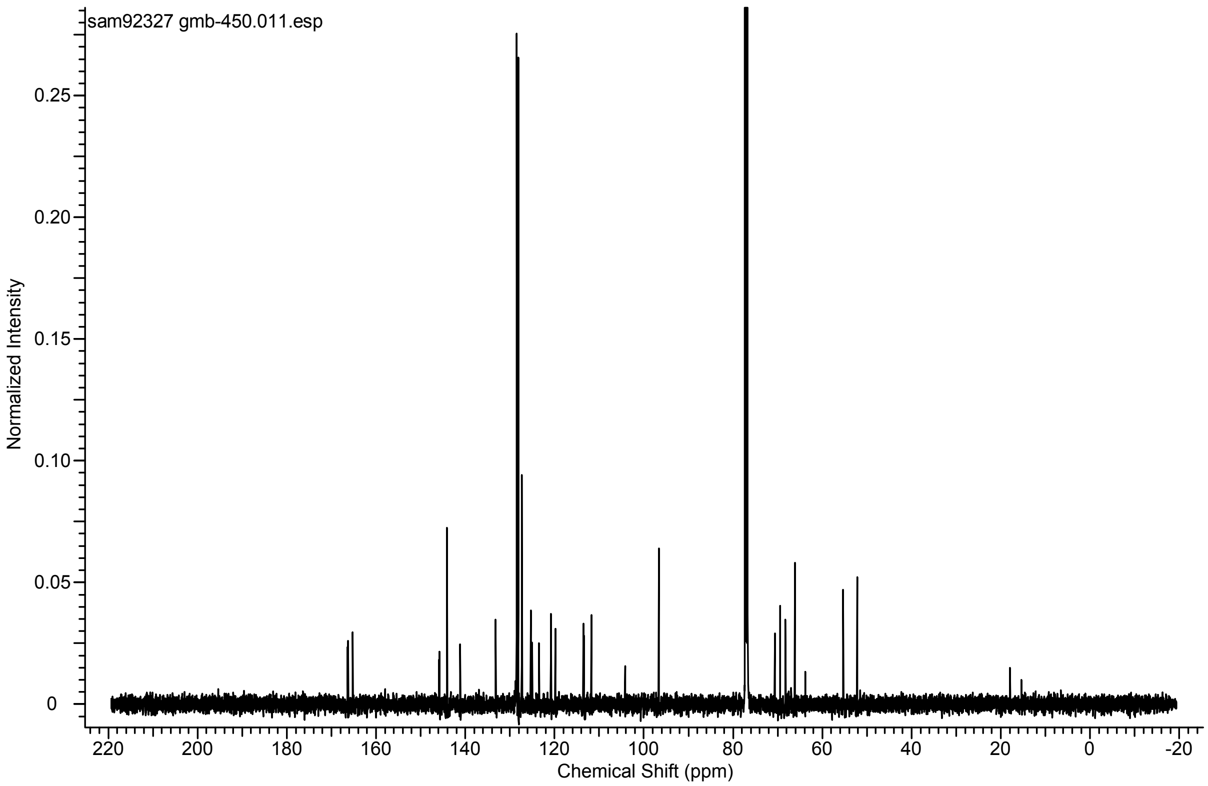
sam92327 gmb-450.011.esp
Normalized Intensity
Chemical Shift (ppm)

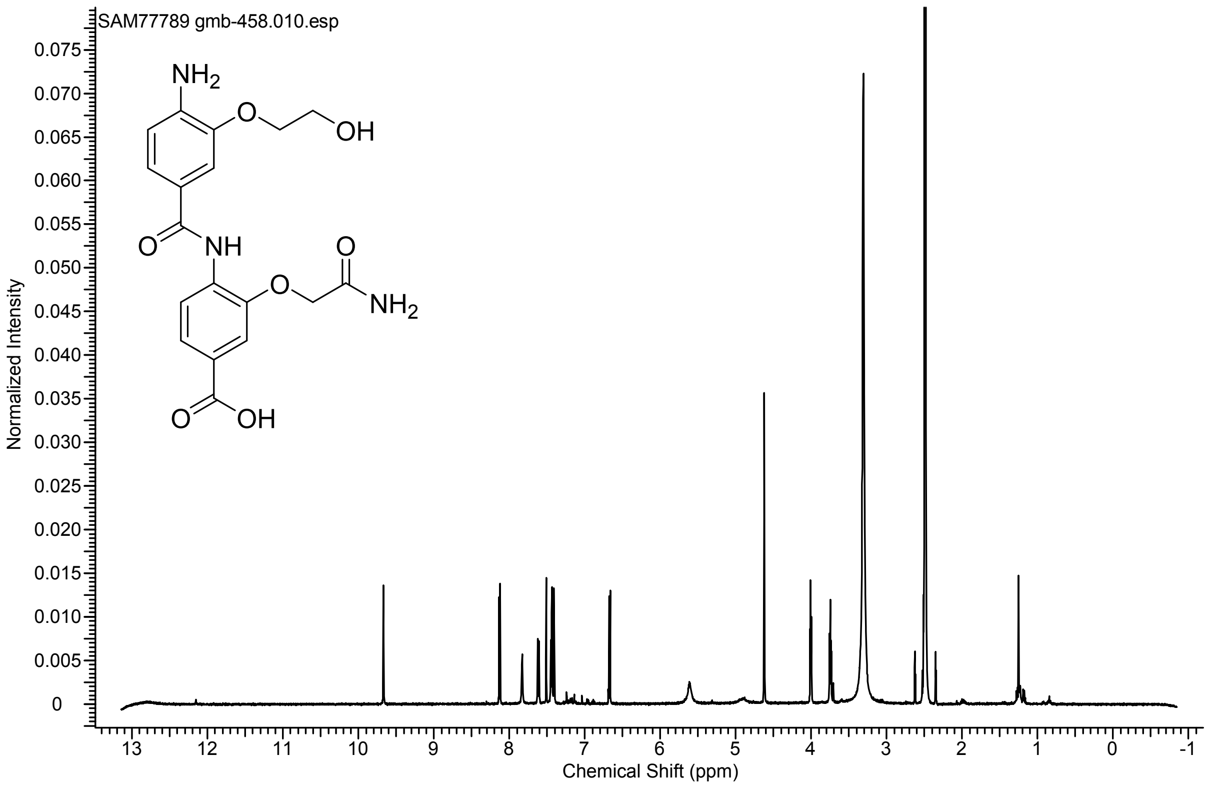
SAM77789 gmb-458.010.esp
Normalized Intensity
Chemical Shift (ppm)
NH2
O
OH
O
NH
O
NH2
O
OH

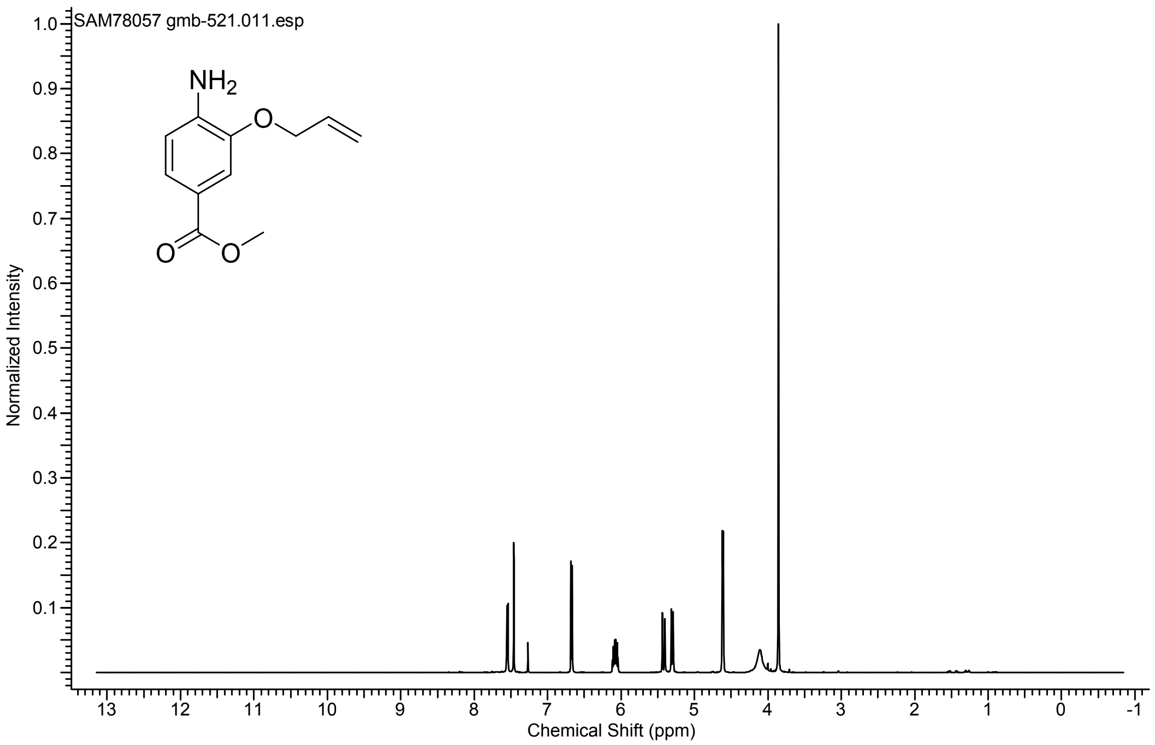
SAM78057 gmb-521.011.esp
NH2
O
O
O
Normalized Intensity
Chemical Shift (ppm)

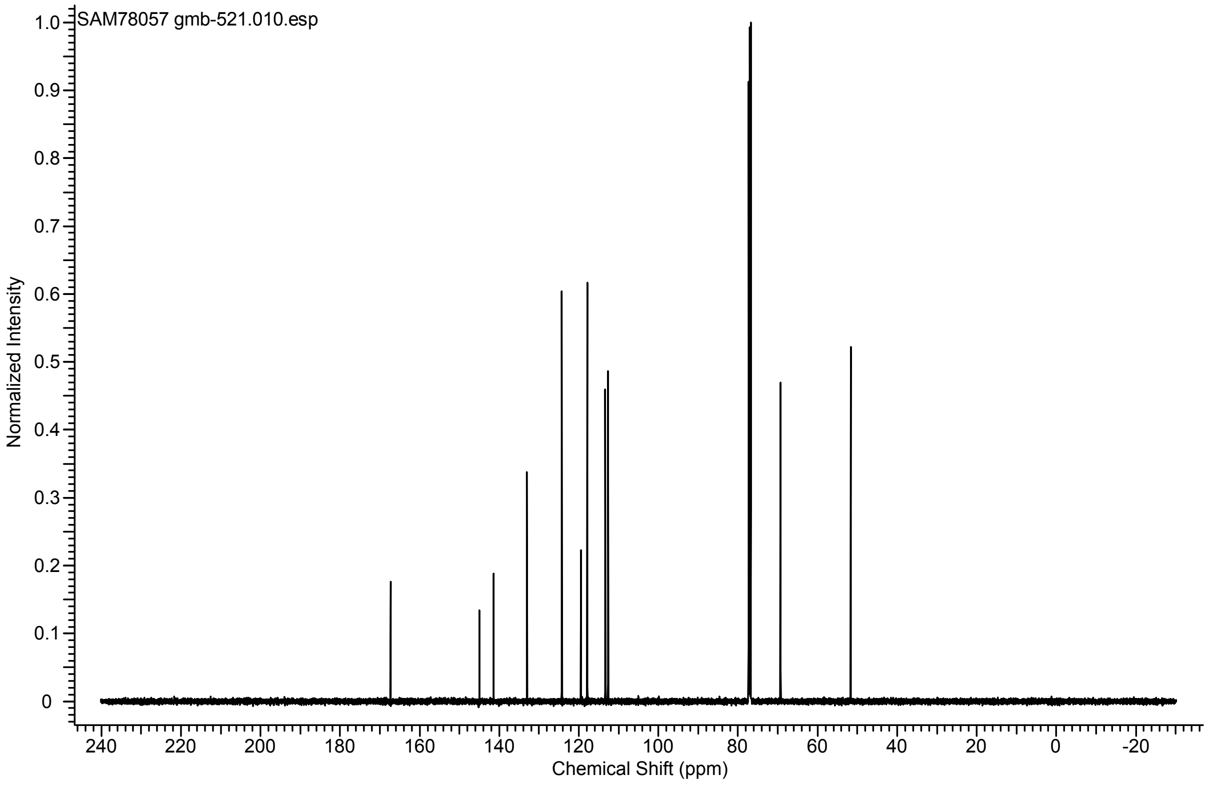
SAM78057 gmb-521.010.esp
Normalized Intensity
Chemical Shift (ppm)

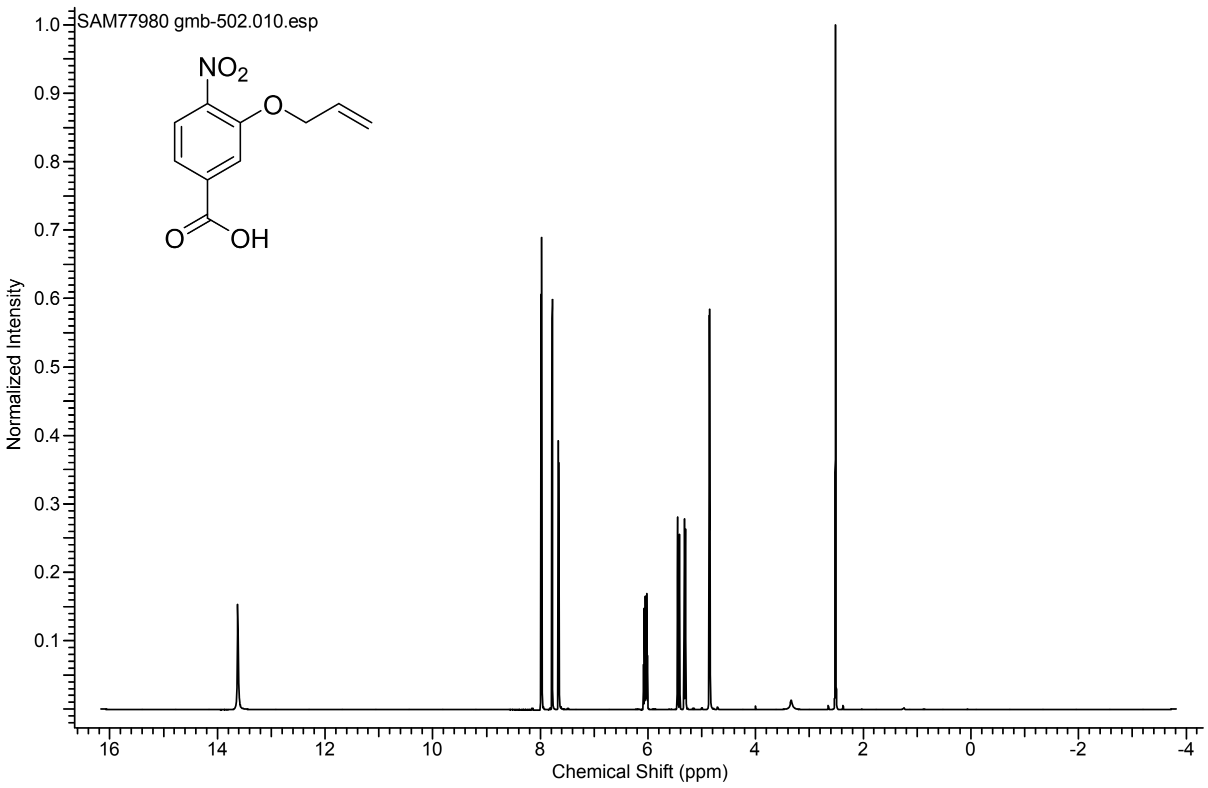
SAM77980 gmb-502.010.esp
NO2
O
O
OH
Normalized Intensity
Chemical Shift (ppm)

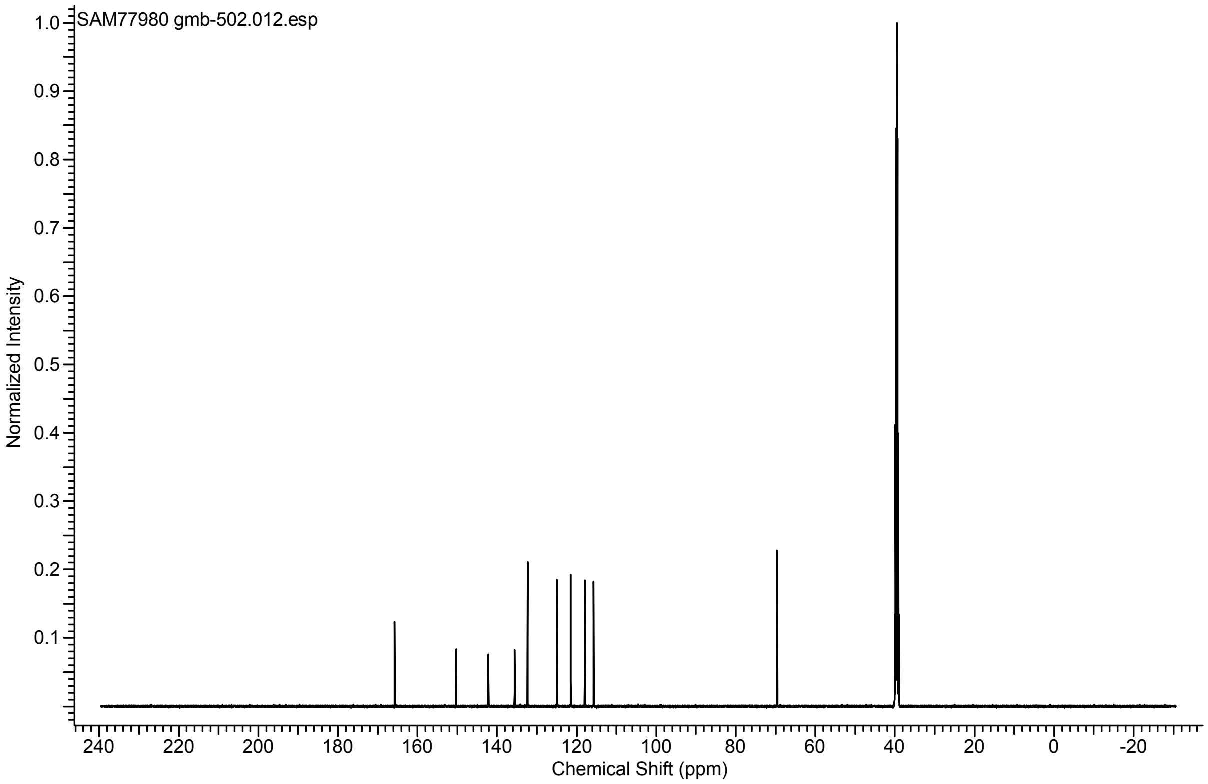
SAM77980 gmb-502.012.esp
Normalized Intensity
Chemical Shift (ppm)

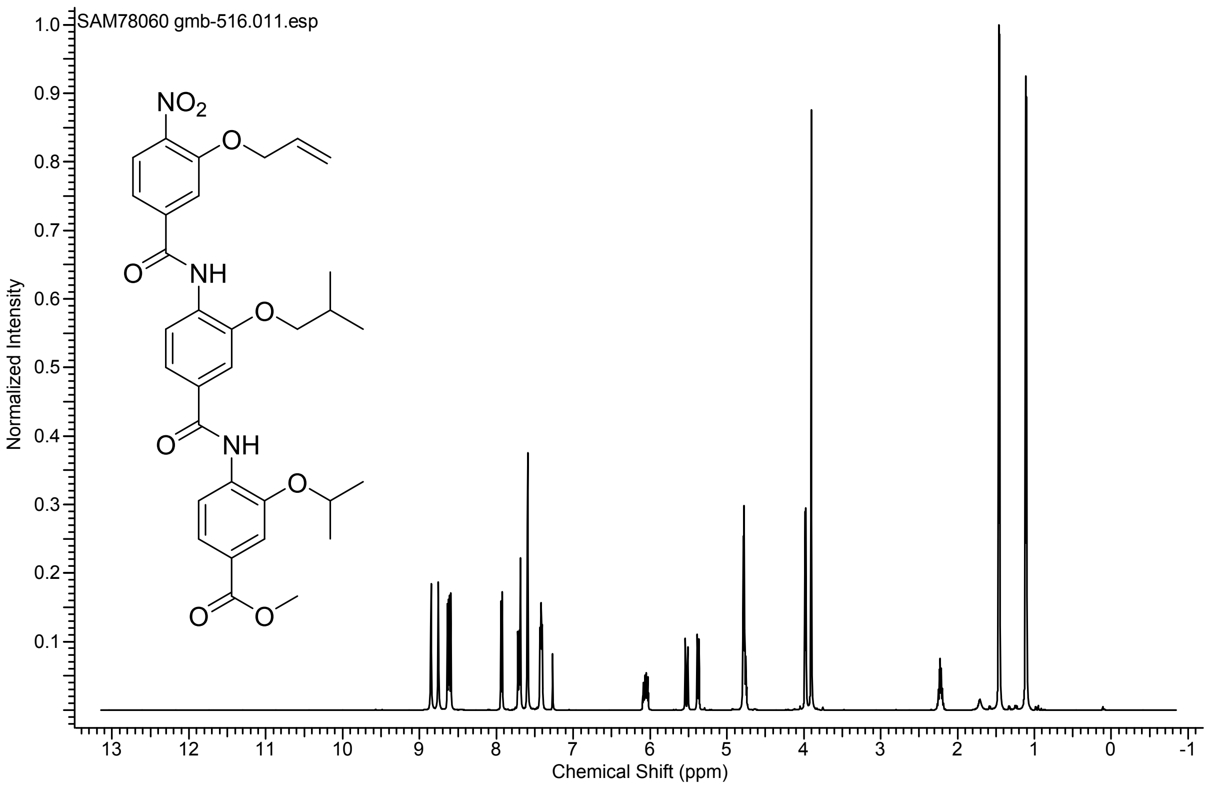
SAM78060 gmb-516.011.esp
NO2
O
O
NH
O
O
NH
O
O
O
Normalized Intensity
Chemical Shift (ppm)

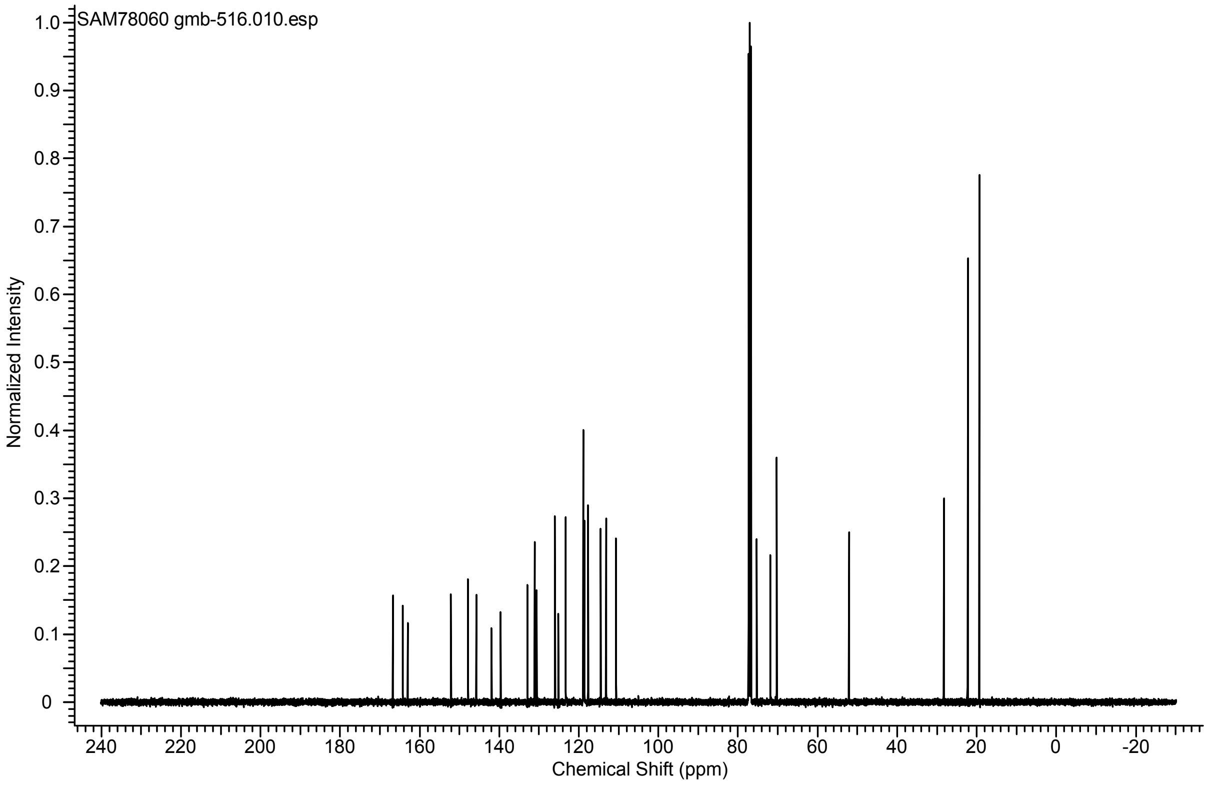
SAM78060 gmb-516.010.esp
Normalized Intensity
Chemical Shift (ppm)

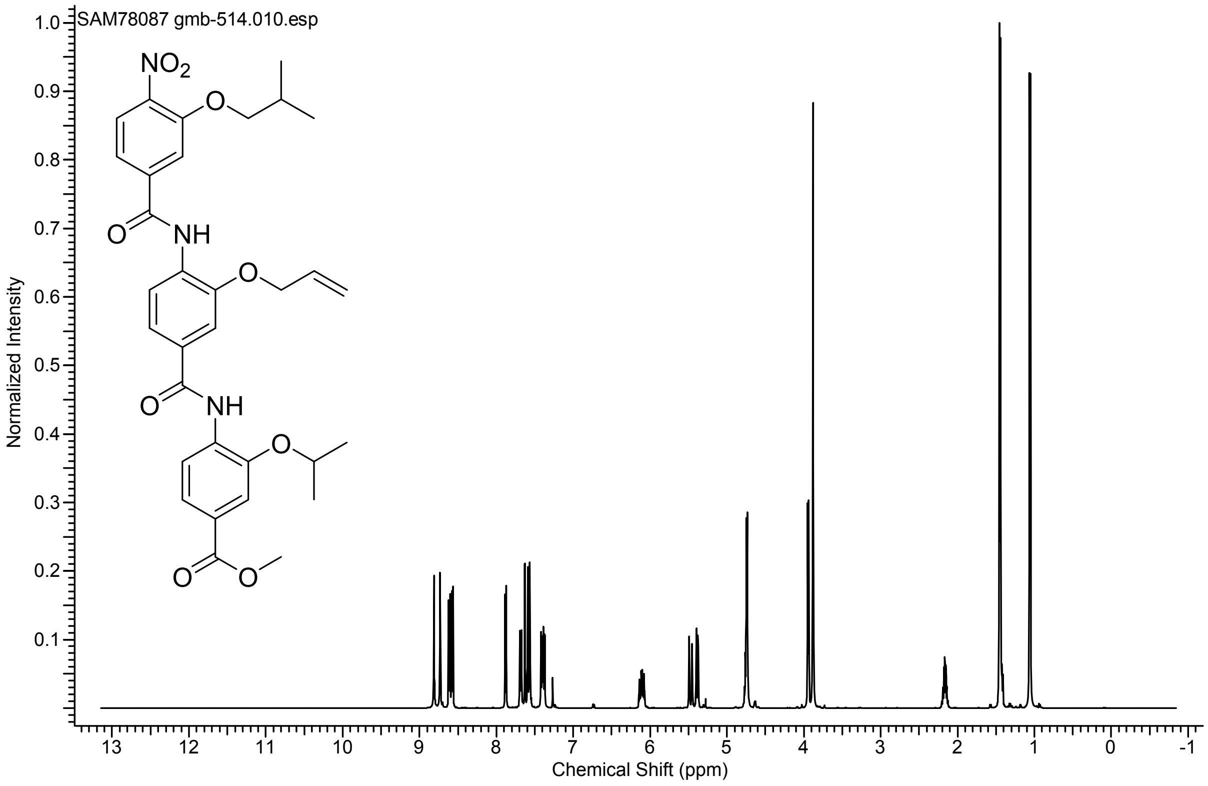
SAM78087 gmb-514.010.esp
Normalized Intensity
Chemical Shift (ppm)
NO2
O
NH
O
NH
O
O
O
O
O

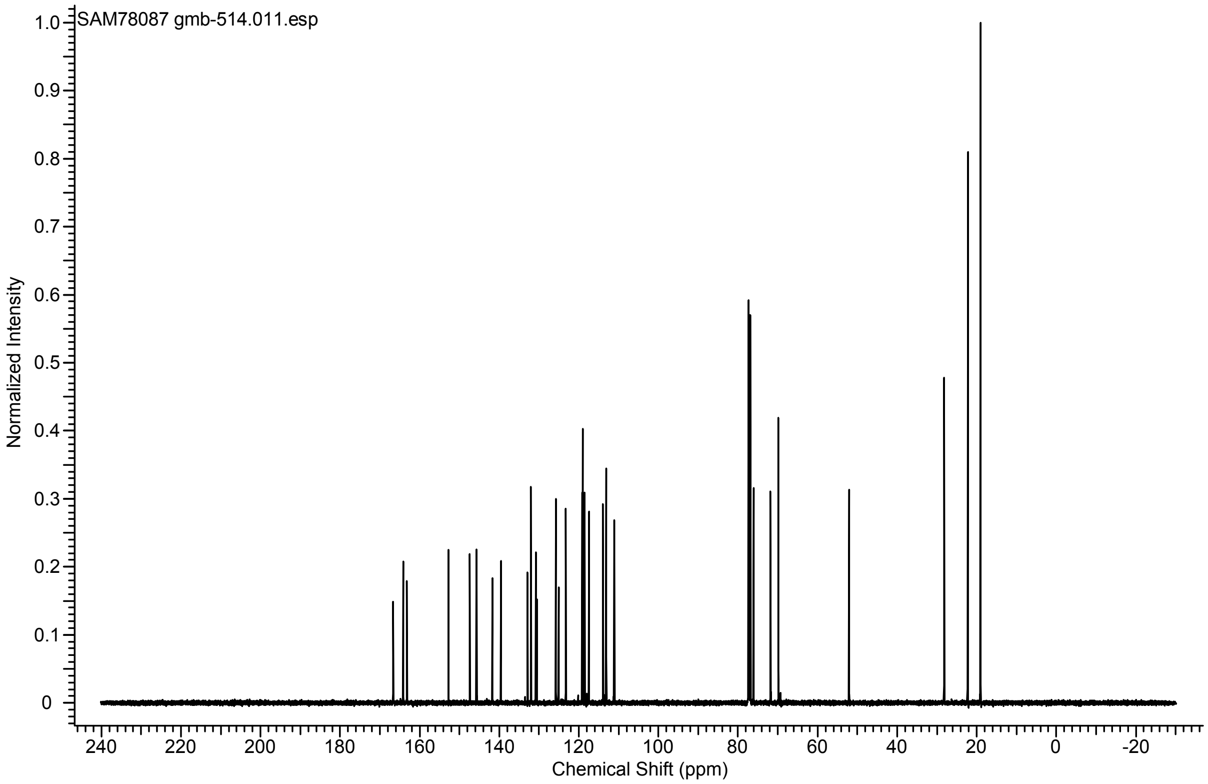
SAM78087 gmb-514.011.esp
Normalized Intensity
Chemical Shift (ppm)

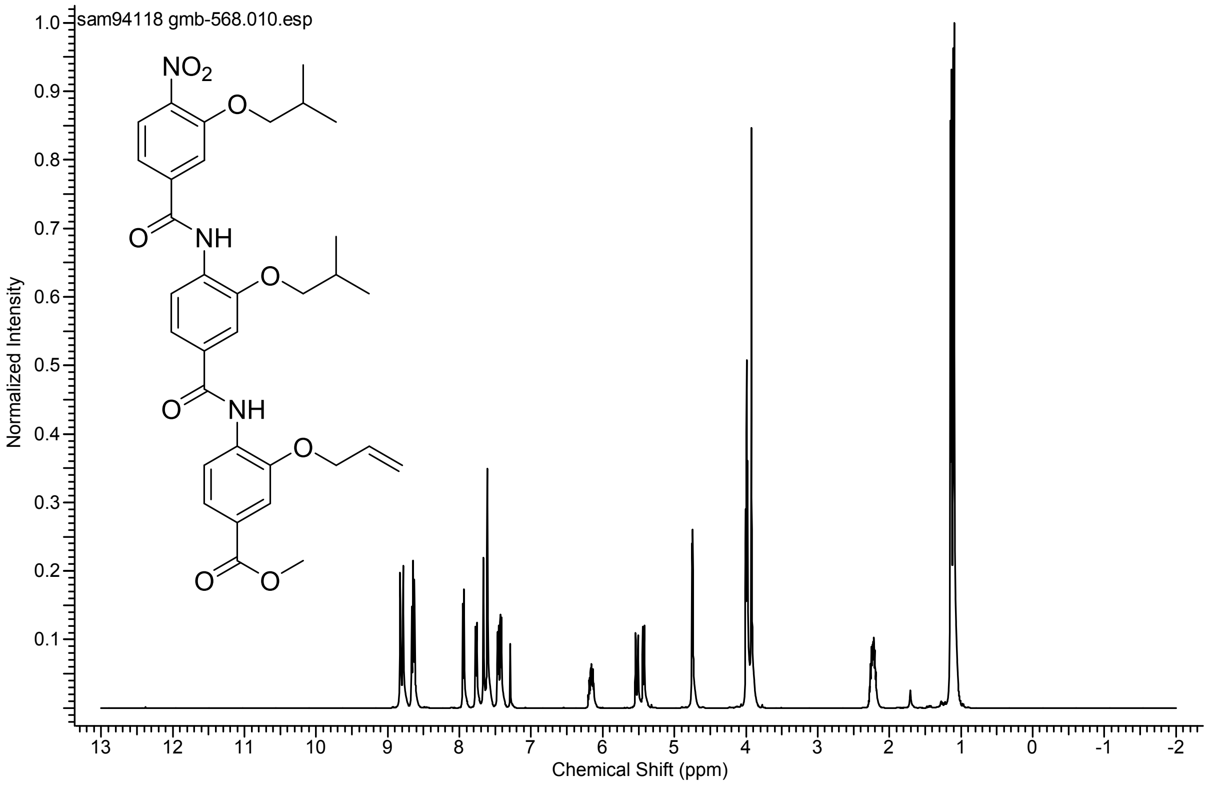
sam94118 gmb-568.010.esp
NO_2
O
O
NH
O
O
NH
O
O
O
Normalized Intensity
Chemical Shift (ppm)

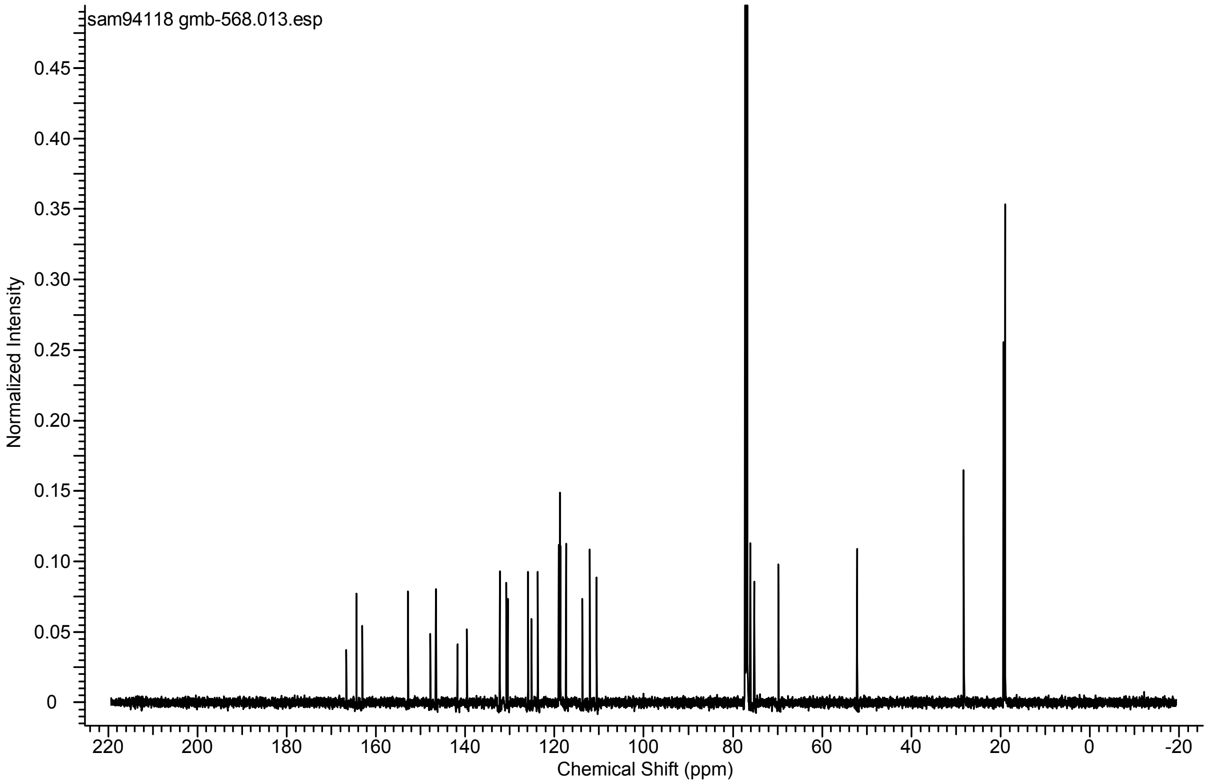
sam94118 gmb-568.013.esp
Normalized Intensity
Chemical Shift (ppm)

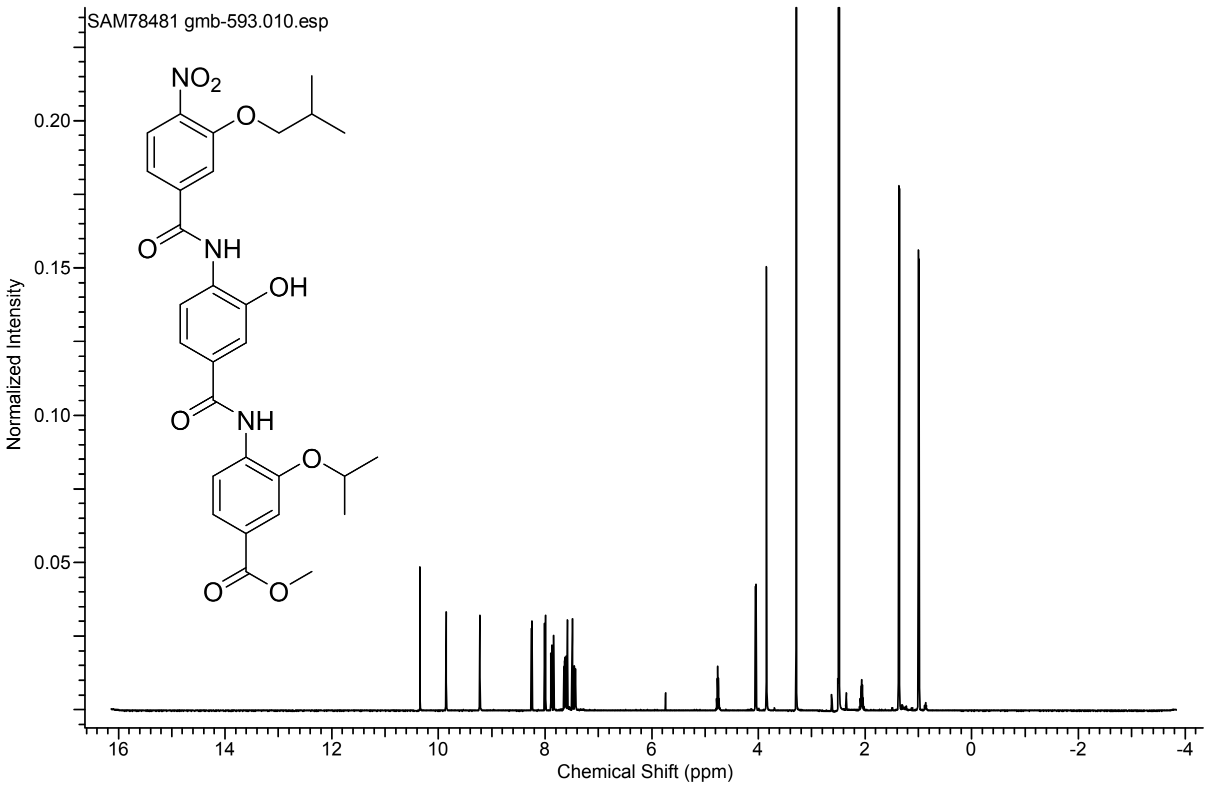
SAM78481 gmb-593.010.esp
NO_2
O
NH
OH
NH
O
Normalized Intensity
Chemical Shift (ppm)
0.20
0.15
0.10
0.05
16
14
12
10
8
6
4
2
0
-2
-4

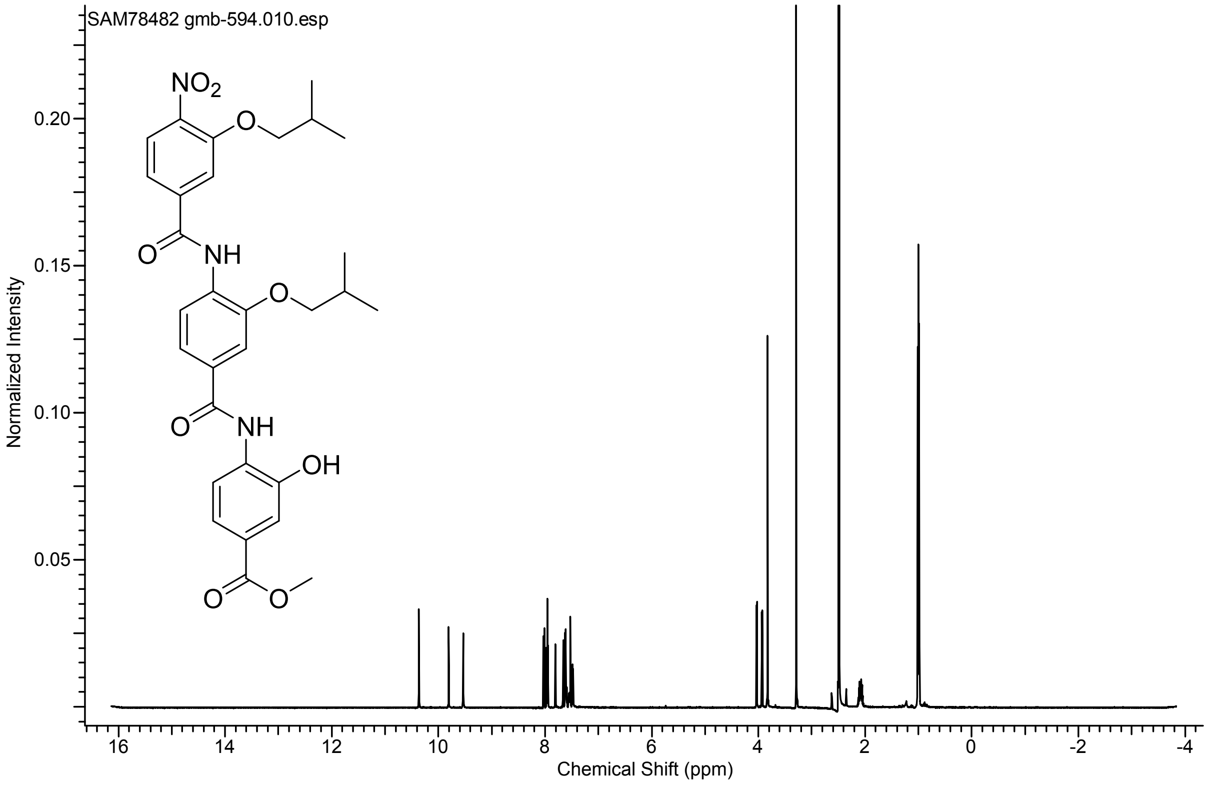
SAM78482 gmb-594.010.esp
NO_2
O
NH
NH
OH
O
Normalized Intensity
Chemical Shift (ppm)
0.20
0.15
0.10
0.05
16
14
12
10
8
6
4
2
0
-2
-4

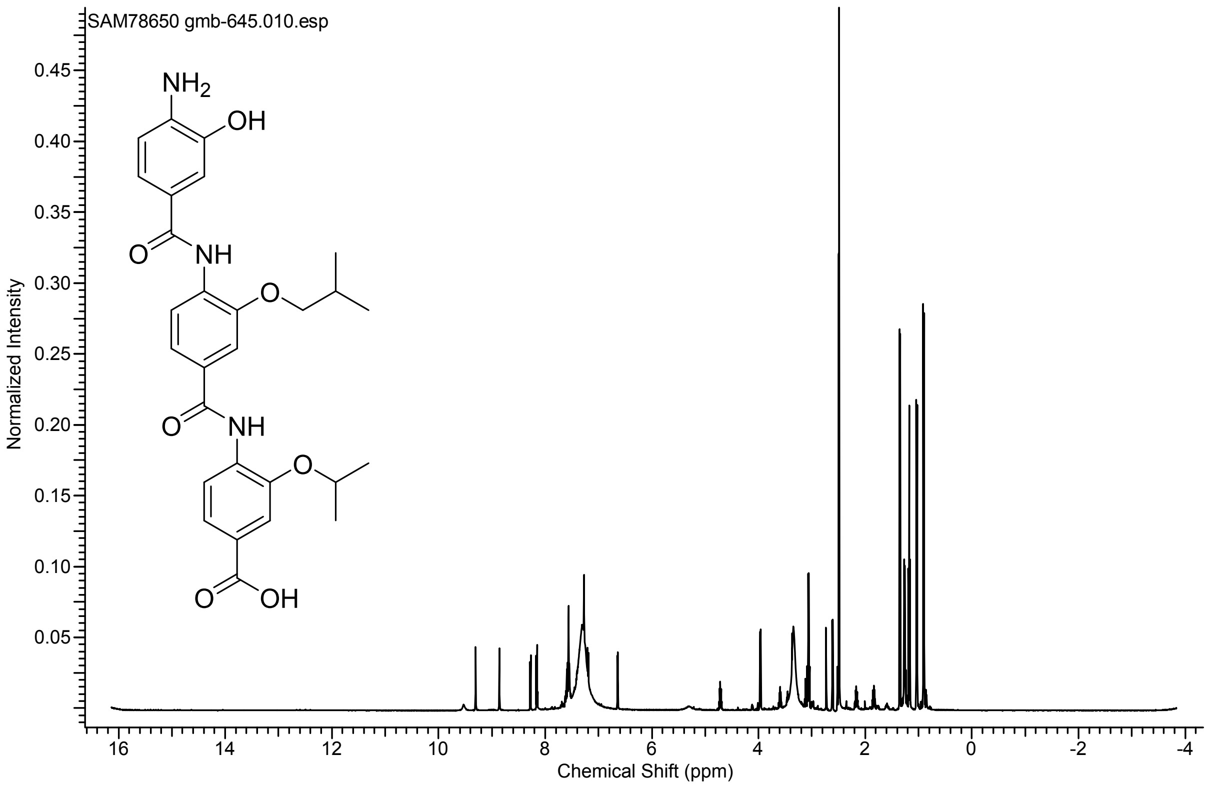
SAM78650 gmb-645.010.esp
Normalized Intensity
Chemical Shift (ppm)
NH2
OH
O
NH
O
O
NH
O
O
OH

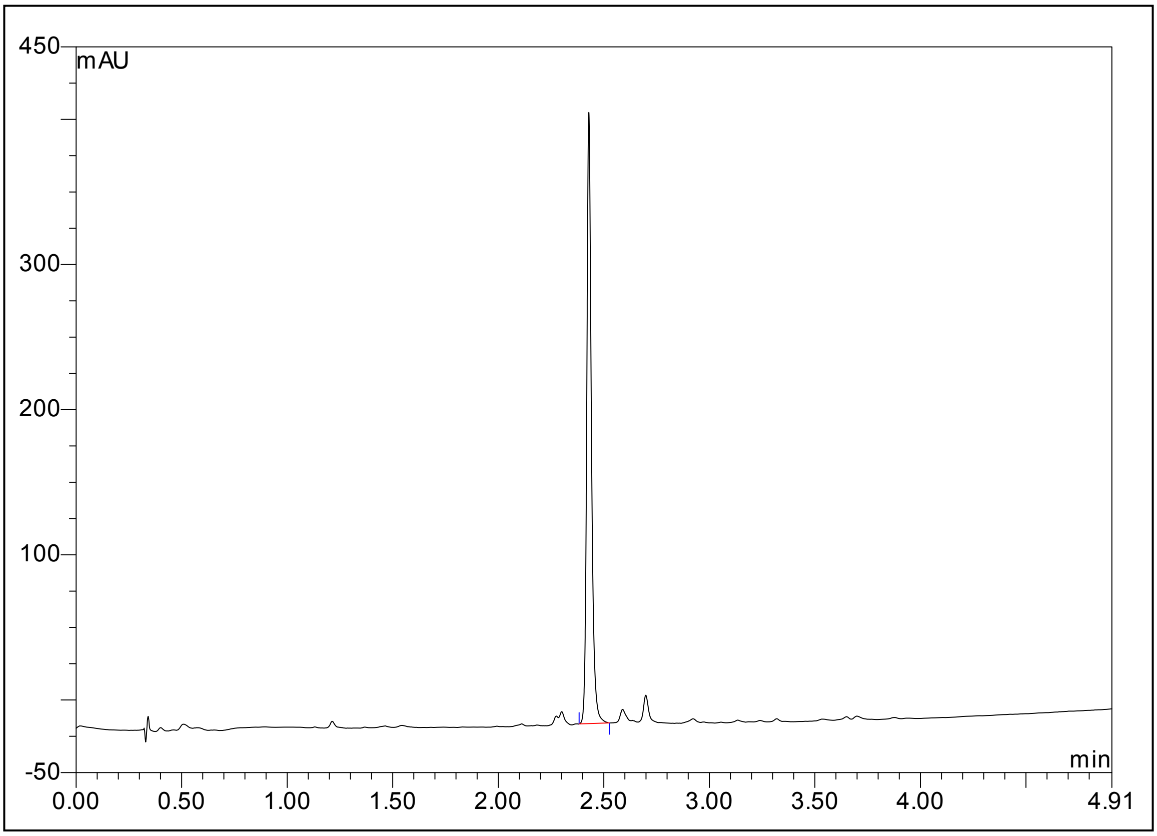
mAU
450
300
200
100
-50
min
0.00
0.50
1.00
1.50
2.00
2.50
3.00
3.50
4.00
4.91

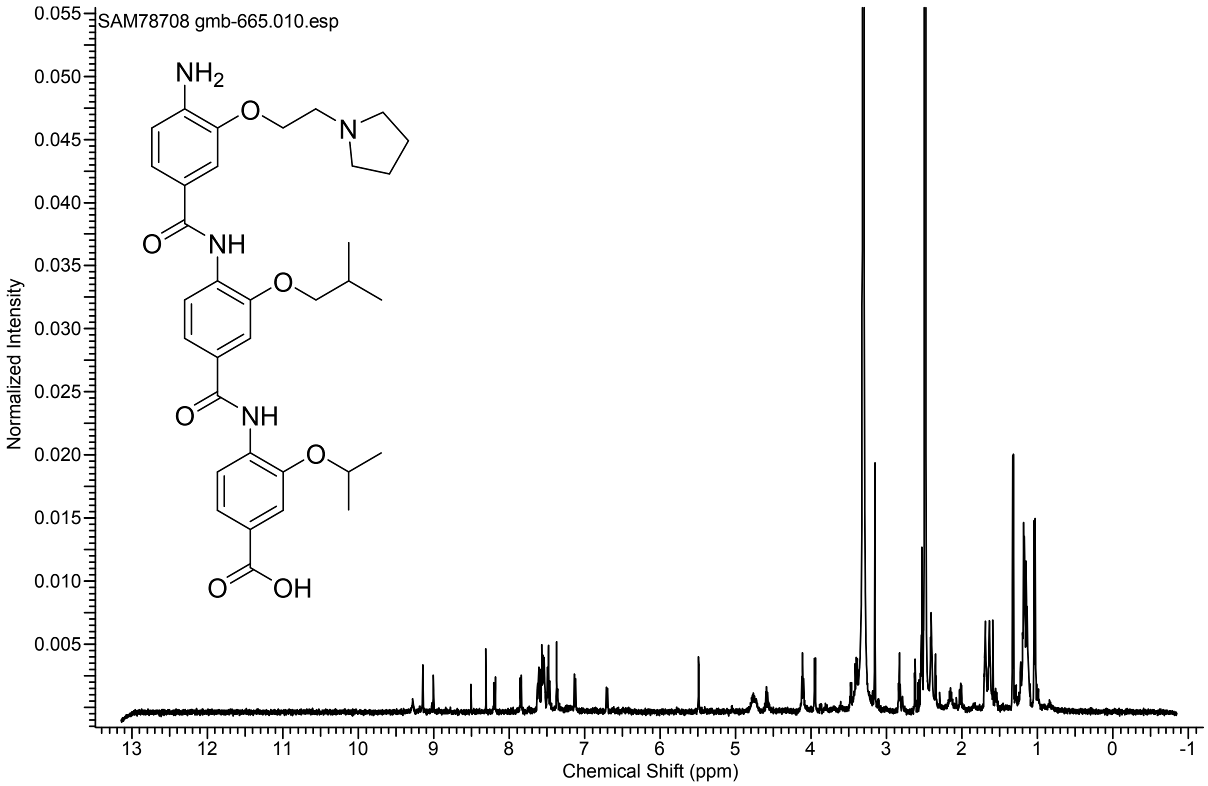
SAM78708 gmb-665.010.esp
Normalized Intensity
Chemical Shift (ppm)
NH2
O
N
NH
OH

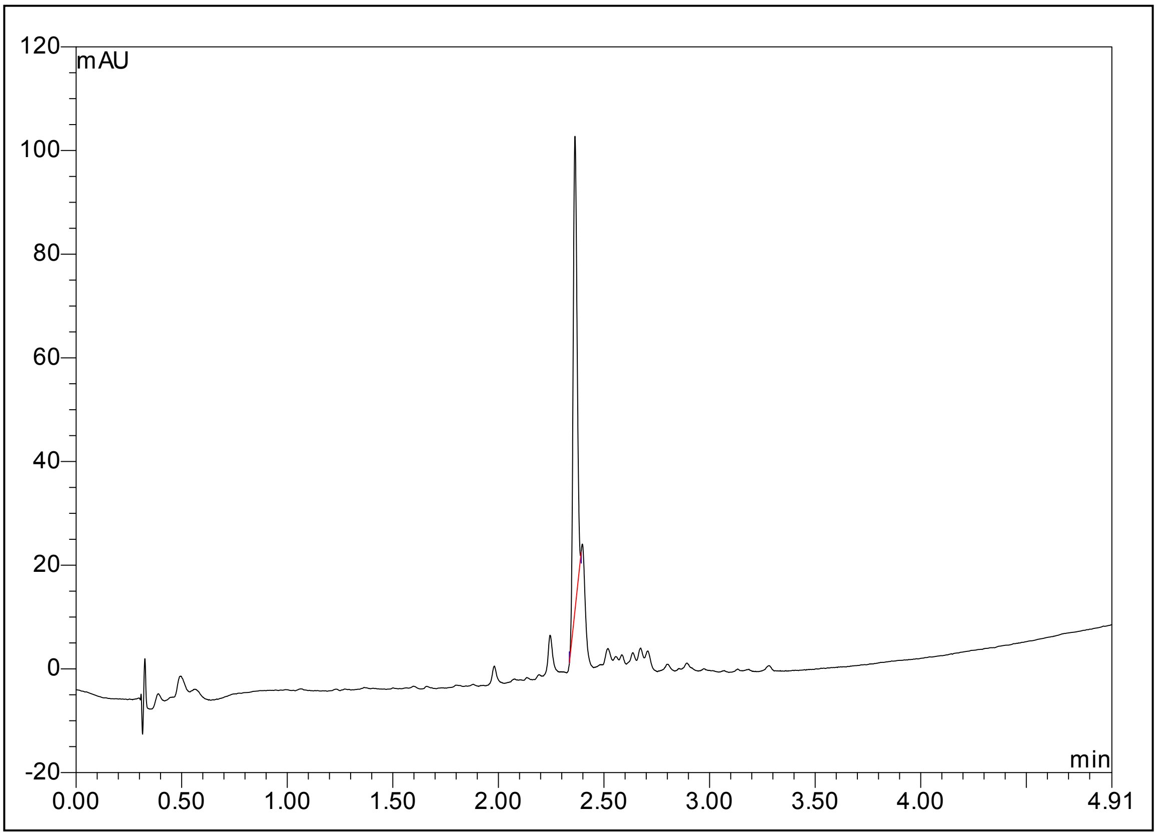
mAU
min

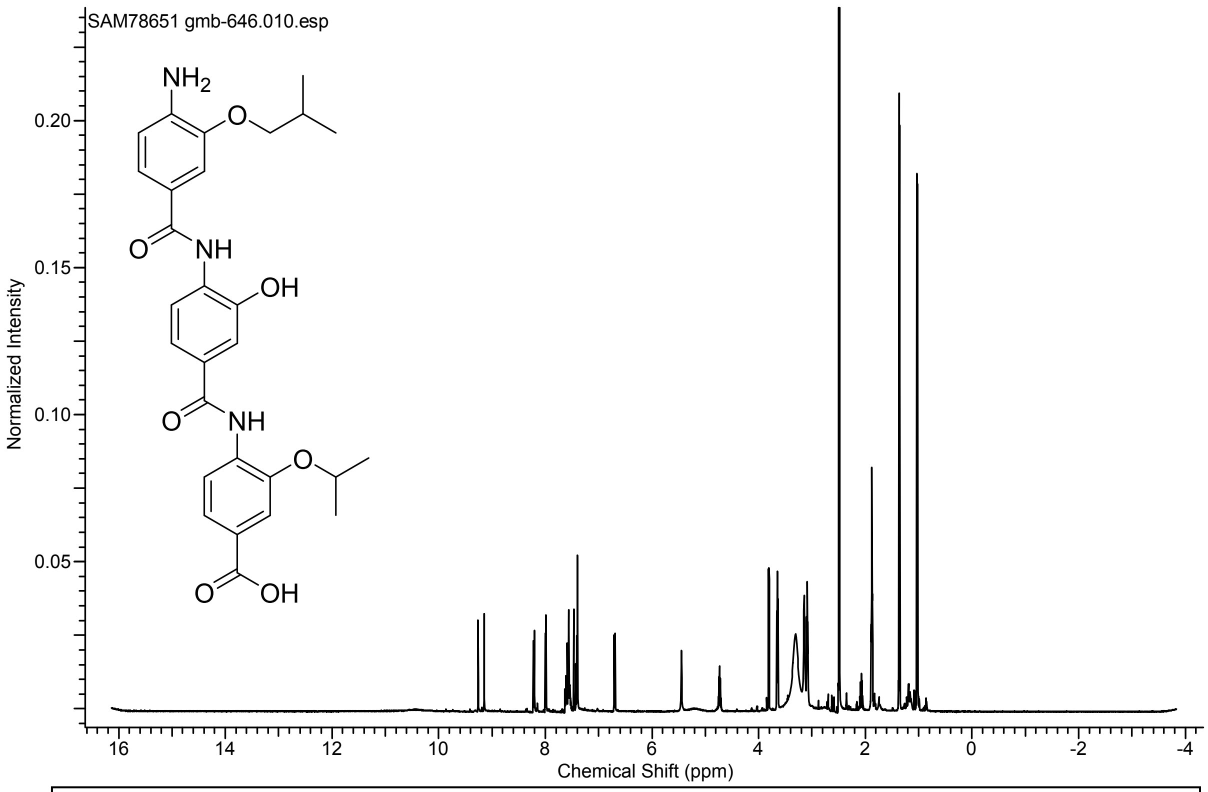
SAM78651 gmb-646.010.esp
Normalized Intensity
Chemical Shift (ppm)
NH2
O
NH
OH
NH
O
OH

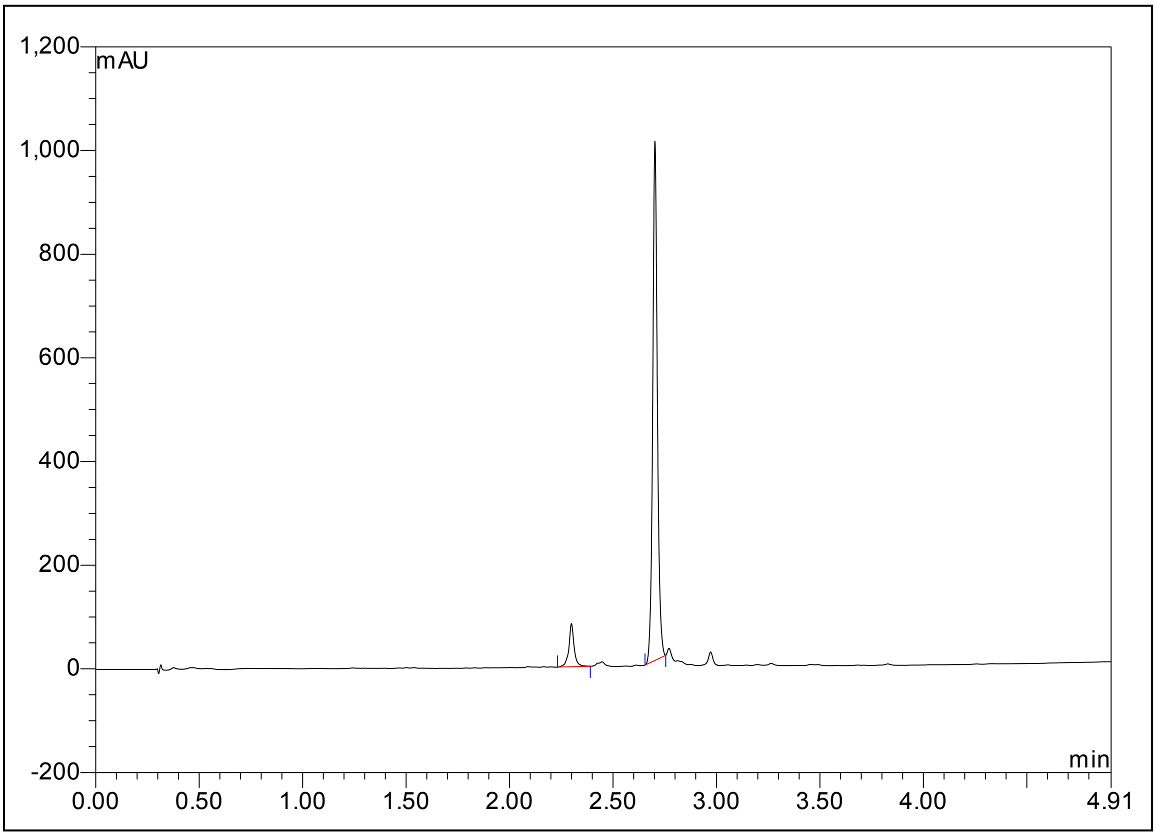
mAU
min

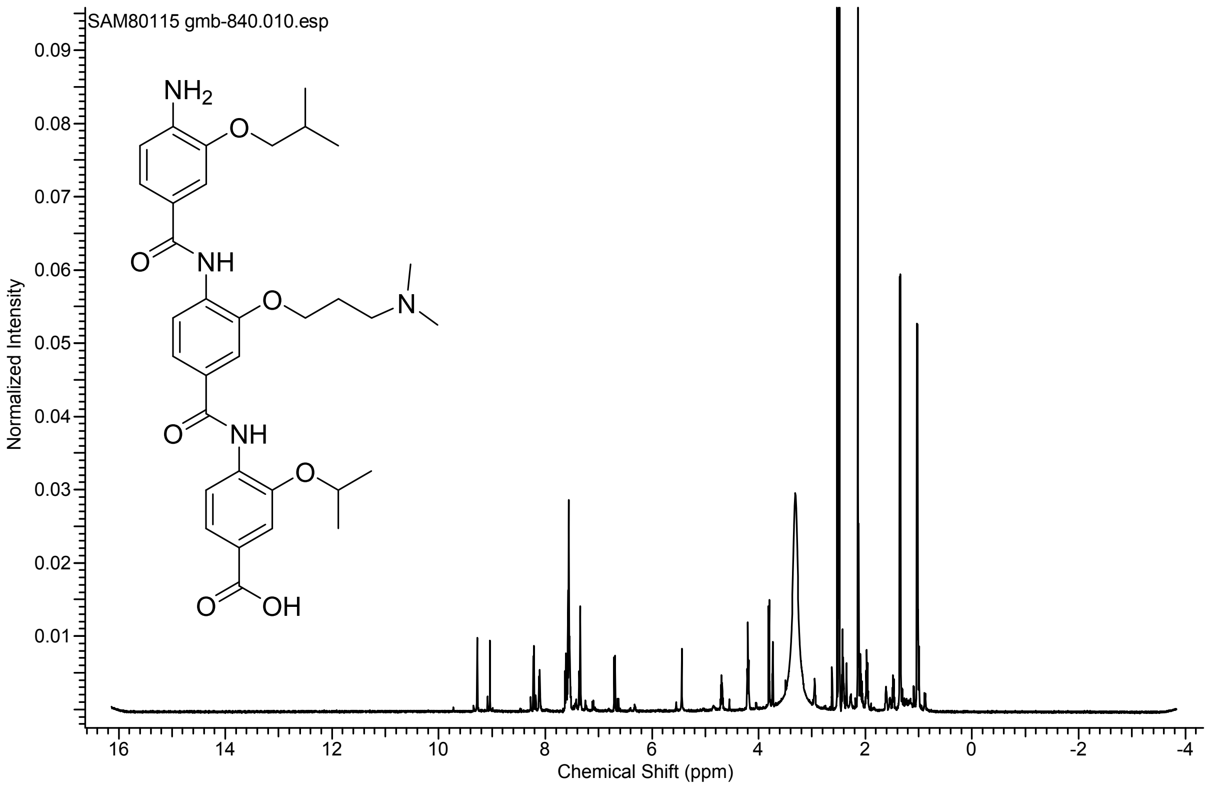

SAM80115 gmb-840.010.esp
Normalized Intensity
Chemical Shift (ppm)
NH2
O
NH
N
OH

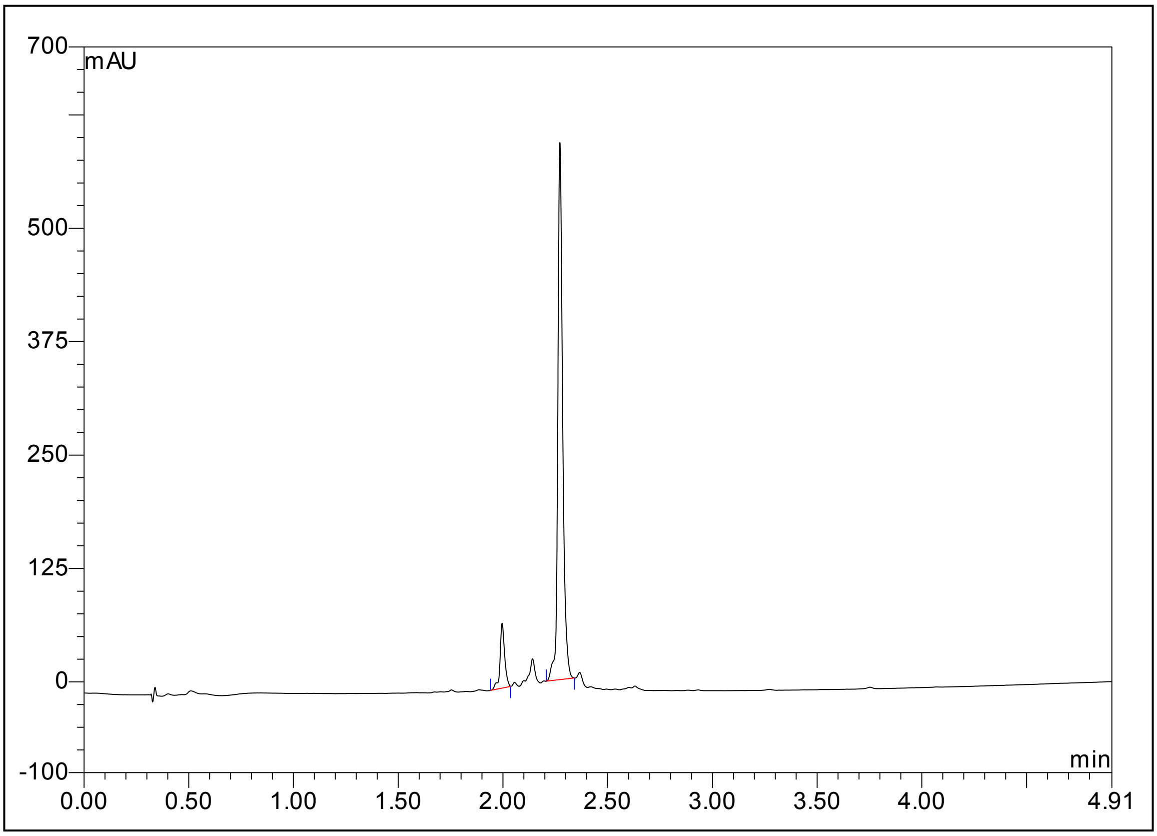

mAU
min

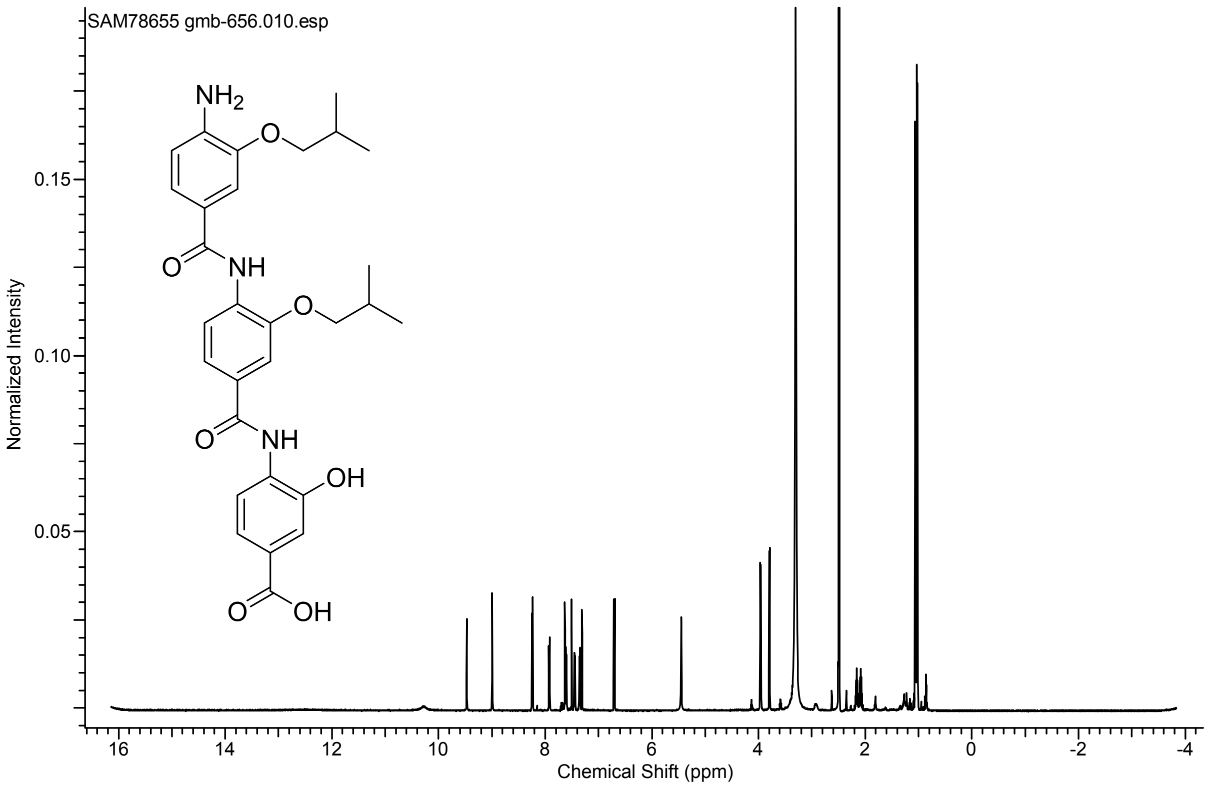
SAM78655 gmb-656.010.esp
NH2
O
O
NH
O
O
NH
OH
O
OH
Normalized Intensity
0.15
0.10
0.05
16
14
12
10
8
6
4
2
0
-2
-4
Chemical Shift (ppm)

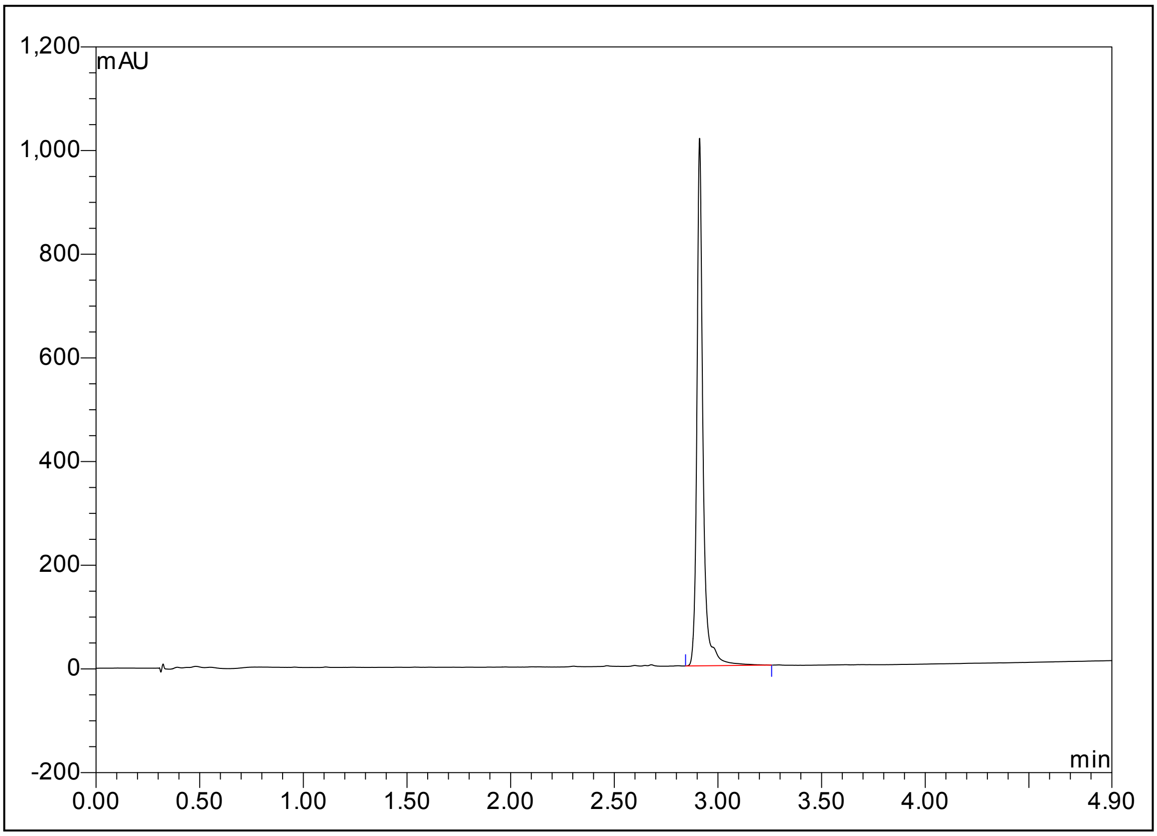
1,200
mAU
1,000
800
600
400
200
0
-200
min
0.00
0.50
1.00
1.50
2.00
2.50
3.00
3.50
4.00
4.90

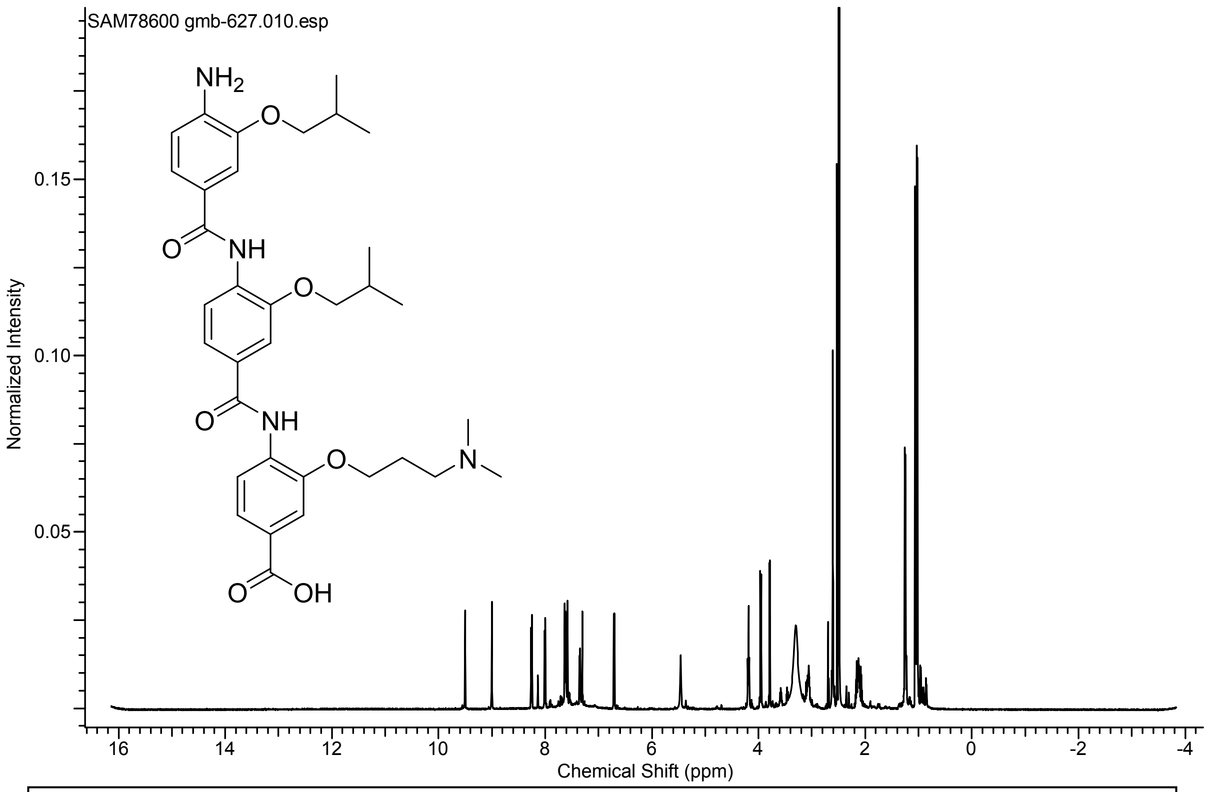
SAM78600 gmb-627.010.esp
Normalized Intensity
Chemical Shift (ppm)
NH2
O
NH
OH
N

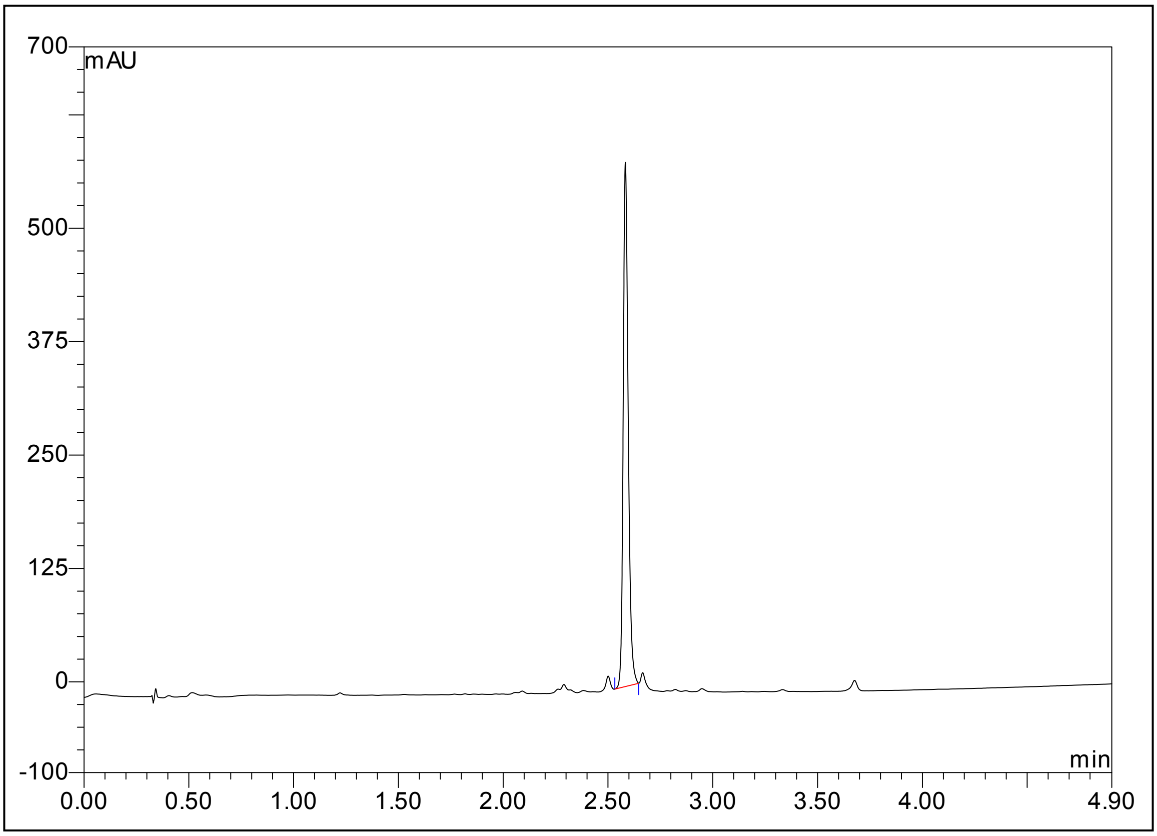
mAU
min

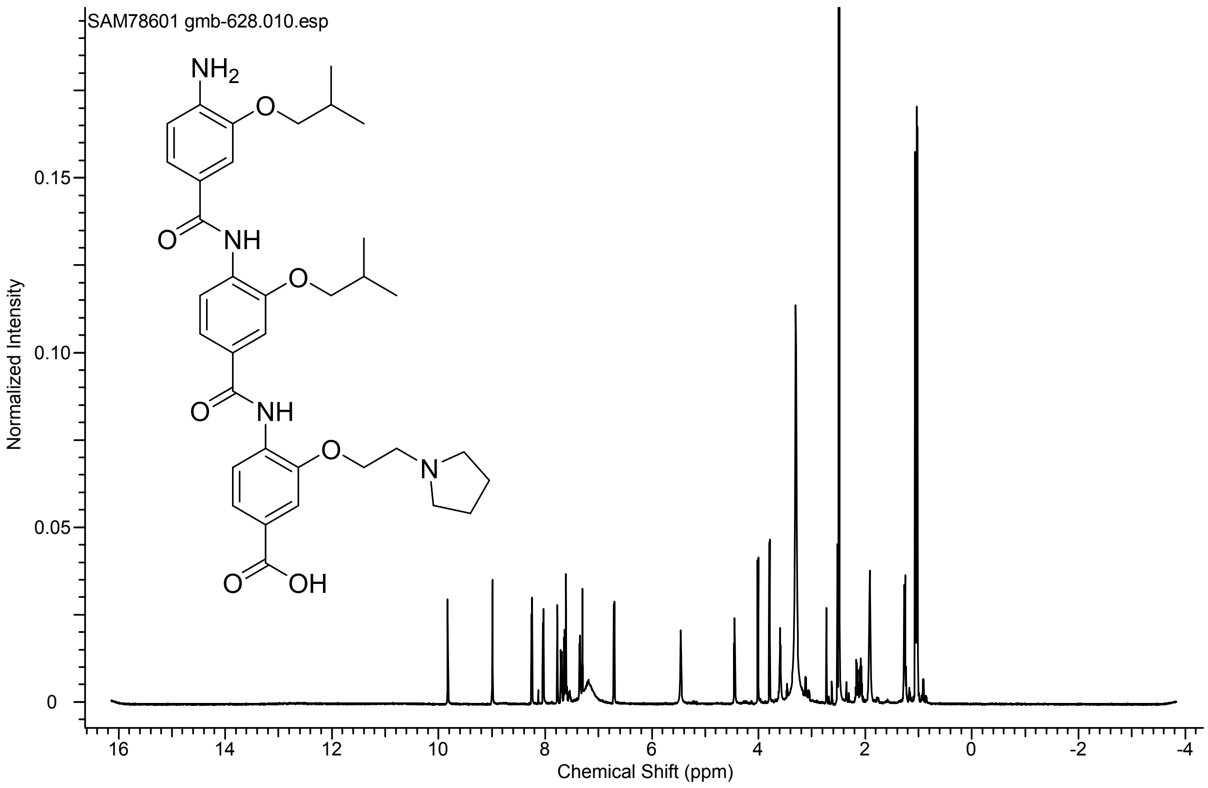
SAM78601 gmb-628.010.esp
Normalized Intensity
Chemical Shift (ppm)
NH2
O
NH
OH
N

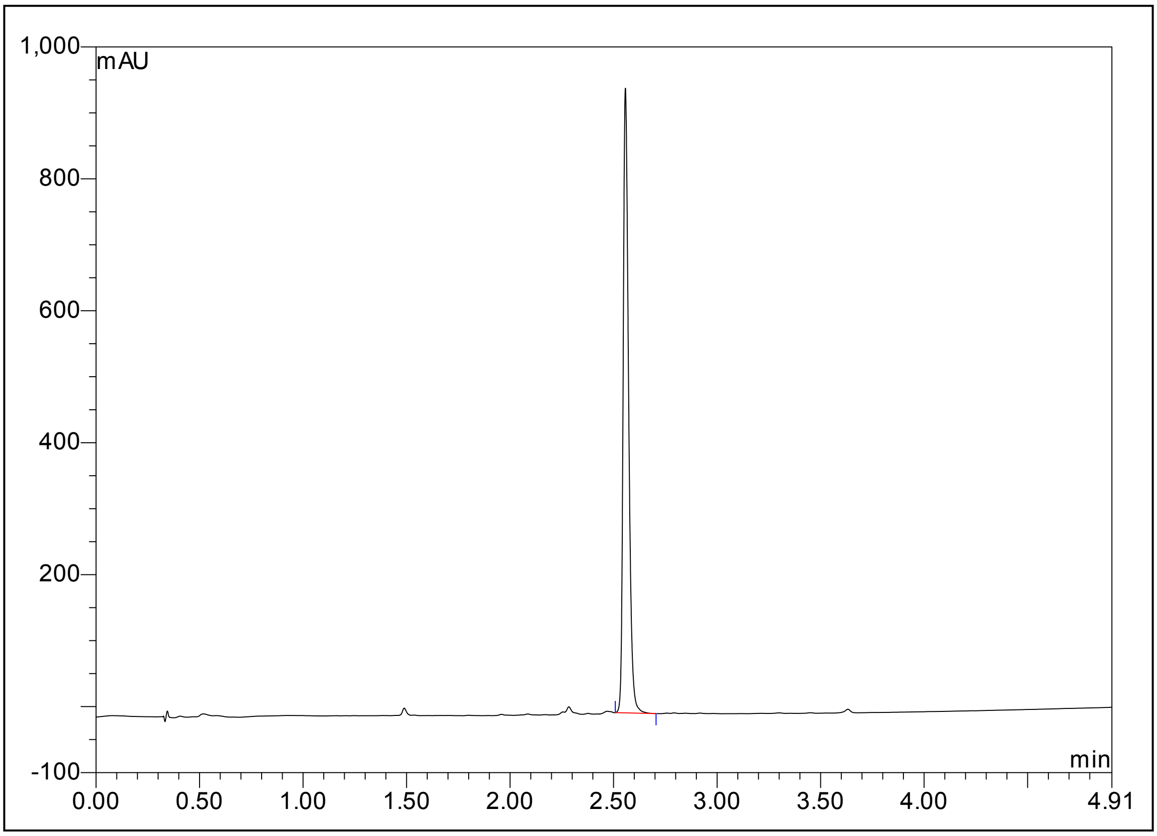
mAU
min

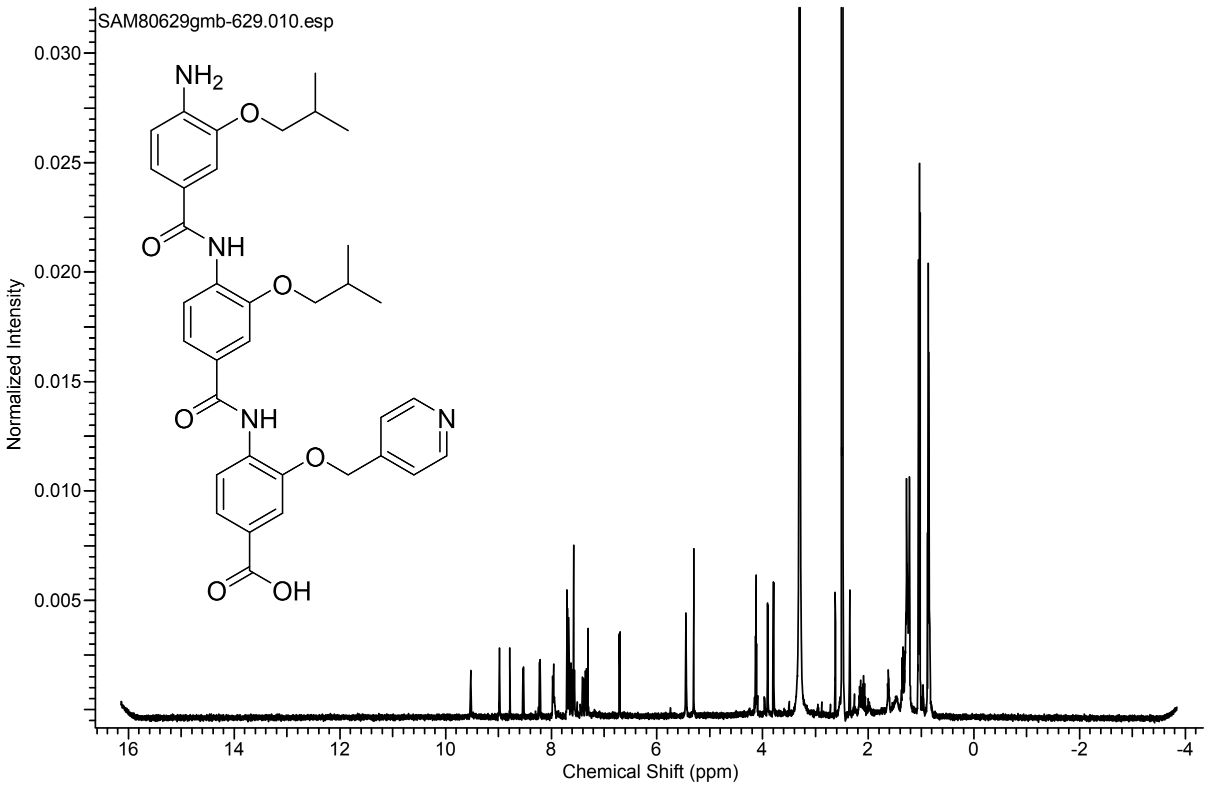
SAM80629gmb-629.010.esp
Normalized Intensity
Chemical Shift (ppm)
NH2
O
NH
OH
N

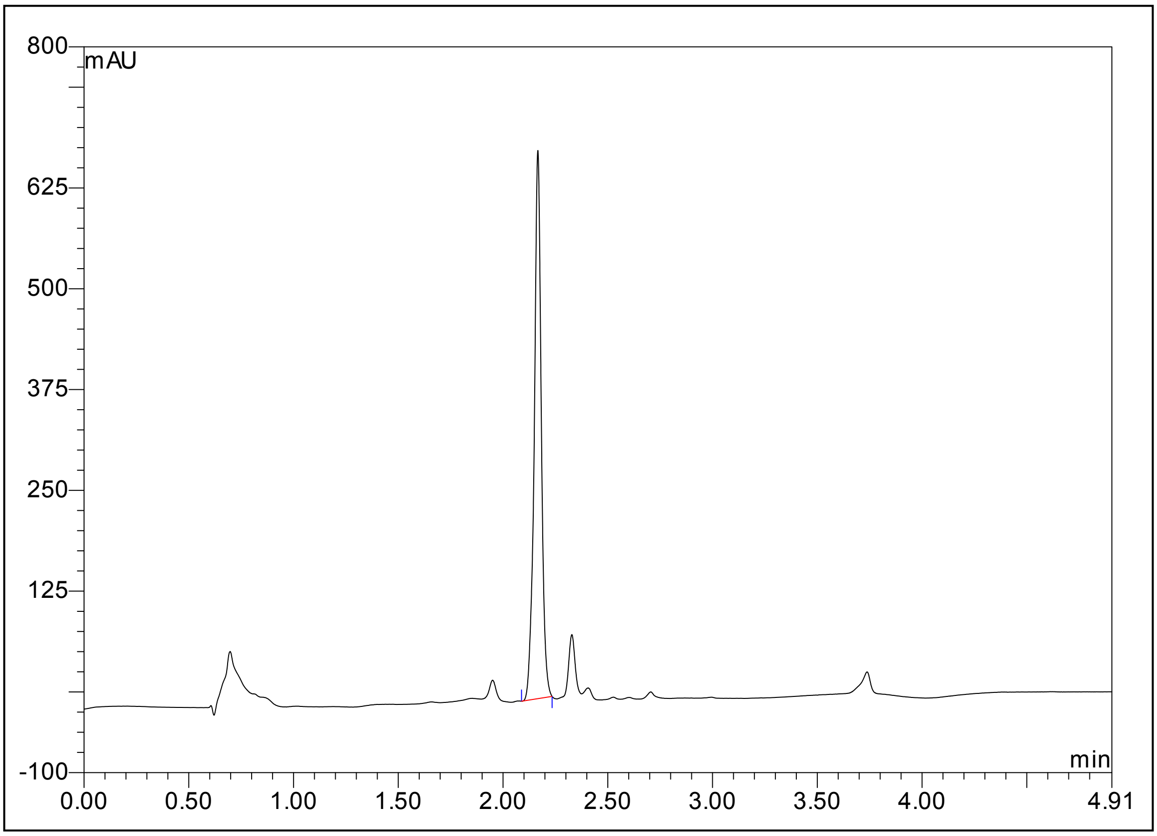
mAU
min

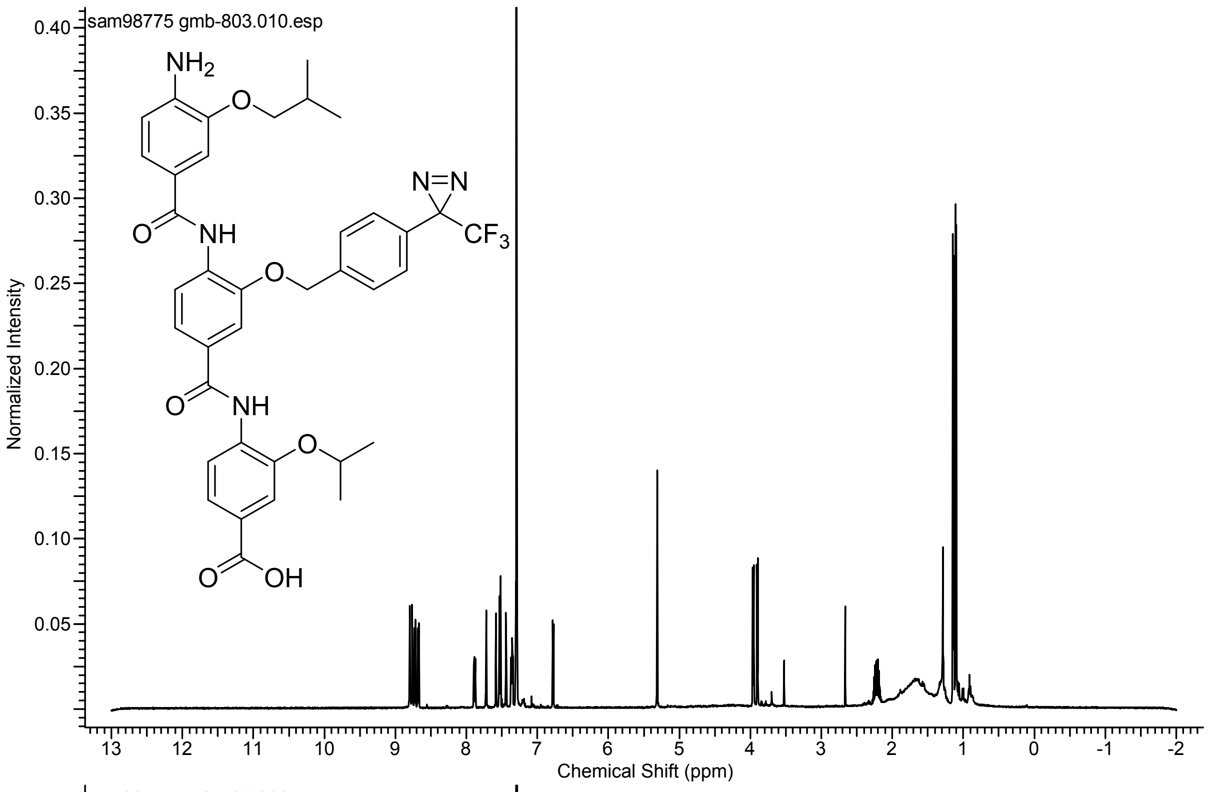
sam98775 gmb-803.010.esp
Normalized Intensity
0.40
0.35
0.30
0.25
0.20
0.15
0.10
0.05
NH2
O
N=N
O
NH
CF3
O
O
NH
O
O
OH
13 12 11 10 9 8 7 6 5 4 3 2 1 0 -1 -2
Chemical Shift (ppm)

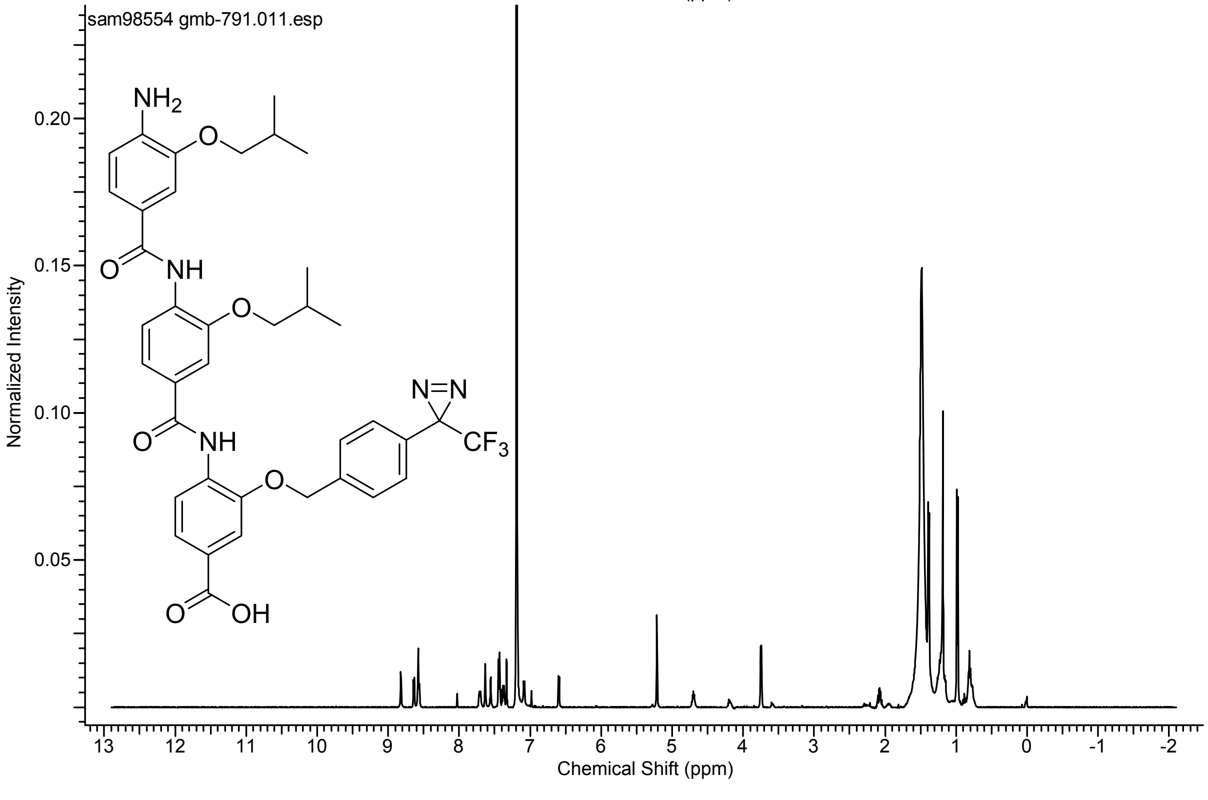
sam98554 gmb-791.011.esp
Normalized Intensity
0.20
0.15
0.10
0.05
NH2
O
O
NH
O
N=N
O
NH
CF3
O
O
OH
13 12 11 10 9 8 7 6 5 4 3 2 1 0 -1 -2
Chemical Shift (ppm)

References

1. C. B. Cooley, B. M. Trantow, F. Nederberg, M. K. Kiesewetter, J. L. Hedrick, R. M. Waymouth and P. A. Wender, *J. Am. Chem. Soc.*, 2009, **131**, 16401-16403.
2. G. M. Burslem, H. F. Kyle, A. L. Breeze, T. A. Edwards, A. Nelson, S. L. Warriner and A. J. Wilson, *ChemBioChem*, 2014, **15**, 1083-1087.
3. P. Prabhakaran, A. Barnard, N. S. Murphy, C. A. Kilner, T. A. Edwards and A. J. Wilson, *Eur. J. Org. Chem.*, 2013, **2013**, 3504-3512.
4. P. Prabhakaran, V. Azzarito, T. Jacobs, M. J. Hardie, C. A. Kilner, T. A. Edwards, S. L. Warriner and A. J. Wilson, *Tetrahedron*, 2012, **68**, 4485-4491.
5. V. Azzarito, P. Prabhakaran, A. I. Bartlett, N. S. Murphy, M. J. Hardie, C. A. Kilner, T. A. Edwards, S. L. Warriner and A. J. Wilson, *Org. Biomol. Chem.*, 2012, **10**, 6469-6472.
6. J. P. Plante, T. Burnley, B. Malkova, M. E. Webb, S. L. Warriner, T. A. Edwards and A. J. Wilson, *Chem. Commun.*, 2009, 5091-5093.
7. J. Plante, F. Campbell, B. Malkova, C. Kilner, S. L. Warriner and A. J. Wilson, *Org. Biomol. Chem.*, 2008, **6**, 138-146.
8. D. P. Smith, J. Anderson, J. Plante, A. E. Ashcroft, S. E. Radford, A. J. Wilson and M. J. Parker, *Chem. Commun.*, 2008, 5728–5730.
9. H. F. Kyle, K. F. Wickson, Jonathon Stott, G. M. Burslem, A. L. Breeze, C. Tiede, D. C. Tomlinson, S. L. Warriner, A. Nelson, A. J. Wilson and T. A. Edwards, *Mol. BioSys.*, 2015, **11**, 2738-2749.
